# Nucleophilic Addition of 4,5-Dihydrooxazole Derivatives to Base Generated *o*-Quinone Methides: A Four-Component Reaction

**DOI:** 10.1021/acs.joc.2c02614

**Published:** 2023-01-31

**Authors:** Yuk Fai Wong, Ivan Hernandez, Thomas R. R. Pettus

**Affiliations:** Department of Chemistry and Biochemistry, University of California at Santa Barbara, Santa Barbara, California 93106-9510, United States

## Abstract

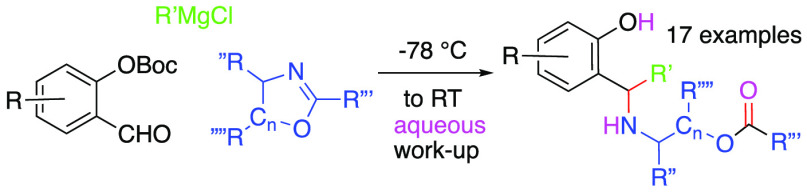

A novel method for joining *four* components together in a single pot leading to an assortment of N-amino-benzylated phenols is described. The method involves the addition of different Grignard reagents to various *o*-OBoc salicylaldehydes in the presence of assorted 4,5-dihydrooxazoles, followed by aqueous workup. Seventeen examples are presented with varied (-R, -R′ -R″, -R‴, -R⁗, and C_*n*_) substituents.

We recently described a synthetic method involving *ortho*-quinone methides (*o*-QMs), which are base generated by the addition of assorted Grignard reagents to various *ortho*-OBoc salicylaldehydes and observed to undergo reaction with the sp^2^ nitrogen atom of various imine nucleophiles and afford the corresponding 3,4-dihydro-2*H*-1,3-benzoxazines in good yields and diastereoselectivities (Scheme 1: *i*).^1^ As a multi three-component reaction (M3CR)^2^ comprised of a salicylaldehyde, a Grignard reagent, and an imine, this earlier process enables the rapid exploration of benzylic amine substrate space.^3^ Herein, we report an unexpected M4CR from replacement of the imine with dihydro-4,5-oxazole derivatives followed by hydrolytic workup of a zwitterionic intermediate.

**Scheme 1:**
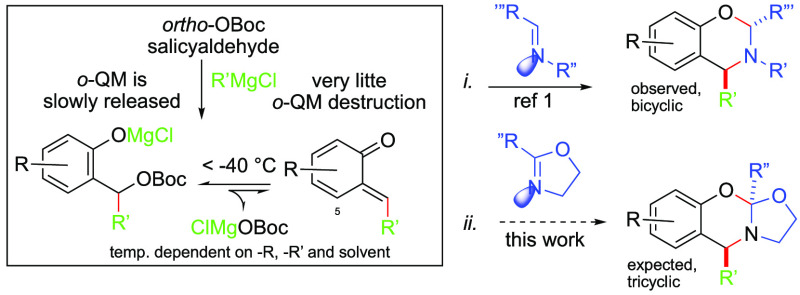
Prior Reactions and Expected Outcome

Originally, we had postulated that introduction of 4,5-dihydrooxazoles should deliver the corresponding tricyclic 1,3-benzoxazine adduct as opposed to the earlier bicyclic adducts observed for imines (Scheme 1: *ii*).^1^ When this result failed to transpire, we paused to consider the inherent reactivity of 4,5-dihydrooxazoles with electrophilic reagents (Scheme 2). We found the literature bursting with examples of cationic ring opening polymerization, and reports of block copolymer formation leading to polyamides via a pseudo-living oxazolinum terminus thermodynamically driven toward amide formation (Scheme 2: *i*).^4^ These were instigated by the addition of a small amount of an electrophilic initiator, which included an assortment of Brønsted or Lewis acids, as well as alkylation or acylation reagents under *neat* conditions. These reactions afforded poly-*N*-acylethylenimines of tunable molecular weight that were bioisosteric with polypeptides. In addition, several nonpolymerizing ring openings of dihydrooxazole have been noted at reduced temperatures. These required that the electrophile and nucleophile be introduced at a near parity of equivalents under dilute conditions. For example, after Lewis acid activation, aryl nucleophiles had been observed to add at the 5-position of the oxazolium intermediates in a diastereoselective fashion (Scheme 2: *ii*).^5^ Other ring openings included protonation with an acid displaying a weakly nucleophilic counteranion followed by the addition of a secondary amine (Scheme 2: *iii*).^6^ Upon application of ethyl chloroformate, on the other hand, the chloride anion was found to open the oxazolium ring (Scheme 2: *iv*).^7^ In addition, there was a solitary report of “wet” low-temperature conditions, whereby opportunistic water intercepted the cationic species to provide an ammonium intermediate that underwent regioselective ring opening and amine expulsion to produce an ester and ammonium species (Scheme 2: *v*)^8^ with regioselective ring opening attributed to stereoelectronic control.^9^

**Scheme 2:**
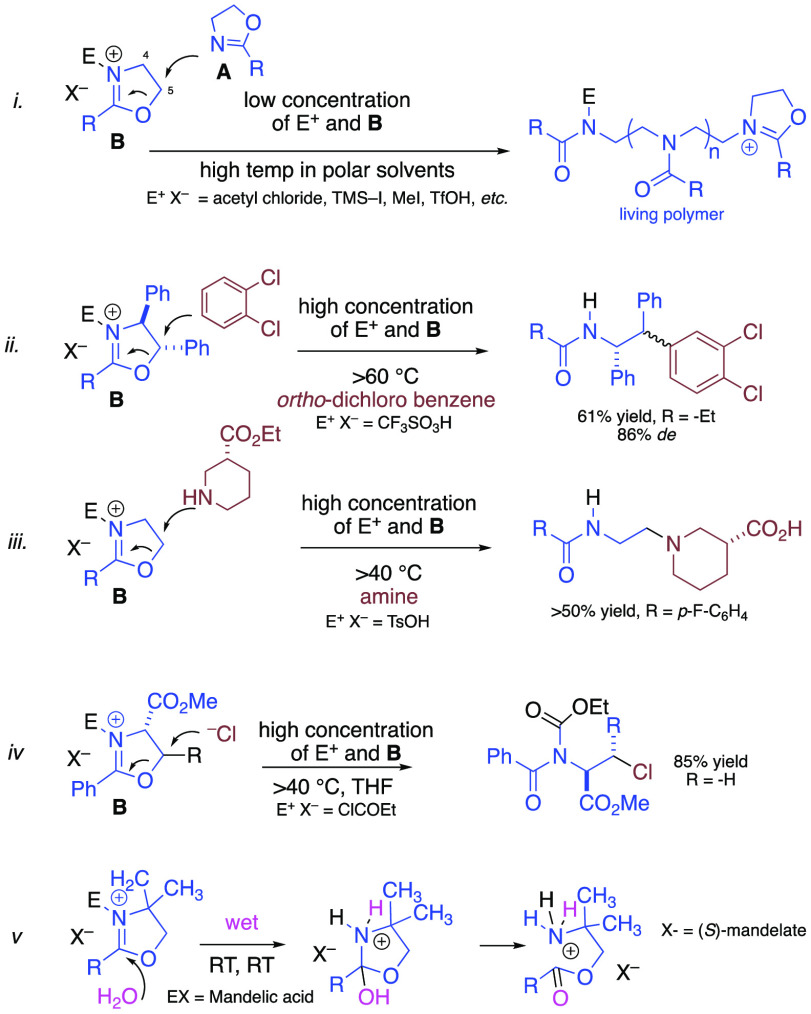
Some Reported Ring Opening Reactions of 4,5-Dihydrooxazoles

We were therefore keen to determine if any related products had arisen from our low temperature *in situ* generation of electrophilic *o*-QMs in the presence of various 4,5-dihydrooxazoles. Our analyses showed that products **25**–**41** (Table 1) had emerged from our usual conditions; addition of the Grignard reagent to the aldehyde 0.1 M in diethyl ether at −78 °C, followed by addition of the 4,5-dihydrooxazole (2 equiv) and slowly warming to RT over 24 h, followed by an *aqueous* workup with 1 M NaHCO_3._ Upon close inspection of the respective ^1^H NMR spectra, we noted that the benzylic methine resonances displayed a signal of about 4.0 ppm, whereas the corresponding benzylic amide methines generally arise at about 5.0 ppm. Thus, the ^1^H NMR spectra and the lack of rotamers revealed that the reaction had followed pathway *v* in Scheme 2, whereby opportunistic water had intercepted the oxazolium intermediate upon workup as the fourth component of a new *M4CR*.

**Table 1:**
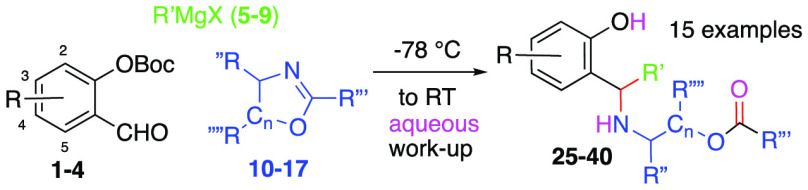

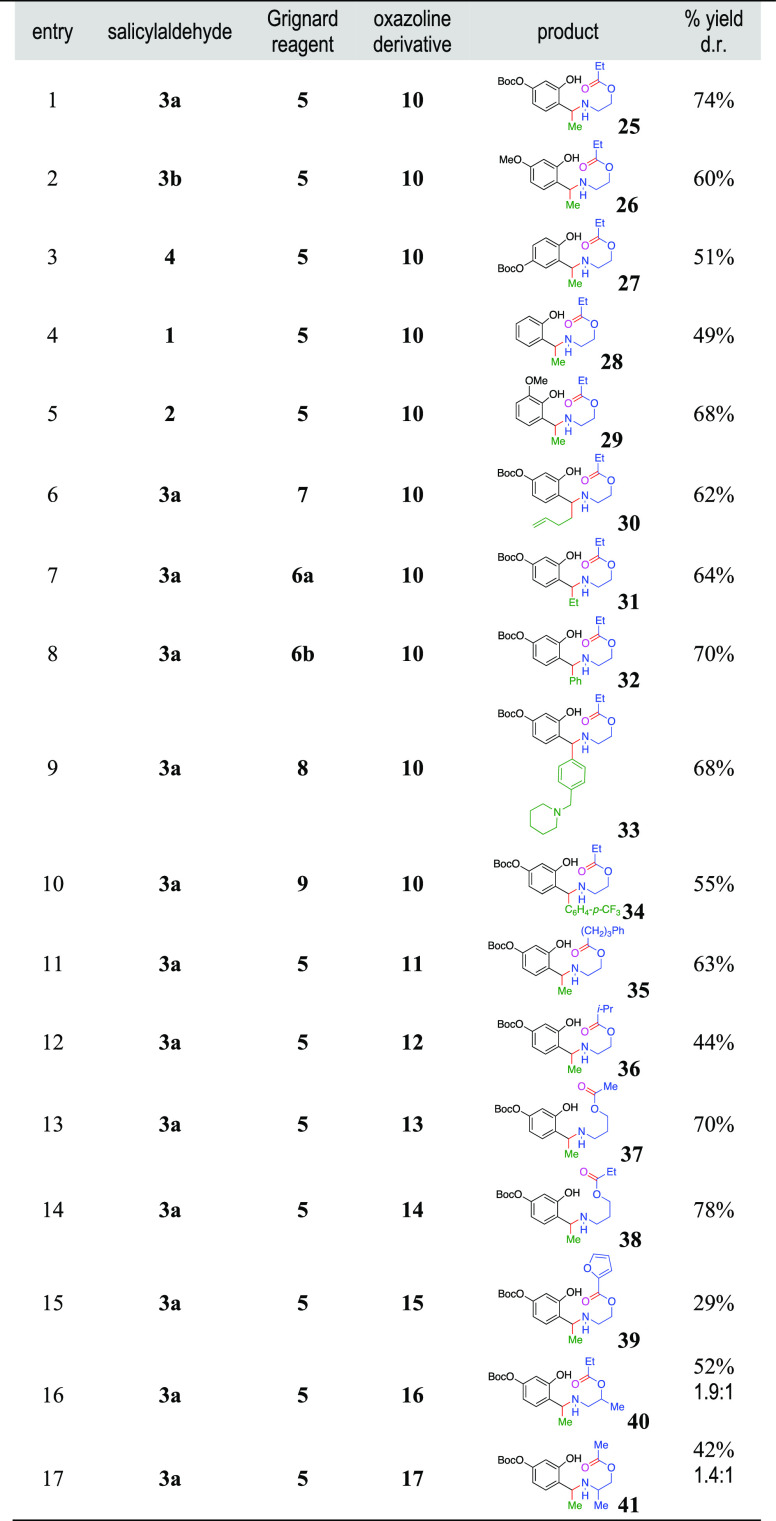
MCRs of *ortho*-OBoc Salicylic Aldehydes **1**–**4** in Combination with Various Grignard Reagents, Dihydrooxazoles, and Water

Fruitful combinations of salicylic aldehydes, Grignard reagents, and dihydrooxazoles, followed by *aqueous* bicarbonate are shown in Figure 1. The trend among yields for the aromatic cores **1**–**4** (Table 1, entries 1–5) reflected of our earlier observations in which similar *o*-QMs have been generated and intercepted by either organometallic species,^10^ alkenes,^11^ imines,^1^ or other carbon nucleophiles.^12^ Salicylaldehydes displaying electron donating substituents (C2–C4) usually provide stable *o*-QM species leading to better controlled reactions,^13^ whereas the *o*-QM derived from compound **1** (-R = -H) (entry 4, Table 1) without donating substituents resulted in moderate self-destruction and lower overall yields (entry 4, 49%).^14^ Grignard reagents **6**–**9** containing bromide (Table 1, entries 5–11) proved equally effective for *o*-QM generation as Grignard reagents containing chloride. Their reactions with aldehydes **1**–**4** and compound **10** delivered products **25**–**34** in similar yields. Variations among yields for products **35**–**41**, which arise from dihydrooxazoles **11**–**17** (Table 1, entries 10–16), as well as the ineffective examples (Figure 2, **18**–**25**) indicate several undesirable dihydrooxazole features. For example, branching at the α-position in R‴ led to poorer outcomes as did introduction of R″ and R⁗ substituents (Table 1, entries 11, 15–16; **12**, **16**–**17**). However, the reaction tolerated several straight chain R‴ substituents.

**Figure 1:**
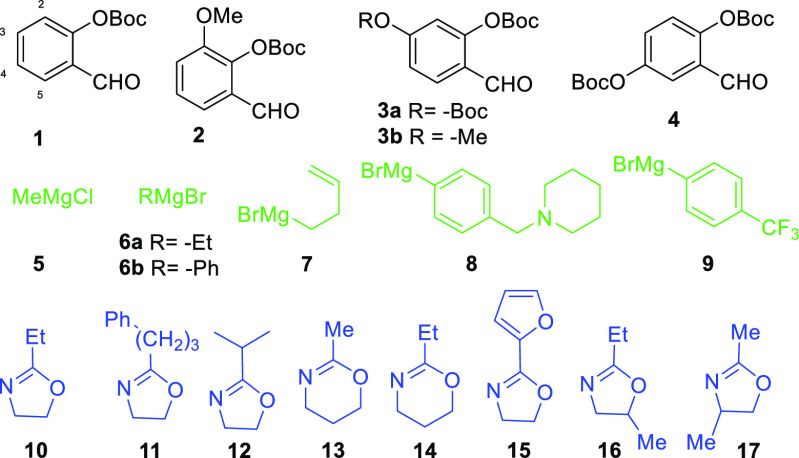
Productive reagents among MCRs tested.

**Figure 2:**
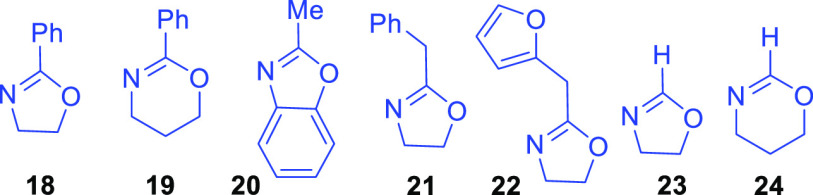
Unproductive dihydrooxazoles, oxazoles, and oxazines.

Remarkably, the furyl oxazole derivative **15** proved successful (Table 1, entry 15, 29% yield), whereas the dihydrooxazole analogues **18** and **19** (Figure 2) did not. Given that oxazole **20** also failed to provide significant product, we attribute their collective shortcomings to a combination of steric encumbrances interfering with nitrogen atom nucleophilicity, as well as an enhanced oxazolium stability and lower reactivity of the respective intermediates. These traits thwart either the initial addition of the nitrogen nucleophile, or the subsequent addition of water. We ascribed unsuccessful reactions of compounds **21**–**24** to proton acidities within their relevant oxazolium intermediates resulting in a propensity toward substrate deprotonation and destruction.

Compound **25** was observed to undergo several useful and illuminative transformations. For example, its ester moiety undergoes saponification with potassium carbonate in methanol to provide the 1° alcohol **42** (95%). Upon refluxing compound **25** at 110 °C in toluene for 48 h, we observed formation of the styrene **43** (75% yield). Lower temperatures (<60 °C) returned the starting material unchanged. We postulated this transformation proceeds by rearrangement of the ester to its corresponding amide and subsequent expulsion resulting in an *o*-QM equilibria at 90 °C, whereby the *Z* isomer participates in a 1,5-sigmatropic shift to produce styrene **43**. On the other hand, upon heating compound **25** to 90 °C in acetonitrile in the presence of imidazole (1 equiv), we observed formation of compound **44** (55%) along with styrene (<25%). The styrene likely arises from a proton transfer from within the imidazolium zwitterionic formed after imidazole addition to the *o*-QM.

Figure 3 shows our postulated mechanism and explains formation of the compounds in both Table 1 and Scheme 3. We find aldehyde **3a** and methyl Grignard **5** undergo reaction at −78 °C (0.1 M in Et_2_O) to provide the speculative cyclic intermediate **A**. This alkoxide species can collapse three possible ways. It collapses to afford the more stable phenoxide **B**, as opposed to the two other plausible albeit less stable alkoxides. Next, at some temperature between −60 to −20 °C (both R and R′ substituent dependent), the phenoxide expels the less basic *tert*-butyl carbonate sequestered as a magnesium salt to form the highly reactive *o*-QM **C**. Remarkably, lithium salts do not appear to undergo the β-elimination from **B** to **C**.^10a,15^ At these temperatures *o*-QMs with a β-methyl, as opposed to β-phenyl systems, undergo rapid self-destruction *in the absence* of a nucleophilic partner. However, in the presence of the dihydrooxazole, we surmise that it engages the *o*-QM around −20 °C to form the dihydrooxazolium zwitterion **D**, which appears more stable than intermediate **B**. It can be stored at room temperature for days, and then later redeployed as an *o*-QM around 60 °C for a variety of applications. Removal of the solvent and ^1^H NMR analysis of **D** showed no indication of the expected tricyclic 1,3-benzoxazine or the ester **25**. Instead, only intermediate **D** and the expected *t*-butyl carbonate sequestered as its magnesium salt were evident. However, upon subsequent *aqueous* workup at RT, oxazolium **D** undergoes facile addition of water at its 2-position to form the fleeting speculative intermediate **E**, which collapses to the kinetic ester **25**, rather than the more common and thermodynamically stable amide **F**.^7^ While Deslongchamps’s stereoelectronic model has been proposed to explain this hemi-*ortho* amide collapse in acyclic and six membered ring examples,^8^ we suspect formation of the ester **25** may be favored due to the basicity of the nitrogen atom and the internally available Lewis acidic proton of the phenol that facilitates amine expulsion. Remarkably, compound **25** is also another *o*-QM precursor. Upon heating compound **25** to 90 °C, we postulate that the ester moiety undergoes rearrangement to the corresponding amide **G**, which undergoes immediate and irreversible elimination of the amide alcohol to provide the *E*-*o*-QM **C**.

**Scheme 3:**
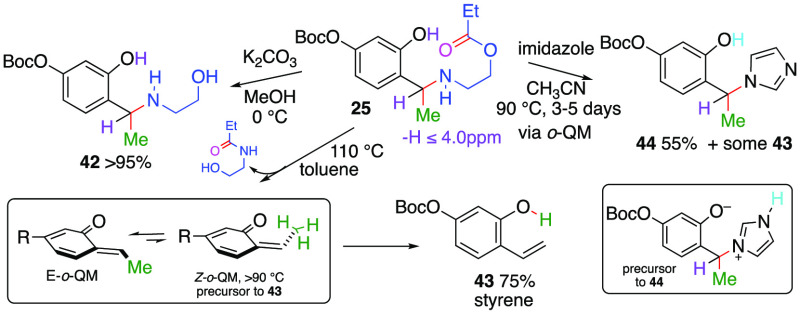
Some Transformations of Compound **25**

**Figure 3:**
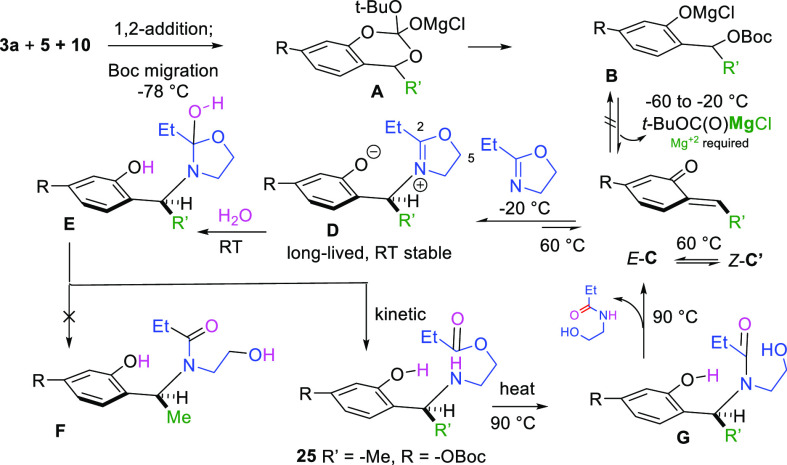
Postulated mechanism.

To strengthen these mechanistic hypotheses, we carried out the experiments shown in Scheme 4. First, we deployed our traditional low temperature cycloaddition protocol with ethoxyvinyl ether (EVE),^10^ which afforded the benzopyran **45a** as single diastereomer. This outcome supports the notion that at these low temperatures the *o*-QM *E***-C** is not in equilibrium with the *o*-QM *Z***-C**, because no styrene is observed, and *endo* diastereoselectivity for the benzopyran **45a** is outstanding. Remarkably, our attempts toward orchestrating a crossover or disrupting stereochemistry by heating **45a** (>120 °C) for 2 days failed. Thus, we concluded that **45a** is *not* an *o*-QM precursor at 120 °C. Next, we replaced EVE with dihydrooxazole **10** and carried out the same process resulting in the speculative zwitterion **D**, whereupon we introduced EVE. No formation of benzopyran **45** was apparent over the course of 24 h at RT. However, upon heating to 60 °C we isolated the benzopyran **45b** in a 1.6:1 diastereomeric ratio along with the dihydrooxazole **10**. Thus, we speculate that around 60 °C the zwitterion **D** undergoes elimination to return the *o*-QM *E*-**C** and either undergoes reaction in both *endo* and *exo* manifolds, or it exists in an equilibrium alongside the *o*-QM *Z*-**C′** which undergoes reaction in an *endo* format. However, the absences of styrene **43** supports the former notion. Lastly, we heated the amine **25** in the presence of EVE at 60 °C in a sealed tube for 2 days and observed no reaction. Thus, we surmise from the experiment that the order of stability among these *o*-QM precursors is **45a** > **25** > **44** > **D** > **B**, with all being R and R′ dependent.

**Scheme 4:**
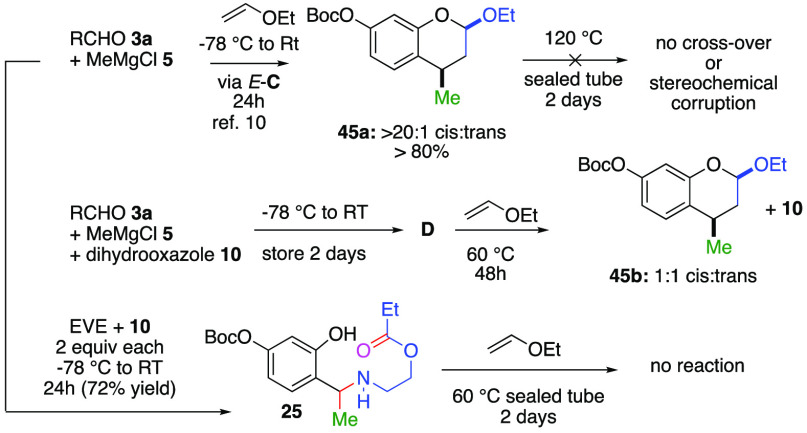
Experiments with *o*-QM Precursors

To further illuminate the regioselective collapse of intermediate **E**, we used 2-methoxy-4,5-dihydrooxazole **46** in reaction with salicylaldehyde **3a** and MeMgCl **5**. This modification results in three plausible outcomes and potentially affords compounds **47**–**49** (Scheme 5). We were surprised to find that none of the carbonate **49** had formed. Instead, the reaction provided the oxazolidinone **47** (54%) along with the carbamate **48** (19% yield). On the other hand, use of 2-(methylthio)-4,5-dihydrothiazole **50** solely gave the thiazolidin-2-one **51** in a 78% yield.

**Scheme 5:**
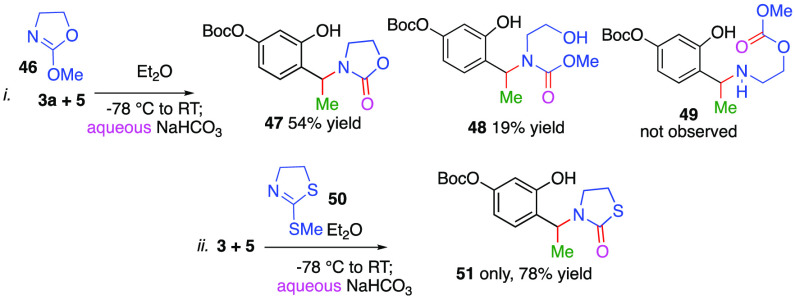
Reactions with Oxygenated and Thiolated Systems

Several natural products and their derivatives can be imagined as amenable to synthesis using this novel M(4)CR method (Figure 4). (±)-Stritida B and C (**51a**,**b**) are the first pyridocarbazole alkaloids reported to display an *N*-2-hydroxyethyl residue.^16^ (+)-Hispidacine (**52**), an 8,4′-oxyneolignan alkaloid displaying vasorelaxant activity, also manifests this motif.^17^ (±)-Irpexine (**53**), an isoindolinone alkaloid, exhibits this substituent as well.^18^ However, we chose to explore the application of this M(4)CR toward the synthesis of mariline B (**54**), a naturally occurring *racemic* phthalimidine isolated from the sponge derived fungus *Stachylidium sp*.^19^

**Figure 4:**
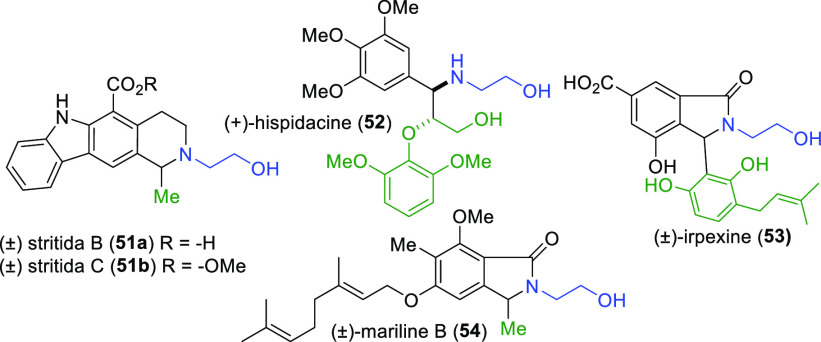
Potential natural product applications.

Construction of the isoindolinone from adduct **29** necessitated a carbonylation to replace the phenol residue and connect it with the neighboring benzylic amine. This first required conditions for selective phenol triflation in the presence of a free amine (Scheme 6). This was modestly accomplished using biphasic conditions developed by Sonesson, and it provided a sufficient yield of the corresponding triflate **55** to test our strategy.^20^ Using a modified palladium carbonylation chemistry developed by Crisp,^21^ we observed the phthalimidine to smoothly form upon exposure to carbon monoxide and palladium with the appropriate catalyst. Further *in situ* saponification afforded the desired phthalimidine **56** in 61% yield.

**Scheme 6:**
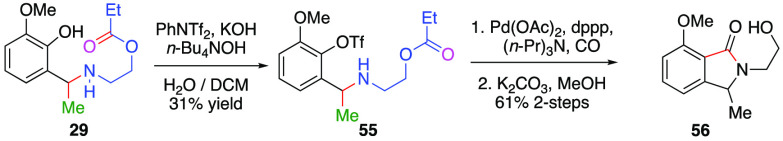
Potential Application toward (±)-Mariline B (**54**)

In conclusion, a M4CR has been developed that enables various combinations of *ortho*-OBoc salicylaldehydes, Grignards, dihydrooxazoles, and water. The method provides a large array of structurally diverse products possessing a masked *N*-2-hydroxyethyl residue. This transformation involves an unusual zwitterionic dihydrooxazole *o*-QM precursor that proves stable at RT. This unexpected species leads to the corresponding benzopyran [4 + 2] adducts without diastereoselectivity. In addition, this zwitterionic intermediate undergoes regioselective opportunistic addition of water. Moreover, we anticipate this species **D** can be selectively intercepted by other nucleophiles at either its 2- or 5- positions.^22^ Progress in this endeavor will be reported in due course.

## Data Availability

The data underlying this study are available in the published article and its Supporting Information.

## Supporting Information Available

Representative experimental procedures; Characterization data for all new compounds including ^1^H, ^13^C NMR spectra; References to other known compounds (PDF)
