# Supplementary material for: Nucleophilic Addition of 4,5-Dihydrooxazole Derivatives to Base Generated o-Quinone Methides: A Four-Component Reaction
Source: J Org Chem. 2023 Jan 31;88(4):2583–8. doi: 10.1021/acs.joc.2c02614 (PMC9942189; doi:10.1021/acs.joc.2c02614)
Supplement: Supplementary file 1 — jo2c02614_si_001.pdf [file jo2c02614_si_001.pdf]

*Supporting Information for "***Nucleophilic Addition of 4,5-Dihydrooxazole Derivatives to Base Generated o-Quinone Methides; a four component reaction**"

Yuk Fai Wong, Ivan Hernandez, Thomas R. R. Pettus *
Pettus@chem.ucsb.edu

Department of Chemistry and Biochemistry, University of California, Santa Barbara
Santa Barbara, California 93106-9510 (United States)

Table of Contents

1. General Information SI-2
2. Synthesis of Dihidrooxazole Derivatives.. SI-3
3. One-pot, Four-Component Reaction.. SI-9
4. Transformations of Adduct 25 .... SI-18
5. Experiments with *o*-QM Precursors in Scheme 4 .... SI-20
6. Adducts in Scheme 5 .... SI-23
7. Potential Application Toward Mariline B SI-25
8. NMR Spectra... SI-26

1. General Information

In reactions where water was not present as a solvent, reagent, or byproduct, the glassware was flame dried, and the reactions were carried out under an inert atmosphere of nitrogen. Reactions were monitored by analytical thin-layer chromatography on EMD silica gel 60 F254 plates; visualization was effected by ultraviolet light (254 nm), *p*-anisaldehyde or potassium permanganate stains. Solvents were removed using a rotary evaporator. If the product was non-volatile, trace solvents were removed at a reduced pressure of approximately 2 mmHg.

All purchased chemicals were used without purification unless otherwise stated. Dichloromethane was distilled from CaH_2. Diethyl ether, tetrahydrofuran, and toluene were distilled from sodium and benzophenone. Deuterated chloroform was stored over anhydrous potassium carbonate and 4Å molecular sieves before use.

^{1}H NMR spectra were recorded at Varian or Bruker, 500 or 600 MHz instruments with the solvent resonance of $CDCl_3$ (7.26 ppm) and CD_2Cl_2 (5.32 ppm). Coupling constants (*J*) are reported in Hz and splitting patterns are designated using the following abbreviations: s (singlet), d (doublet), t (triplet), q (quartet), quin (quintet), sex (sextet), m (multiplet), br (broad). ^{13}C NMR spectra were recorded at 500 or 600 MHz instruments with a solvent resonance of $CDCl_3$ (77.0 ppm). High resolution mass spectra (HRMS) were obtained by electrospray ionization/time-of-flight experiments performed by Mass Spectrometry Facility, University of California Irvine.

2. Synthesis of Dihidrooxazole Derivatives

General Procedure A

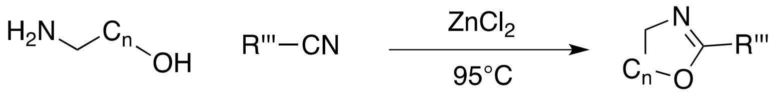

At room temperature, anhydrous $ZnCl_2$ (140 mg, 1 mmol, 2 mol%) was suspended in anhydrous nitrile (55 mmol, 1.1 equiv). To this suspension, aminoalcohol (50 mmol, 1.0 equiv) was added dropwise with stirring. The mixture was heated at 95 °C for 48 hours. The crude mixture was purified by vacuum distillation to afford pure product. The product was further dried by another vacuum distillation over CaH_2 before use.

General Procedure B

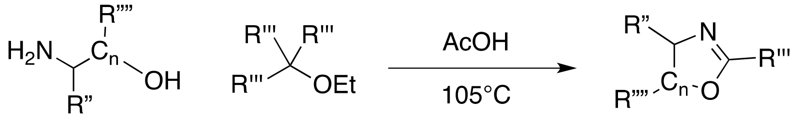

A sealed tube was charged with a mixture of aminoalcohol (20 mmol, 1.0 equiv), triethyl orthoester (24 mmol, 1.2 equiv) and glacial acetic acid (70 μL, 1.2 mmol, 6 mol%) in DCE (20 mL). The tube was heated at 95 °C for 16 hours. The mixture was cooled to room temperature, quenched with saturated $NaHCO_3$ solution (10 mL), extracted with dichloromethane (4 × 10 mL). Organic phases were combined, dried over anhydrous Na_2SO_4, and concentrated *in vacuo*. The crude product was purified by vacuum distillation over CaH_2 to afford pure, anhydrous product.

General Procedure C

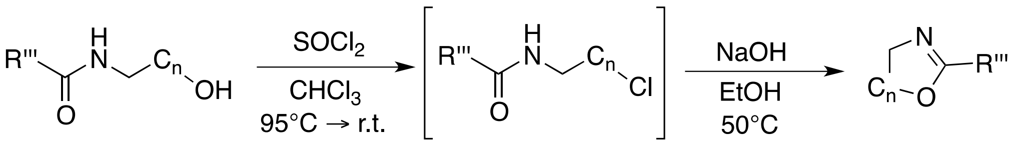

A solution of amido alcohol (15.0 mmol) in dry chloroform (80 mL) was cooled to 0 °C. $SOCl_2$ (1.85 mL, 25.5 mmol, 1.7 equiv) was added dropwise using a dropping funnel. The mixture was warmed to room temperature and stirred for 16 hours. The reaction was quenched with 1M Na_2CO_3 solution (20 mL), extracted with chloroform (3 × 10 mL). Organic phases were combined, dried over anhydrous $MgSO_4$, and concentrated *in vacuo*. The crude chloride was taken up in dry ethanol (15 mL). Pellets of NaOH (3.61 g, 90.0 mmol, 6.0 equiv) was then added. The mixture was heated at 50 °C for 16 hours. The white salt precipitated was removed by filtering through a pad of Celite. The filtrate was concentrated *in vacuo*, then taken up in chloroform (40 mL). The solution was washed with water (10 mL) and brine (10 mL), dried over anhydrous Na_2SO_4, and concentrated. The crude product was distilled over CaH_2 to afford pure, anhydrous product.

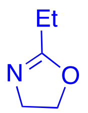

10

2-Ethyl-4,5-dihydrooxazole (10). Obtained from Alfa Aesar. It was further dried by vacuum distilled over CaH_2 (b.p. = 65 °C at 60 Torr) before use.

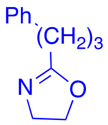

11

2-(3-Phenylpropyl)-4,5-dihydrooxazole (11). Prepared according to the general procedure C. The crude product was purified by vacuum distillation (b.p. = 74 °C at Torr) as a colorless oil (0.79 g, 28% isolated yield). **^{1}H NMR** (500 MHz, $CDCl_3$): δ 7.24 - 7.30 (m, 3H), 7.15 - 7.21 (m, 2H), 4.20 (t, *J* = 9.5 Hz, 2H), 3.77 - 3.85 (m, 2H), 2.68 (t, *J* = 7.7 Hz, 2H), 2.30 (t, *J* = 7.5 Hz, 2H), 1.97 (quin, *J* = 7.6 Hz, 2H). Our characterization data match with prior literature data.[1]

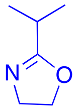

12

2-Isopropyl-4,5-dihydrooxazole (12). Prepared according to the general procedure A. The crude product was purified by vacuum distillation (b.p. = 68 °C at Torr) as a colorless oil (1.70 g, 30% isolated yield). **^{1}H NMR** (500 MHz, $CDCl_3$): δ 4.21 (t, *J* = 9.5 Hz, 2H), 3.81 (t, *J* = 9.3 Hz, 2H), 2.56 (sept, *J* = 7.0 Hz, 1H), 1.19 (d, *J* = 7.0 Hz, 6H). Our characterization data match with prior literature data.[2]

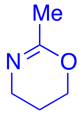

13

2-Methyl-5,6-dihydro-4H-1,3-oxazine (13). Prepared according to the general procedure A. The crude product was purified by vacuum distillation (b.p. = 65 °C at 60 Torr) as a colorless oil (1.98 g, 40% isolated yield). **^{1}H NMR** (600 MHz, $CDCl_3$): δ 4.13 (br s, 2H), 3.32 (br s, 2H), 1.88 (s, 3H) 1.84 (t, *J* = 5.4 Hz, 2H). Our characterization data match with prior literature data.[3]

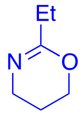

14

2-Ethyl-5,6-dihydro-4H-1,3-oxazine (14). Prepared according to the general procedure A. The crude product was purified by vacuum distillation (b.p. = 78 °C at ~60 mmHg) as a colorless oil (3.17 g, 56% isolated yield). **^{1}H NMR** (600 MHz, $CDCl_3$): δ 4.13 (t, *J* = 5.4 Hz, 2H), 3.35 (t, *J* = 5.7 Hz, 2H), 2.13 (q, *J* = 7.7 Hz, 2H), 1.84 (quin, *J* = 5.7 Hz, 2H), 1.08 (t, *J* = 7.7 Hz, 3H). Our characterization data match with prior literature data.[4]

[1] Huang, X.; Zhao, W.; Chen, D.-L.; Zhan, Y.; Zeng, T.; Jin, H.; Peng, B. *Chem. Commun.* **2019**, *55* (14), 2070-2073.

[2] Meyer, M.; Schlaad, H. *Macromolecules* **2006**, *39* (11), 3967-3970.

[3] Pang, S. H.; Lively, R. P.; Jones, C. W. *ChemSusChem* **2018**, *11* (15), 2628-2637.

[4] Papadopoulos, E. P.; George, B. *J. Org. Chem.* **1977**, *42* (14), 2530-2532.

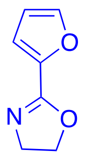

15

2-(Furan-2-yl)-4,5-dihydrooxazole (15). Prepared according to the general procedure A. The crude product was purified by recrystallization in ethyl acetate and hexanes as white solid (2.19 g, 32% isolated yield). **^{1}H NMR** (400 MHz, $CDCl_3$): δ 7.54 (d, *J* = 1.2 Hz, 1H), 6.95 (d, *J* = 3.1 Hz, 1H), 6.48 (dd, *J* = 3.5, 2.0 Hz, 1H), 4.41 (t, *J* = 9.4 Hz, 2H), 4.06 (t, *J* = 9.6 Hz, 2H). Our characterization data match with prior literature data.[5]

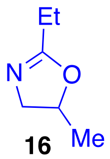

16

2-Ethyl-5-methyl-4,5-dihydrooxazole (16). Prepared according to the general procedure A. The crude product was purified by vacuum distillation (b.p. = 70 °C at 60 Torr) as a colorless oil (1.75 g, 31% isolated yield). **^{1}H NMR** (400 MHz, $CDCl_3$): δ 4.70-4.55 (m, 1H), 3.90 (dd, *J* = 13.7, 9.4 Hz, 1H), 3.36 (dd, *J* = 13.9, 7.2 Hz, 1H), 2.26 (q, *J* = 7.2 Hz, 2H), 1.31 (d, *J* = 6.7 Hz, 3H), 1.17 (t, *J* = 7.4 Hz, 3H). Our characterization data match with prior literature data of (*R*) and (*S*) enantiomers.[6]

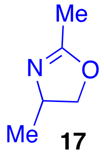

17

2,4-Dimethyl-4,5-dihydrooxazole (17). Prepared according to the general procedure B. The crude product was purified by vacuum distillation (b.p. = 65 °C at 60 Torr) as a colorless oil (0.137 g, 5.1% isolated yield). **^{1}H NMR** (500 MHz, $CDCl_3$): δ 4.33 (t, *J* = 9 Hz, 1H) 4.16 - 4.10 (m, 1H), 3.76 (t, *J* = 8 Hz, 1H) 1.97 (s, 3H) 1.24 (d, *J* = 6.5 Hz, 3H). Our characterization data match with prior literature data.[7]

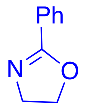

18

2-Phenyl-4,5-dihydrooxazole (18). Prepared according to the general procedure A. The crude product was purified by vacuum distillation (b.p. = 95 °C at 2 Torr) as a colorless oil (3.61 g, 49% isolated yield). The oil solidified into white solid upon standing at room temperature. **^{1}H NMR** (500 MHz, $CDCl_3$): δ 8.00-7.88 (m, 2 H), 7.52-7.44 (m, 1H), 7.44-7.37 (m, 2H), 4.44 (t, *J* = 9.6 Hz, 2H), 4.06 (t, *J* = 9.6 Hz, 2H). Our characterization data match with prior literature data.[8]

[5] Zhu, J.; Zhou, M.; Jiang, W.; Zhou, Y.; Song, G.; Liu, R. *Tetrahedron Lett.* **2022**, *91*, 153637.

[6] Luxenhofer, R.; Huber, S.; Hytry, J.; Tong, J.; Kabanov, A. V.; Jordan, R. *Journal of Polymer Science Part A: Polymer Chemistry* **2012**, *51* (3), 732-738.

[7] Herman, H. H.; Husain, P. A.; Colbert, J. E.; Schweri, M. M.; Pollock, S. H.; Fowler, L. C.; May, S. W. *J. Med. Chem.* **1991**, 34, 3, 1082–1085.

[8] Rodriguez del Rey, F. O.; Floreancig, P. E. *Org. Lett.* **2021**, *23* (1), 150-154.

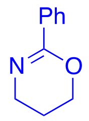

19

2-Phenyl-5,6-dihydro-4H-1,3-oxazine (19). Prepared according to the general procedure A. The crude product was purified by vacuum distillation (b.p. = 120 °C at 2 mmHg) as a colorless oil (3.06 g, 38% isolated yield). **^{1}H NMR** (600 MHz, $CDCl_3$): δ 7.90-7.86 (m, 2H), 7.42-7.38 (m, 1H), 7.38-7.33 (m, 2H), 4.36 (t, *J* = 5.6 Hz, 2H), 3.61 (t, *J* = 5.9 Hz, 2H), 1.98 (quin, *J* = 5.7 Hz, 2H). Our characterization data match with prior literature data.[9]

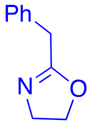

21

2-Benzyl-4,5-dihydrooxazole (21). Prepared according to the general procedure C. The crude product was purified by vacuum distillation (b.p. = 100 °C at 2 mmHg) as a colorless oil (1.18 g, 49% isolated yield). **^{1}H NMR** (400 MHz, $CDCl_3$): δ 7.40-7.14 (m, 5H), 4.24 (t, *J* = 9.8 Hz, 2H), 3.84 (t, *J* = 9.6 Hz, 2H), 3.62 (s, 2H). Our characterization data match with prior literature data.[10]

22 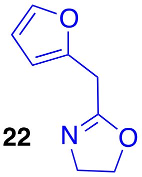

2-(Furan-2-ylmethyl)-4,5-dihydrooxazole (22). Prepared according to the general procedure C. The crude product was purified by vacuum distillation (b.p. = 95 °C at 2 mmHg) as a colorless oil (0.40 g, 16% isolated yield). **^{1}H NMR** (500 MHz, $CDCl_3$): δ 7.31 (dd, *J* = 1.8, 0.8 Hz, 1H), 6.28 (dd, *J* = 3.1, 1.8 Hz, 1H), 6.06-6.01 (m, 1H), 4.26 (td, *J* = 9.5, 2.1 Hz, 2H), 3.85 (t, *J* = 9.0 Hz, 2H), 3.00 (s, 2H). Our characterization data match with prior literature data.[11]

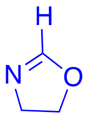

23

4,5-Dihydrooxazole (23). Prepared according to the general procedure C. The crude product was purified by distillation (b.p. = 96 °C at 760 mmHg) as an orange oil (50 mg, 5% isolated yield). **^{1}H NMR** (600 MHz, CD_3OD): 8.07 (s, 1H), 3.61 (t, *J* = 5.4 Hz, 2H) 3.58 (t, *J* = 5.4 Hz, 2H). Our characterization data match with prior literature data.[12]

[9] Lin, S.; Sheng, X.; Zhang, X.; Liu, H.; Luo, C.; Hou, S.; Li, B.; Chen, X.; Li, Y.; Xie, F. *J. Org. Chem.* **2021**, *87* (2), 1366-1376.

[10] Takasu, A.; Kojima, H. *Journal of Polymer Science Part A: Polymer Chemistry* **2010**, *48* (24), 5953–5960

[11] Soleymani Movahed, F.; Foo, S. W.; Mori, S.; Ogawa, S.; Saito, S. *J. Org. Chem.* **2022**, *87*, 243-257.

[12] Tauhardt, L.; Kempe, K.; Schubert, U. S. *Journal of Polymer Science Part A: Polymer Chemistry* **2012**, *50* (21), 4516–4523.

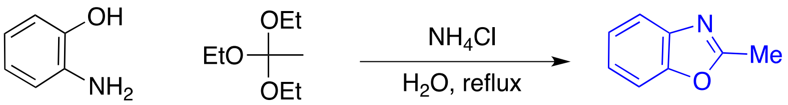

20

2-Phenyl-5,6-dihydro-4H-1,3-oxazine (20). 2-Aminophenol (5.00 g, 46 mmol) was suspended in a solution of triethyl orthoacetate (7.80 g, 48.1 mmol, 1.05 equiv.) and ammonium chloride (367 mg, 3.87 mmol, 15 mol%) in water (100 mL). The mixture was refluxed for 16 hours. After cooling to room temperature, the aqueous layer was extracted with ethyl acetate (3 × 70 mL). Organic phases were combined, washed with brine (50 mL), dried over anhydrous Na_2SO_4, and concentrated *in vacuo*. The residue was first purified by column chromatography (SiO_2, eluent: hexanes/ethyl acetate = 3:1), then dried by vacuum distillation over CaH_2 (b.p. = 75°C under 2 torr) as yellow oil (1.41 g, 23% isolated yield). **^{1}H NMR** (500 MHz, $CDCl_3$): δ 7.68-7.62 (m, 1H), 7.49-7.44 (m, 1H), 7.32-7.27 (m, 2H), 2.64 (s, 3H). **R_f** = 0.6 (hexanes/ethyl acetate = 1:1). Our characterization data match with prior literature data.[13]

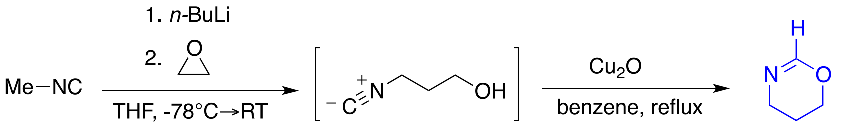

24

5,6-Dihydro-4*H*-1,3-oxazine (24). A solution of isocyanomethane (2.40 g, 58.4 mmol) in THF (60 mL) was cooled to -78°C. To this solution, *n*-BuLi (33.6 mL, 1.7M solution in pentane, 58.4 mmol) was added dropwise, followed by a solution of oxirane (29.4 mL, 2.0M solution in THF, 58.4 mmol). The mixture was warmed to 0°C for 10 minutes. The reaction was quenched by adding glacial acetic acid (3.6 mL) at -20°C. The mixture was warmed to room temperature, and concentrated in vacuo. The resulting residue was dissolved in biphasic mixture of DCM (300 mL) and water (30 mL). The aqueous layer was extracted with DCM (3 × 100 mL). The combined organic layer was dried over anhydrous Na_2SO_4 and concentrated. The residue was taken up in benzene (40 mL), then Cu_2O (12 mg, 0.558 mmol, 1 mol%) was added. The resulting suspension was refluxed for 16 hours. Upon cooling to room temperature, the mixture was filtered. The filtrate collected was purified by fractional distillation (b.p. = 120°C under 760 torr) to afford the pure product as colorless oil (240 mg, 5% isolated yield). **^{1}H NMR** (600 MHz, $CDCl_3$): δ 7.27 (s, 1H), 4.31 (t, *J* = 5.9 Hz, 2H), 3.26 (t, *J* = 5.6 Hz, 2H), 2.03 (m, 2H). Our characterization data match with prior literature data.[14]

[13] Kummari, V. B.; Chiranjeevi, K.; Suman Kumar, A.; Kumar, R. A.; Yadav, J. S. *Syn. Comm.* **2019**, *49* (23), 3335–3342

[14] Deslongchamps, P.; Cheriyan, U. O.; Taillefer, R. J. *Canadian Journal of Chemistry* **1979**, *57* (24), 3262–3271.

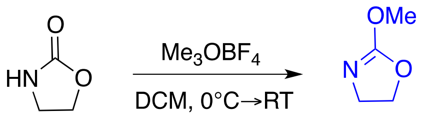

46

2-Methoxy-4,5-dihydrooxazole (46). Trimethyloxonium tetrafluoroborate (5.61 g, 38 mmol) was suspended in dry dichloromethane (15 mL) at 0°C. A solution of oxazolidin-2-one (3.00 g, 34.5 mmol) in dichloromethane (15 mL) was added. The mixture was stirred at room temperature for 16 hours before quenching with cold aqueous sodium carbonate solution. The aqueous layer was extracted with dichloromethane (3 × 20 mL). Organic phases were combined, dried over anhydrous Na_2SO_4, and concentrated *in vacuo*. The residue was purified by distillation over CaH_2 (b.p. = 97°C under 760 torr) as colorless oil (1.58 g, 45% isolated yield). **^{1}H NMR** (400 MHz, $CDCl_3$): δ 4.41 (t, J = 8.8 Hz, 2H), 3.87 (s, 3H), 3.80 (t, J = 8.8 Hz, 2H). **^{13}C{^{1}H} NMR** (126 MHz, $CDCl_3$): δ 164.5, 69.0, 57.5, 51.6. **HRMS (ESI)** Not stable in HRMS condition.

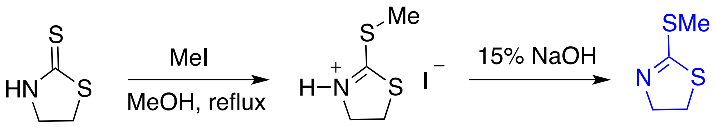

50

2-(Methylthio)-4,5-dihydrothiazole (50). To a solution of thiazolidine-2-thione (2.85 g, 24 mmol) in methanol (15 mL) was added methyl iodide (3.39 g, 24 mmol). The mixture was refluxed for 1 hour. After cooling to room temperature, diethyl ether was added slowly. White crystalline solid precipitated was collected by vacuum filtration. The solid was dissolved in 15% NaOH solution (24 mL), and extracted with chloroform (3 × 20 mL). Organic phases were combined, dried over anhydrous Na_2SO_4, and concentrated *in vacuo*. The residue was purified by distillation over CaH_2 (b.p. = 54°C under 2 torr) as colorless oil (1.60 g, 50% isolated yield). **^{1}H NMR** (600 MHz, $CDCl_3$): δ 4.23 (t, J = 8.0 Hz, 2H), 3.43 (t, J = 8.0 Hz, 2H), 2.58 (s, 3H). Our characterization data match with prior literature data.[15]

[15] Nain Singh, K.; Singh, P.; Kaur, A. *Syn. Comm.* **2006**, *36* (22), 3339-3343.

3. One-pot, Four-Component Reaction

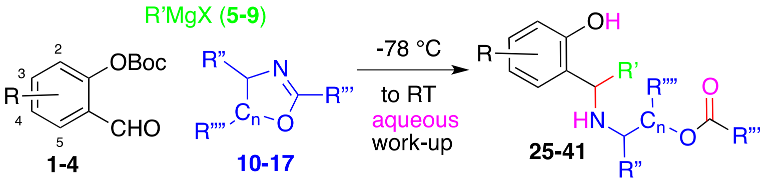

General Procedure D

Preparation of Dihydrooxazole Derivatives and o-Boc Aldehydes Solutions

Anhydrous dihydrooxazole derivatives and *o*-OBoc aldehydes[16] were diluted as 1.0 M solution in toluene before use. Under nitrogen atmosphere, a flame-dried round-bottomed flask was charged with pure dihydrooxazole or aldehyde. They were dissolved in appropriate amount of dry toluene to make up a 1.0 M of solution. These solutions were dried over 4 Å molecular sieves overnight before use.

*Preparation of Grignard Reagents **5-9***

Grignard reagents **5** (3.0 M solution in THF) and **6a-b** (2.0 M solutions in THF) were obtained from Aldrich. Grignard reagents **7**-**9** were prepared from its corresponding bromides as follows. A Schlenk flask was charged with magnesium turnings (243 mg, 10 mmol) and a piece of iodine crystal under N_2 atmosphere. A solution of aryl or alkyl bromide (10 mmol) in THF (10 mL) was added dropwise with vigorous stirring at room temperature. The mixture was stirred at 50ºC for another hour. Upon cooling back to room temperature, the solution was stored in dark under nitrogen atmosphere. The concentrations of all Grignard reagents were determined by titration[17] before use.

One-pot, Four-Component Reaction via ortho-Quinone Methides

To a flame-dried Schlenk tube was charged with *o*-OBoc aldehyde solution (0.1 mL, 0.1 mmol, 1.0 M in toluene) and dry Et_2O (1 mL). Grignard reagent (1.05 equiv) was added to the mixture at -78ºC. The mixture was allowed to stir at the same temperature for 10 minutes before addition of dihydrooxazole solution (0.2 mL, 0.2 mmol, 1.0 M in toluene, 2 equiv.). The mixture was allowed to warm to room temperature over 16 hours. The solution was quenched with saturated $NaHCO_3$ solution (1 mL), extracted with Et_2O (3 × 1mL). Organic layers were combined, washed with brine (1 mL), dried over anhydrous Na_2SO_4, and concentrated. The crude product was purified by column chromatography (SiO_2, eluent: hexanes/ethyl acetate = 5:1→1:1) to afford pure products.

[16] Aldehydes **1, 3a, 4** were prepared following our previous reported procedure, see Jones, R. M.; Van De Water, R. W.; Lindsey, C. C.; Hoarau, C.; Ung, T.; Pettus, T. R. R. *J. Org. Chem.* **2001**, *66*, 3435-3441. Other aldehydes were prepared similarly, and have been reported in following references. Compound **2**: Gulotty, E. M.; Rodriguez, K. X.; Parker, E. E.; Ashfeld, B. L. *Eur. J. Chem.***2021**, *27* (40), 10349-10355. Compound **3b**: Marsini, M. A.; Huang, Y.; Van De Water, R. W.; Pettus, T. R. R. *Org. Lett.* **2007**, *9* (17), 3229-3232.

[17] Lin, H.-S.; Paquette, L. A. *Synth. Commun.* **1994**, *24* (17), 2503-2506.

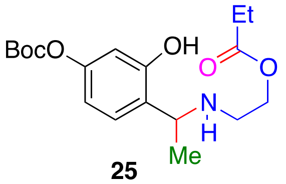

25

2-((1-(4-((*Tert*-butoxycarbonyl)oxy)-2-hydroxyphenyl)ethyl)amino)ethyl propionate (25). Prepared according to the general procedure D (purified by column chromatography, eluent: hexanes/ethyl acetate = 5:1→1:1). Yellow oil (26.1 mg, 74% isolated yield). **^{1}H NMR** (600 MHz, $CDCl_3$): δ 6.91 (d, *J* = 8.0 Hz, 1H), 6.61 (d, *J* = 2.4 Hz, 1H), 6.58 (dd, *J* = 8.0, 2.4 Hz, 1H), 4.26 (ddd, *J* = 11.7, 6.4, 3.5 Hz, 1H), 4.11 (ddd, *J* = 11.3, 7.1, 3.8 Hz, 1H), 3.94 (q, *J* = 6.8 Hz, 1H), 2.92-2.80 (m, 2H), 2.37 (q, *J* = 7.7 Hz, 2H), 1.54 (s, 9H), 1.44 (d, *J* = 6.6 Hz, 3H), 1.15 (t, *J* = 7.7 Hz, 3H). **^{13}C{^{1}H} NMR** (126 MHz, $CDCl_3$): δ 174.3, 158.1, 151.9, 151.3, 128.4, 123.7, 111.9, 110.0, 83.3, 62.9, 58.2, 45.9, 27.7, 27.4, 22.3, 9.0. **IR** (neat, cm^{-1}): 3318, 2978, 2931, 2839, 1739, 1600, 1461. **HRMS (ESI)** m/z calculated for $C_{18}H_{28}NO_6$ $[M+H]^+$: 354.1917; found 354.1923 **R_f** = 0.2 (hexanes/ethyl acetate = 3:1).

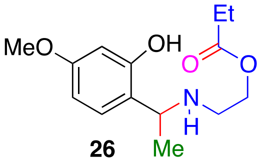

26

2-((1-(2-Hydroxy-4-methoxyphenyl)ethyl)amino)ethyl propionate (26). Prepared according to the general procedure D (purified by column chromatography, eluent: hexanes/ethyl acetate = 5:1→1:1). Yellow oil (16.0 mg, 60% isolated yield). **^{1}H NMR** (600 MHz, $CDCl_3$): δ 6.84 (d, *J* = 8.3 Hz, 1H), 6.39 (d, *J* = 2.4 Hz, 1H), 6.35 (dd, *J* = 8.3, 2.4 Hz, 1H), 4.27 (ddd, *J* = 11.4, 6.5, 3.7 Hz, 1H), 4.12 (ddd, *J* = 11.3, 7.1, 3.8 Hz, 1H), 3.92 (q, *J* = 6.6 Hz, 1H), 3.75 (s, 3H), 2.94-2.79 (m, 2 H), 2.37 (q, *J* = 7.5 Hz, 2H), 1.44 (d, *J* = 6.6 Hz, 3H), 1.16 (t, *J* = 7.5 Hz, 3H). **^{13}C{^{1}H} NMR** (126 MHz, $CDCl_3$): δ 174.4, 160.2, 158.2, 128.7, 118.4, 105.2, 102.2, 62.9, 58.0, 55.2, 45.7, 27.4, 22.4, 9.0. **IR** (neat, cm^{-1}): 3314, 2988, 2870, 1736, 1619, 1591, 1508, 1462. **HRMS (ESI)** m/z calculated for $C_{14}H_{21}NO_4Na$ $[M+Na]^+$: 290.1368; found 290.1377. **R_f** = 0.2 (hexanes/ethyl acetate = 3:1).

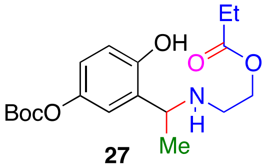

27

2-((1-(5-((*Tert*-butoxycarbonyl)oxy)-2-hydroxyphenyl)ethyl)amino)ethyl propionate (27). Prepared according to the general procedure D (purified by column chromatography, eluent: hexanes/ethyl acetate = 3:1→1:1). Yellow oil (18.0 mg, 51% isolated yield). **^{1}H NMR** (600 MHz, $CDCl_3$): δ 6.93 (dd, *J* = 8.9, 3.0 Hz, 1H), 6.78 (d, *J* = 2.8 Hz, 1H), 6.77 (d, *J* = 9.0 Hz, 1H), 4.26 (ddd, *J* = 11.4, 6.3, 3.5 Hz, 1H), 4.12 (ddd, *J* = 11.5, 7.3, 3.5 Hz, 1H), 3.92 (q, *J* = 6.8 Hz, 1H),

2.95-2.79 (m, 2H), 2.37 (q, *J* = 7.7 Hz, 2H), 1.54 (s, 9H), 1.46 (d, *J* = 6.6 Hz, 3H), 1.16 (t, *J* = 7.5 Hz, 3H). **^{13}C{^{1}H} NMR** (126 MHz, $CDCl_3$): δ 174.3, 154.7, 152.4, 143.4, 126.5, 121.0, 120.6, 117.3, 83.2, 62.9, 58.4, 46.0, 27.7, 27.4, 22.0, 9.0. **HRMS (ESI)** m/z calculated for $C_{18}H_{28}NO_6$ $[M+H]^+$: 354.1917; found 354.1923. **R$_f$** = 0.1 (hexanes/ethyl acetate = 3:1).

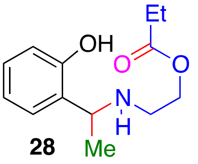

28

2-((1-(2-Hydroxyphenyl)ethyl)amino)ethyl propionate (28). Prepared according to the general procedure D (purified by column chromatography, eluent: hexanes/ethyl acetate = 5:1→1:1). Yellow oil (11.6 mg, 49% isolated yield). **^{1}H NMR** (600 MHz, $CDCl_3$): δ 7.14 (td, *J* = 8.0, 1.7 Hz, 1H), 6.95 (dd, *J* = 7.5, 1.2 Hz, 1H), 6.81 (d, *J* = 8.0 Hz, 1H), 6.77 (td, *J* = 7.4, 0.9 Hz, 1H), 4.27 (ddd, *J* = 11.6, 6.5, 3.5 Hz, 1H), 4.12 (ddd, *J* = 11.4, 7.2, 3.7 Hz, 1H), 3.96 (q, *J* = 6.7 Hz, 1H), 2.94-2.80 (m, 2H), 2.37 (q, *J* = 7.5 Hz, 2H), 1.47 (d, *J* = 7.0 Hz, 3H), 1.16 (t, *J* = 7.5 Hz, 3H). **^{13}C{^{1}H} NMR** (126 MHz, $CDCl_3$): δ 174.4, 157.1, 128.5, 128.1, 126.1, 119.2, 116.8, 62.9, 58.7, 45.9, 27.4, 22.3, 9.0. **HRMS (ESI)** m/z calculated for $C_{13}H_{19}NO_3Na$ $[M+Na]^+$: 260.1263; found 260.1259. **R$_f$** = 0.3 (hexanes/ethyl acetate = 3:1).

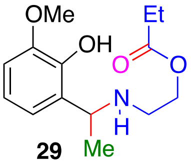

29

2-((1-(2-Hydroxy-3-methoxyphenyl)ethyl)amino)ethyl propionate (29). Prepared according to the general procedure D (purified by column chromatography, eluent: hexanes/ethyl acetate = 3:1→1:1). Yellow oil (18.2 mg, 68% isolated yield). **^{1}H NMR** (600 MHz, $CDCl_3$): δ6.78 (dd, *J*=8.0, 1.4 Hz, 1 H), 6.74 (t, *J* = 7.8 Hz, 1H), 6.59 (dd, *J* = 7.7, 1.4 Hz, 1H), 4.28 (ddd, *J* = 11.5, 5.9, 3.8 Hz, 1H), 4.11 (ddd, *J* = 11.6, 7.4, 4.0 Hz, 1H), 3.97 (q, *J* = 6.8 Hz, 1H), 3.87 (s, 3H), 2.94-2.79 (m, 2H), 2.37 (q, *J* = 7.5 Hz, 2H), 1.47 (d, *J* = 7.0 Hz, 3H), 1.15 (t, *J* = 7.7 Hz, 3H). **^{13}C{^{1}H} NMR** (126 MHz, $CDCl_3$): δ 174.3, 148.2, 146.3, 126.5, 120.1, 118.8, 110.6, 63.0, 58.5, 55.8, 46.0, 27.4, 22.3, 9.0. **HRMS (ESI)** m/z calculated for $C_{14}H_{22}NO_4$ $[M+H]^+$: 268.1549; found 268.1547. **R$_f$** = 0.3 (hexanes/ethyl acetate = 1:1).

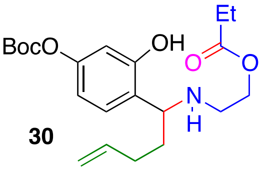

30

2-((1-(4-((*Tert*-butoxycarbonyl)oxy)-2-hydroxyphenyl)pent-4-en-1-yl)amino)ethyl propionate

(30). Prepared according to the general procedure D (purified by column chromatography, eluent: hexanes/ethyl acetate = 5:1→1:1). Yellow oil (24.4 mg, 62% isolated yield). **^{1}H NMR** (600 MHz, $CDCl_3$): δ 6.91 (d, *J* = 8.0 Hz, 1H), 6.70 (br s, 1H), 6.61 (dd, *J* = 8.2, 2.3 Hz, 1H), 5.76 (ddt, *J* = 17.0, 10.4, 6.5 Hz, 1H), 5.03 (dd, *J* = 17.4, 1.7 Hz, 1H), 5.00 (dd, *J* = 10.4, 1.4 Hz, 1H), 4.30 (dt, *J* = 11.8, 5.0 Hz, 1H), 4.13 (dt, *J* = 12.2, 4.9 Hz, 1H), 3.83 (t, *J* = 6.8 Hz, 1H), 2.89 (t, *J* = 5.0 Hz, 2H), 2.38 (q, *J* = 7.5 Hz, 2H), 2.07-2.01 (m, 2H), 2.00-1.86 (m, 2H), 1.55 (s, 9H), 1.15 (t, *J* = 7.5 Hz, 3H). **^{13}C{^{1}H} NMR** (126 MHz, $CDCl_3$): δ 174.5, 157.7, 151.8, 151.7, 137.2, 129.7, 115.7 (2C unresolved), 112.0, 110.1, 83.5, 62.5, 62.2, 45.8, 30.2, 27.7 (2C unresolved), 27.4, 9.0. **HRMS (ESI)** m/z calculated for $C_{21}H_{31}NO_6Na$ $[M+Na]^+$: 416.2049; found 416.2032. **R_f** = 0.3 (hexanes/ethyl acetate = 3:1).

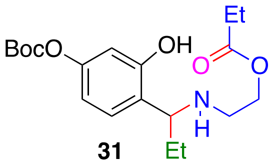

31

2-((1-(4-((*Tert*-butoxycarbonyl)oxy)-2-hydroxyphenyl)propyl)amino)ethyl propionate (31). Prepared according to the general procedure D. Attempted purification was performed by quick column chromatography (eluent: hexanes/ethyl acetate = 2:1) to yield an yellow oil (23.7 mg, 64% isolated yield). Numerous impurities were found inseparable from the desired product. Chromatography at a slower rate leads to decomposition of product. **^{1}H NMR** (600 MHz, $CDCl_3$): δ 6.90 (d, *J* = 8.4 Hz, 1H) 6.66 (d, *J* = 1.8 Hz, 1H) 6.60 (dd, *J* = 8.4, 2.4 Hz, 1H) 4.30 (dt, *J* = 16.8, 4.8 Hz, 1H), 4.12 (dt, *J* = 12, 4.8 Hz, 1H), 3.69 (t, *J* = 7.2 Hz, 1H) 2.37 (q, *J* = 7.8 Hz, 2H) 1.89 – 1.77 (m, 2H) 1.54 (s, 3H), 1.15 (t, *J* = 7.2 Hz, 3H), 0.88 (t, *J* = 7.2 Hz, 3H). **^{13}C{^{1}H} NMR** (126 MHz, $CDCl_3$): δ 174.5, 157.7, 151.8, 151.6, 129.7, 128.9, 111.9, 110.0, 83.4, 64.6, 62.2, 45.8, 27.7, 27.6, 27.4, 10.7, 9.0). **HRMS (ESI)** m/z calculated for $C_{19}H_{29}NO_6Na$ $[M+Na]^+$: 390.1893; found 390.1890. **R_f** = 0.2 (hexanes/ethyl acetate = 3:1).

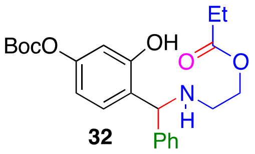

32

2-(((4-((*Tert*-butoxycarbonyl)oxy)-2-hydroxyphenyl)(phenyl)methyl)amino)ethyl propionate (32). Prepared according to the general procedure D (purified by column chromatography, eluent: hexanes/ethyl acetate = 8:1→3:1). Yellow oil (29.1 mg, 70% isolated yield). **^{1}H NMR** (600 MHz, $CDCl_3$): δ 7.39-7.27 (m, 5H), 6.79 (d, *J* = 8.3 Hz, 1H), 6.70 (d, *J* = 1.7 Hz, 1H), 6.55 (dd, *J* = 8.3, 2.1 Hz, 1H), 4.93 (s, 1H), 4.30 (ddd, *J* = 11.7, 5.9, 4.0 Hz, 1H), 4.17 (ddd, *J* = 11.5, 7.0, 4.2 Hz, 1H), 3.01-2.91 (m, 2H), 2.36 (q, *J* = 7.4 Hz, 2H), 1.54 (s, 9H), 1.15 (t, *J* = 7.5 Hz, 3H). **^{13}C{^{1}H} NMR** (126 MHz, $CDCl_3$): δ 174.3, 158.5, 151.8, 151.6, 129.6, 129.3, 129.1, 128.5, 128.1, 127.4,

122.0, 112.0, 110.2, 83.4, 66.8, 62.6, 46.3, 27.7, 9.0. **HRMS (ESI)** m/z calculated for $C_{23}H_{30}NO_6$ $[M+H]^+$: 416.2073; found 416.2068. $\mathbf{R_f}$ = 0.4 (hexanes/ethyl acetate = 3:1).

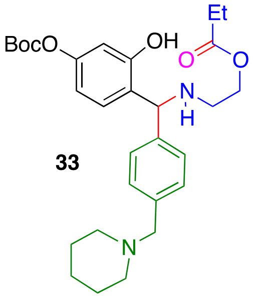

2-(((4-((*Tert*-butoxycarbonyl)oxy)-2-hydroxyphenyl)(4-(piperidin-1-ylmethyl)phenyl)methyl)amino)ethyl propionate (33). Prepared according to the general procedure D (purified by column chromatography, eluent: hexanes/ethyl acetate = 1:1→100% ethyl acetate). Yellow oil (34.9 mg, 68% isolated yield). **^{1}H NMR** (600 MHz, $CDCl_3$): δ 7.35 (d, *J* = 7.7 Hz, 2H), 7.29 (d, *J* = 8.0 Hz, 2H), 6.81 (d, *J* = 8.3 Hz, 1H), 6.68 (d, *J* = 2.4 Hz, 1H), 6.55 (dd, *J* = 8.3, 2.4 Hz, 1H), 4.91 (s, 1H), 4.30 (ddd, *J* = 11.7, 6.2, 3.7 Hz, 1H), 4.16 (ddd, *J* = 11.5, 7.1, 4.0 Hz, 1H), 3.55 (br s, 2H), 3.01-2.88 (m, 2H), 2.46 (br s, 4H), 2.36 (q, *J* = 7.7 Hz, 2H), 1.64 (br s, 4H), 1.54 (s, 9H), 1.46 (br s, 2H), 1.14 (t, *J* = 7.7 Hz, 3H). **^{13}C{^{1}H} NMR** (126 MHz, $CDCl_3$): δ 174.3, 158.4, 151.8, 151.6, 130.5, 129.6, 127.4, 121.9, 112.1, 110.3, 83.5, 66.7, 62.7, 54.0, 46.4, 31.7, 29.7, 27.7 (2C unresolved), 27.4, 24.9, 23.7, 9.0. **HRMS (ESI)** m/z calculated for $C_{29}H_{41}N_2O_6$ $[M+H]^+$: 513.2964; found 513.2955. $\mathbf{R_f}$ = 0.1 (hexanes/ethyl acetate = 1:1).

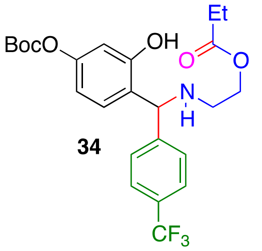

2-(((4-((*Tert*-butoxycarbonyl)oxy)-2-hydroxyphenyl)(4-(trifluoromethyl)phenyl)methyl)amino)ethyl propionate (34). Prepared according to the general procedure D. Attempted purification was performed by quick column chromatography (eluent: hexanes/ethyl acetate = 3:1) to yield an yellow oil (26.3 mg, 55% isolated yield). Numerous impurities were found inseparable from the desired product. Chromatography at a slower rate leads to decomposition of product. **^{1}H NMR** (600 MHz, $CDCl_3$): δ 7.61 (d, *J* = 7.8 Hz, 2H), 7.49 (d, *J* = 7.8 Hz, 2H), 6.80 (d, *J* = 8.4 Hz, 1H), 6.70 (d, *J* = 2.4 Hz, 1H), 6.58 (dd, *J* = 6.0, 2.4 Hz, 1H), 4.99 (s, 1H) 4.32 (dt, *J* = 12, 5.4 Hz, 1H), 4.18 (dt, *J* = 12.6, 4.8 Hz, 1H), 2.97 (t, *J* = 6.0 Hz, 2H), 2.37 (q, *J* = 7.8 Hz, 2H), 1.54 (s, 9H), 1.15 (t, *J* = 7.8 Hz, 3H). **^{13}C{^{1}H} NMR** (126 MHz, $CDCl_3$): δ 174.3, 158.3 151.9, 151.7, 144.8, 130.5, 130.4 (q, $^2J_{C-F}$ = 32.7 Hz), 129.4, 127.7, 126.1 (q, $^3J_{C-F}$ = 15 Hz), 123.9 (q, $^1J_{C-F}$ = 272.5 Hz), 112.4, 110.6, 83.6, 66.5, 62.5, 46.4, 27.7, 27.4, 9.0. **IR** (neat, cm^{-1}): 3306, 2988, 2870, 1758, 1618, 1457.

HRMS (ESI) m/z calculated for $C_{24}H_{29}F_3NO_6$ $[M+H]^+$: 484.1947; found 484.1945. $\mathbf{R_f}$ = 0.4 (hexanes/ethyl acetate = 3:1).

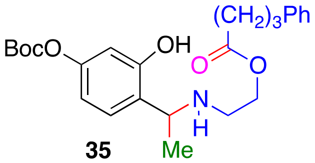

35

2-((1-(4-((*Tert*-butoxycarbonyl)oxy)-2-hydroxyphenyl)ethyl)amino)ethyl 4-phenylbutanoate (35). Prepared according to the general procedure D (purified by column chromatography, eluent: hexanes/ethyl acetate = 8:1→3:1). Yellow oil (28.0 mg, 63% isolated yield). ^{1}**H NMR** (600 MHz, $CDCl_3$): δ 7.31-7.21 (m, 2H), 7.22-7.17 (m, 3H), 6.91 (d, *J* = 8.3 Hz, 1H), 6.61-6.57 (m, 2H), 4.25 (ddd, *J* = 11.4, 6.3, 3.5 Hz, 1H), 4.09 (ddd, *J* = 11.5, 7.3, 3.8 Hz, 1H), 3.94 (q, *J* = 6.6 Hz, 1H), 2.91-2.79 (m, 2H), 2.67 (t, *J* = 7.5 Hz, 2H), 2.36 (t, *J* = 7.7 Hz, 2H), 1.98 (quin, *J* = 7.5 Hz, 2H), 1.55 (s, 9H), 1.44 (d, *J* = 6.6 Hz, 3H). ^{13}**C{^{1}H} NMR** (126 MHz, $CDCl_3$): δ 173.3, 158.0, 151.9, 151.3, 141.2, 128.47, 128.45, 128.4, 126.0, 123.7, 111.9, 110.0, 83.4, 62.9, 58.3, 45.8, 35.1, 33.4, 27.7, 26.3, 22.3. **HRMS (ESI)** m/z calculated for $C_{25}H_{34}NO_6$ $[M+H]^+$: 444.2386; found 444.2397 $\mathbf{R_f}$ = 0.4 (hexanes/ethyl acetate = 3:1).

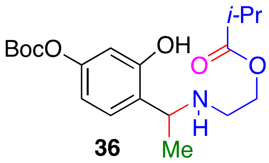

36

2-((1-(4-((*Tert*-butoxycarbonyl)oxy)-2-hydroxyphenyl)ethyl)amino)ethyl isobutyrate (36). Prepared according to the general procedure D (purified by column chromatography, eluent: hexanes/ethyl acetate = 5:1→1:1). Yellow oil (16.1 mg, 44% isolated yield). ^{1}**H NMR** (600 MHz, $CDCl_3$): δ 6.92 (d, *J* = 8.3 Hz, 1H), 6.63 (d, *J* = 2.4 Hz, 1H), 6.59 (dd, *J* = 8.3, 2.4 Hz, 1H), 4.26 (ddd, *J* = 11.6, 6.3, 3.7 Hz, 1H), 4.11 (ddd, *J* = 11.4, 7.0, 4.2 Hz, 1H), 3.97 (q, *J* = 6.8 Hz, 1H), 2.94-2.80 (m, 2H), 2.59 (sept, *J* = 7.0 Hz, 1H), 1.54 (s, 9H), 1.45 (d, *J* = 6.6 Hz, 3H), 1.18 (d, *J* = 8.0 Hz, 6H). ^{13}**C{^{1}H} NMR** (126 MHz, $CDCl_3$): δ 177.0, 157.9, 151.9, 151.4, 128.5 (2C unresolved), 111.9, 110.0, 83.4, 62.7, 58.1, 45.8, 33.9, 27.7, 22.1, 19.0. **HRMS (ESI)** m/z calculated for $C_{19}H_{30}NO_6$ $[M+H]^+$: 368.2073; found 368.2068. $\mathbf{R_f}$ = 0.3 (hexanes/ethyl acetate = 3:1).

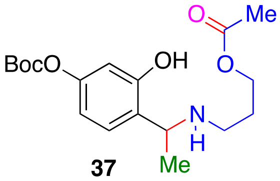

37

3-((1-(4-((*Tert*-butoxycarbonyl)oxy)-2-hydroxyphenyl)ethyl)amino)propyl acetate (37). Prepared according to the general procedure D (purified by column chromatography, eluent:

hexanes/ethyl acetate = 5:1→1:1). Yellow oil (24.7 mg, 70% isolated yield). **^{1}H NMR** (600 MHz, $CDCl_3$): δ 6.90 (d, *J* = 8.3 Hz, 1H), 6.59 (d, *J* = 2.1 Hz, 1H), 6.57 (dd, *J* = 8.4, 2.4 Hz, 1H), 4.16 (dt, *J* = 11.7, 6.0 Hz, 1H), 4.07 (dt, *J* = 11.7, 6.0 Hz, 1H), 3.90 (q, *J* = 6.6 Hz, 1H), 2.74-2.62 (m, 2H), 2.03 (s, 3H), 1.89-1.77 (m, 2H), 1.54 (s, 9H), 1.42 (d, *J* = 7.0 Hz, 3H). **^{13}C{^{1}H} NMR** (126 MHz, $CDCl_3$): δ 171.1, 158.2, 151.9, 151.2, 128.3, 124.0, 111.7, 109.9, 83.3, 62.2, 58.7, 44.2, 28.7, 27.7, 22.5, 20.9. **IR** (neat, cm^{-1}): 3318, 2980, 2869, 1739, 1501, 1460. **HRMS (ESI)** m/z calculated for $C_{18}H_{28}NO_6$ $[M+H]^+$: 354.1917; found 354.1913. **R_f** = 0.2 (hexanes/ethyl acetate = 3:1).

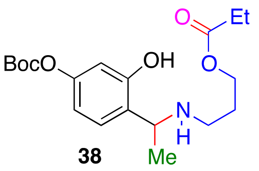

38

3-((1-(4-((*Tert*-butoxycarbonyl)oxy)-2-hydroxyphenyl)ethyl)amino)propyl propionate (38). Prepared according to the general procedure D (purified by column chromatography, eluent: hexanes/ethyl acetate = 5:1→1:1). Yellow oil (28.6 mg, 78% isolated yield). **^{1}H NMR** (600 MHz, $CDCl_3$): δ 6.90 (d, *J* = 8.3 Hz, 1H), 6.61-6.52 (m, 2H), 4.17 (dt, *J* = 11.7, 6.0 Hz, 1H), 4.08 (dt, *J* = 11.7, 6.1 Hz, 1H), 3.90 (q, *J* = 6.6 Hz, 1H), 2.68 (t, *J* = 6.8 Hz, 2H), 2.30 (q, *J* = 7.7 Hz, 2H), 1.89-1.77 (m, 2H), 1.54 (s, 9H), 1.42 (d, *J* = 7.0 Hz, 3H), 1.12 (t, *J* = 7.5 Hz, 3H). **^{13}C{^{1}H} NMR** (126 MHz, $CDCl_3$): δ 174.5, 158.2, 151.9, 151.2, 128.3, 124.0, 111.7, 109.8, 83.3, 62.1, 58.7, 44.2, 28.7, 27.7, 27.5, 22.5, 9.1. **HRMS (ESI)** m/z calculated for $C_{19}H_{30}NO_6$ $[M+H]^+$: 368.2073; found 368.2073. **R_f** = 0.2 (hexanes/ethyl acetate = 3:1).

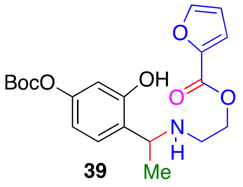

39

2-((1-(4-((*Tert*-butoxycarbonyl)oxy)-2-hydroxyphenyl)ethyl)amino)ethyl furan-2-carboxylate (39). Prepared according to the general procedure D (purified by column chromatography, eluent: hexanes/ethyl acetate = 5:1→1:1). Yellow oil (11.4 mg, 29% isolated yield). **^{1}H NMR** (600 MHz, $CDCl_3$): δ 7.60 (d, *J* = 0.6 Hz, 1H), 6.93 (d, *J* = 8.3 Hz, 1H), 6.63 (d, *J* = 2.4 Hz, 1H), 6.60 (dd, *J* = 8.2, 2.3 Hz, 1H), 6.53 (dd, *J* = 3.5, 1.7 Hz, 1H), 4.48 (ddd, *J* = 11.5, 6.6, 3.5 Hz, 1H), 4.34 (ddd, *J* = 11.3, 7.1, 3.8 Hz, 1H), 4.00 (q, *J* = 6.7 Hz, 1H), 3.01 (ddd, *J* = 13.5, 6.9, 3.3 Hz, 1H), 2.95 (ddd, *J*=13.3, 6.5, 3.8 Hz, 1H), 1.55 (s, 9H), 1.46 (d, *J* = 7.0 Hz, 3H). **^{13}C{^{1}H} NMR** (126 MHz, $CDCl_3$): δ 158.5, 158.0, 151.9, 151.3, 146.6, 144.2, 128.5, 123.6, 118.6, 112.00, 111.97, 110.1, 83.4, 63.4, 58.2, 49.2, 45.8, 27.7. **HRMS (ESI)** m/z calculated for $C_{20}H_{26}NO_7$ $[M+H]^+$: 392.1709; found 392.1708. **R_f** = 0.2 (hexanes/ethyl acetate = 3:1).

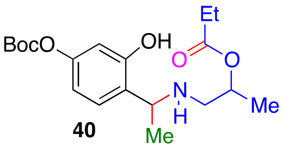

40

1-((1-(4-((*Tert*-butoxycarbonyl)oxy)-2-hydroxyphenyl)ethyl)amino)propan-2-yl propionate (40). Prepared according to the general procedure D (purified by column chromatography, eluent: hexanes/ethyl acetate = 5:1→1:1). Yellow oil (19.1 mg, 52% isolated yield, d.r. = 1.9:1, inseparable diastereomers). **HRMS (ESI)** m/z calculated for $C_{19}H_{30}NO_6$ $[M+H]^+$: 368.2073; found 368.2063. $\mathbf{R_f}$ = 0.2 (hexanes/ethyl acetate = 3:1).

Major Diastereomer:

^{1}H NMR (600 MHz, $CDCl_3$): δ 6.91 (d, *J* = 8.0 Hz, 1H), 6.61-6.57 (m, 2H), 5.02-4.94 (m, 1H), 3.90-3.87 (m, 1H), 2.77-2.66 (m, 2H), 2.42-2.29 (m, 2H), 1.54 (s, 9H), 1.43 (d, *J* = 6.6 Hz, 3H), 1.21 (d, *J* = 6.6 Hz, 3H), 1.14 (t, *J* = 7.7 Hz, 3H). **^{13}C{^{1}H} NMR** (126 MHz, $CDCl_3$): δ 174.0, 158.0, 151.9, 151.3, 128.4, 123.9, 111.8, 110.0, 83.4, 70.1, 68.4, 58.7, 52.0, 27.7, 22.2, 18.1, 9.1.

Minor Diastereomer:

^{1}H NMR (600 MHz, $CDCl_3$): δ 6.91 (d, *J* = 8.0 Hz, 1H), 6.61-6.57 (m, 2H), 5.14-5.07 (m, 1 H), 4.94 - 5.02 (m, 1H), 3.94-3.90 (m, 1H), 2.78-2.68 (m, 2H), 2.42-2.29 (m, 2H), 1.54 (s, 9H), 1.42 (d, *J* = 6.6 Hz, 3H), 1.21 (d, *J* = 6.3 Hz, 3H), 1.16 (q, *J* = 7.7 Hz, 3H). **^{13}C{^{1}H} NMR** (126 MHz, $CDCl_3$): δ 174.3, 158.1, 151.9, 151.4, 128.4, 123.7, 111.8, 110.0, 83.5, 71.3, 70.1, 58.1, 51.8, 27.8, 22.4, 17.9, 9.1.

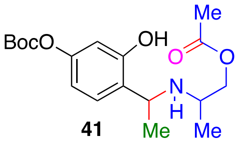

41

2-((1-(4-((*Tert*-butoxycarbonyl)oxy)-2-hydroxyphenyl)ethyl)amino)propyl acetate (41). Prepared according to the general procedure D. Attempted purification was performed by quick column chromatography (eluent: hexanes/ethyl acetate = 2:1) to yield an yellow oil (29.4 mg, 42% isolated yield, d.r. = 1.4:1, inseparable diastereomers). Numerous impurities were found inseparable from the desired product. Chromatography at a slower rate leads to decomposition of product. **HRMS (ESI)** m/z calculated for $C_{18}H_{28}NO_6$ $[M+H]^+$: 354.1917; found 354.1906. $\mathbf{R_f}$ = 0.2 (hexanes/ethyl acetate = 3:1).

Major Diastereomer:

^{1}H NMR (600 MHz, $CDCl_3$): δ 6.93 (d, *J* = 8.4 Hz), 6.65 (s, 1H), 6.61 - 6.58 (m, 1H), 4.23 (dd, *J* = 11.4, 3.6 Hz, 1H), 4.14 – 4.10 (m, 1H), 3.95 (dd, *J* = 11.4, 4.8 Hz, 1H), 3.04 (sex, *J* = 6 Hz, 1H), 2.10 (s, 3H), 1.54 (s, 9H), 1.44 (d, *J* = 6.6 Hz, 3H), 1.14 (d, *J* = 6.6 Hz, 3H). **^{13}C{^{1}H} NMR** (126 MHz, $CDCl_3$): δ 170.9, 157.9, 151.8, 151.4, 128.3, 123.8, 112.0, 110.1, 83.4, 65.4, 55.7, 49.7, 27.7, 22.0, 20.8, 17.6.

Minor Diastereomer:

^{1}H NMR (600 MHz, $CDCl_3$): δ 6.94 (d, *J* = 7.8 Hz), 6.70 (s, 1H), 6.61-6.58 (m, 1H), 4.14 – 4.10 (m, 1H), 4.06 – 4.03 (m, 2H), 2.98 (sex, *J* = 6 Hz, 1H), 2.09 (s, 3H), 1.54 (s, 9H), 1.50 (d, *J* = 6.6 Hz, 3H), 1.19 (d, *J* = 6.6 Hz, 3H). **$^{13}C\{^1H\}$ NMR** (126 MHz, $CDCl_3$): δ 171.0, 157.6, 151.8, 151.6, 128.6, 122.8, 112.2, 110.2, 83.4, 66.9, 55.5, 50.0, 29.7, 21.8, 20.8, 15.4.

*1.0 mmol scale synthesis of adduct **25***

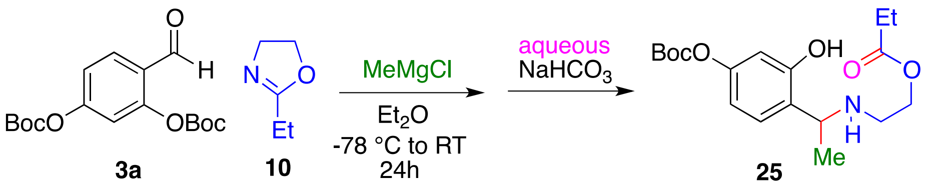

To a flame-dried Schlenk flask was charged with aldehyde **3a** solution (1.0 mL, 1.0 mmol, 1.0 M in toluene) and dry Et_2O (10 mL). Methylmagnesium chloride (0.40 mL, 1.05 mmol, 2.6M solution in THF, 1.05 equiv) was added to the mixture at -78 ºC. The mixture was allowed to stir at the same temperature for 10 minutes before addition of a solution of **10** (2.0 mL, 2.0 mmol, 1.0 M in toluene, 2 equiv.). The mixture was allowed to warm to room temperature over 16 hours. The solution was quenched with saturated $NaHCO_3$ solution (10 mL), extracted with Et_2O (3 × 10 mL). Organic layers were combined, washed with brine (10 mL), dried over anhydrous Na_2SO_4, and concentrated. The crude product was purified by column chromatography (SiO_2, eluent: hexanes/ethyl acetate = 10:1→1:1) to afford pure adduct **25** as yellow oil (240 mg, 68% isolated yield). ^{1}H NMR of the product is identical to that of 0.1 mmol scale reaction.

4. Transformations of Adduct 25

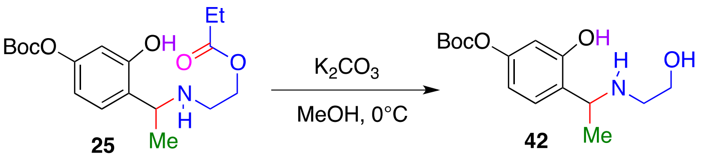

***Tert*-butyl (3-hydroxy-4-vinylphenyl) carbonate (42)**. A solution of **25** (17.7 mg, 0.05 mmol) in methanol (1.0 mL) was cooled to 0 ºC. Potassium carbonate (20.8 mg, 0.15 mmol, 3.0 equiv) was then added. The suspension was stirred at 0 ºC for 20 minutes, after which the suspension was filtered. The filtrate collected was concentrated and purified by column chromatography (SiO_2, eluent: hexanes/ethyl acetate = 1:1→1:3) to afford pure product as brown oil (10.4 mg, 70% isolated yield). **^{1}H NMR** (500 MHz, $CDCl_3$): δ 6.92 (d, J = 8.3 Hz, 1H), 6.63 (d, J = 2.3 Hz, 1H), 6.58 (dd, J = 8.2, 2.5 Hz, 1H), 4.31 (q, J = 6.7 Hz, 1H), 3.62 (br s, 2H), 2.88 (br s, 2H), 1.54 (s, 9H), 1.46 (d, J = 6.7 Hz, 3H). **^{13}C{^{1}H} NMR** (126 MHz, $CDCl_3$): δ 158.5, 151.9, 151.2, 127.5, 125.7, 111.7, 110.3, 83.3, 63.7, 51.3, 43.5, 27.7, 23.8. **HRMS (ESI)** m/z calculated for $C_{15}H_{24}NO_5$ $[M+H]^+$: 298.3548; found 298.3543. **R_f** = 0.2 (hexanes/ethyl acetate = 1:1).

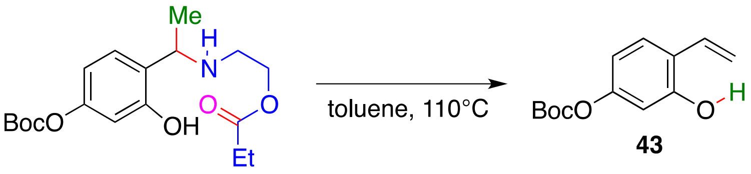

***Tert*-butyl (3-hydroxy-4-vinylphenyl) carbonate (43)**. A solution of **25** (70.6 mg, 0.2 mmol) in toluene (2.0 mL) was refluxed for 24 hours, then concentrated *in vacuo*. The residue was purified by column chromatography (SiO_2, eluent: hexanes/ethyl acetate = 5:1→3:1) to afford pure **43** as white solid (35.4 mg, 75% isolated yield). **^{1}H NMR** (600 MHz, $CDCl_3$): δ 7.35 (d, J = 8.7 Hz, 1H), 6.86 (dd, J = 17.7, 11.1 Hz, 1H), 6.73 (dd, J = 8.5, 2.3 Hz, 1H), 6.65 (d, J = 2.4 Hz, 1H), 5.68 (dd, J = 17.6, 1.2 Hz, 1H), 5.33 (dd, J = 11.3, 1.2 Hz, 1H), 5.31 (br s, 1H), 1.56 (s, 9H). **^{13}C{^{1}H} NMR** (126 MHz, $CDCl_3$): δ 153.4, 151.7, 151.0, 130.8, 127.9, 122.7, 115.9, 113.7, 109.0, 83.8, 27.7. **HRMS (ESI)** Dimerized in ESI condition, m/z calculated for dimer $C_{26}H_{32}O_8Na$ $[2M+Na]^+$: 495.1995; found 495.2018. **R_f** = 0.5 (hexanes/ethyl acetate = 3:1).

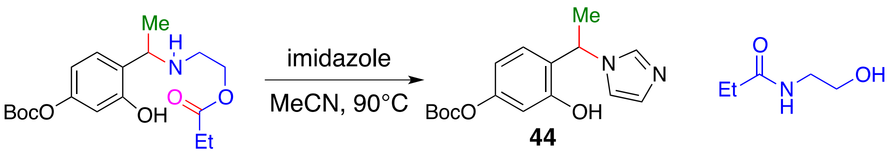

4-(1-(1H-imidazol-1-yl)ethyl)-3-hydroxyphenyl tert-butyl carbonate (44).

A solution of **25** (15.0 mg, 0.042 mmol) and imidazole (3.2 mg, 0.047 mmol) in acetonitrile (1.0 mL) was heated to reflux for 72 hours. The solution was then concentrated *in vacuo*. NMR analysis of this residue showed the presence of *N*-(2-hydroxyethyl)propionamide by comparing to its reported

spectrum[18]. The residue was purified by column chromatography (SiO_2, eluent: 10% MeOH in DCM) to afford **44** as a yellow oil (7.0 mg, 55% isolated yield). **^{1}H NMR** (600 MHz, $CDCl_3$): δ 7.69 (s, 1H), 7.14 (d, *J* = 8.4 Hz, 1H), 7.00 (s, 1H), 6.95 (s, 1H), 6.76 (s, 1H), 6.67 (dd, *J* = 8.4, 1.8 Hz, 1H), 5.72 (q, *J* = 6.6 Hz), 1.84 (d, *J* = 7.2 Hz, 3H), 1.53 (s, 9H). **^{13}C{^{1}H} NMR** (126 MHz, $CDCl_3$): δ 156.4, 152.2, 151.8, 151.0, 138.9, 127.1 (2C unresolved), 122.8, 112.4, 110.0, 83.8, 53.1, 27.7, 19.2. **HRMS (ESI)** m/z calculated for $C_{16}H_{21}N_2O_4$ $[M+H]^+$: 305.1501; found 305.1501. **R_f** = 0.5 (10% MeOH in DCM).

[18] Mahmoud, A. M.; Morrow, J. P.; Pizzi, D.; Nanayakkara, S.; Davis, T. P.; Saito, K.; Kempe, K. *Macromolecules.* **2020**, *53* (2), 693-701.

5. Experiments with *o*-QM Precursors in Scheme 4

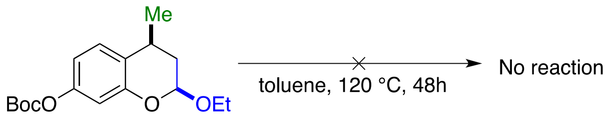

45a: >20:1 cis:trans

Heating 45a at 120ºC: No reaction

A sealed tube was charged with a solution of **45a** (15.4 mg, 0.05 mmol) in toluene (1 mL). The solution was heated at 120 ºC for 48 hours. Upon cooling to room temperature, the solution was concentrated *in vacuo*. NMR analysis of the residue showed it contained pure **45a** only, with no stereochemical corruption.

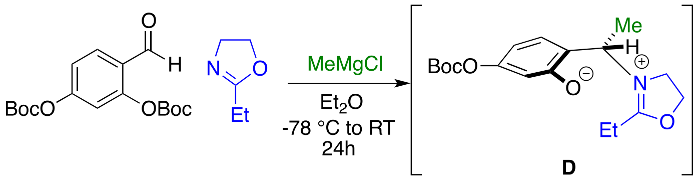

D

***5-((Tert-butoxycarbonyl)oxy)-2-(1-(2-ethyl-4,5-dihydrooxazol-3-ium-3-yl)ethyl)phenolate magnesium bromide* (D).**

To a flame-dried Schlenk tube was charged with aldehyde **3a** solution (0.1 mL, 0.1 mmol, 1.0 M in toluene) and dry Et_2O (1 mL). Methylmagnesium chloride (0.04 mL, 0,105 mmol, 2.6M solution in THF, 1.05 equiv) was added to the mixture at -78 ºC. The mixture was allowed to stir at the same temperature for 10 minutes before addition of dihydrooxazole **10** solution (0.2 mL, 0.2 mmol, 1.0 M in toluene, 2 equiv.). The mixture was allowed to warm to room temperature over 16 hours. Et_2O and dihydrooxazole **10** were removed in vacuo. The residue was taken up in dry CD_2Cl_2 and transferred into a NMR tube under argon atmosphere.

Characteristic shifts: **^{1}H NMR** (600 MHz, CD_2Cl_2): δ 4.30 (3H, C***H*$_2$**O + Ar-C***H***-CH_3), 3.86 (2H, C***H*$_2$**N), 2.44 (2H, C***H*$_2$**-CH_3).

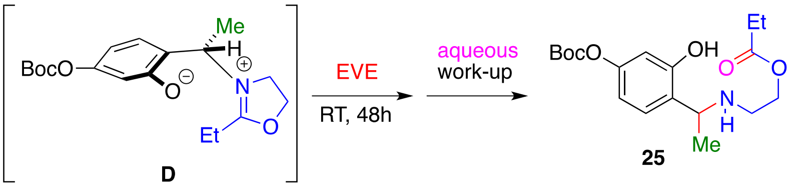

25

Oxazolium Intermediate D with EVE at room temperature: No reaction

To a Schlenk tube containing crude solution of **D** in Et_2O was added ethyl vinyl ether (0.5 mL). The mixture was allowed to stir for another 48 hours. The solution was then quenched with saturated $NaHCO_3$ solution (1 mL), extracted with Et_2O (3 × 1mL). Organic layers were combined, washed with brine (1 mL), dried over anhydrous Na_2SO_4, and concentrated. NMR analysis of this crude product showed the presence of **25**, but no indication of **45a** or **45b**.

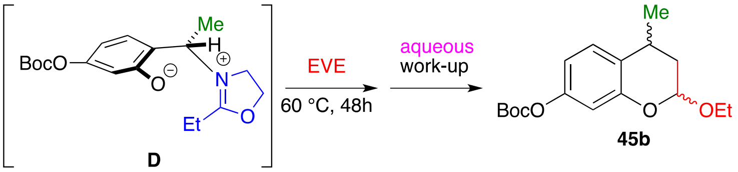

Oxazolium Intermediate D with EVE at 60 ºC: *Tert-butyl (2-ethoxy-4-methylchroman-7-yl) carbonate* (45b).

A sealed tube under argon atmosphere was charged with crude solution of oxazolium ion **D**. To this solution was added 0.5 mL of ethyl vinyl ether at room temperature. The mixture was heated to 60 ºC for 48 hours. The solution was quenched with saturated $NaHCO_3$ solution (1 mL), extracted with Et_2O (3 × 1mL). Organic layers were combined, washed with brine (1 mL), dried over anhydrous Na_2SO_4, and concentrated. The crude product was purified by column chromatography (SiO_2, eluent: hexanes/ethyl acetate = 5:1→1:1) to afford pure products. Yellow oil (19.8 mg, 70% isolated yield, d.r. = 1.6:1, inseparable isomers). $\mathbf{R_f}$ = 0.6 (hexanes/ethyl acetate = 9:1).

Major, cis Isomer:

^{1}H NMR (600 MHz, $CDCl_3$): δ 7.14 (d, *J* = 8.3 Hz, 1H), 6.71 (dd, *J* = 8.3, 2.4 Hz, 1H), 6.67 (d, *J* = 2.4 Hz, 1H), 5.18 (dd, *J* = 7.1, 2.6 Hz, 1H), 4.02-3.94 (m, 1H), 3.65-3.57 (m, 1H), 2.99-2.93 (m, 1H), 2.18-2.11 (m, 1H), 1.79-1.68 (m, 1H), 1.55 (s, 9H), 1.36 (d, *J* = 7.0 Hz, 3H), 1.25 (t, *J* = 7.0 Hz, 3H). This *cis* isomer has been reported. Our characterization data match with prior literature data.[19]

Minor, trans Isomer:

^{1}H NMR (600 MHz, $CDCl_3$): δ 7.01 (d, *J* = 9.0 Hz, 1H), 6.41 (dd, *J* = 8.3, 2.8 Hz, 1H), 6.33 (d, *J* = 2.4 Hz, 1H), 5.16 (dd, *J* = 7.1, 2.6 Hz, 1H), 4.02-3.94 (m, 1H), 3.65-3.57 (m, 1H), 2.94-2.87 (m, 1H), 2.18-2.11 (m, 1H), 1.79-1.68 (m, 1H), 1.55 (s, 9H), 1.34 (d, *J* = 7.0 Hz, 3H), 1.25 (t, *J* = 7.0 Hz, 3H).

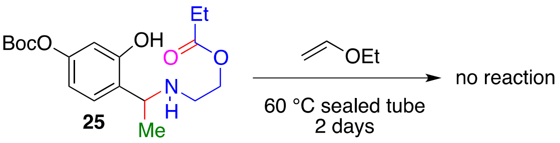

Adduct 25 with EVE at 60 ºC: No reaction

A sealed tube was charged with a solution of **25** (35.3 mg, 0.1 mmol) in EVE (1 mL). The solution was heated at 60 ºC for 48 hours. Upon cooling to room temperature, the solution was concentrated *in vacuo*. NMR analysis of this crude product showed it contained pure **25** only, and no indication of **45a** or **45b**.

[19] Van De Water, R. W.; Magdziak, D. J.; Chau, J. N.; Pettus, T. R. R. *J. Am. Chem. Soc.* **2000**, *122* (27), 6502–6503.

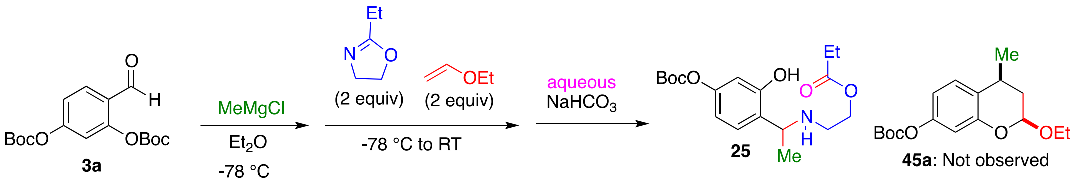

Competition between 10 and EVE as nucleophiles towards *o*-QM

To a flame-dried Schlenk tube was charged with *o*-OBoc aldehyde solution (0.1 mL, 0.1 mmol, 1.0 M in toluene) and dry Et_2O (1 mL). Grignard reagent (1.05 equiv) was added to the mixture at -78 ºC. The mixture was allowed to stir at the same temperature for 10 minutes before addition of a solution of **10** (0.2 mL, 0.2 mmol, 1.0 M in toluene, 2 equiv.) and EVE (14.4 mg, 20 µL, 0.2 mmol, 2 equiv). The mixture was allowed to warm to room temperature over 16 hours. The solution was quenched with saturated $NaHCO_3$ solution (1 mL), extracted with Et_2O (3 × 1mL). Organic layers were combined, washed with brine (1 mL), dried over anhydrous Na_2SO_4, and concentrated. CH_2Br_2 (7 µL, 0.1 mmol) was added to the crude mixture as internal standard. NMR analysis of this mixture showed **25** was formed in 72% yield (by 600 MHz ^{1}H NMR). **45a** was not observed in the NMR of the crude mixture.

6. Adducts in Scheme 5

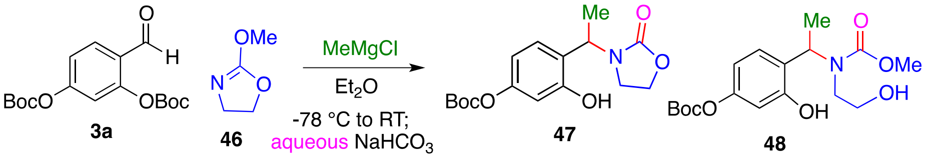

To a flame-dried Schlenk tube was charged with aldehyde **3a** solution (0.1 mL, 0.1 mmol, 1.0 M in toluene) and dry Et_2O (1 mL). Methylmagnesium chloride (0.04 mL, 0,105 mmol, 2.6M solution in THF, 1.05 equiv) was added to the mixture at -78 ºC. The mixture was allowed to stir at the same temperature for 10 minutes before addition of a solution of **46** (0.2 mL, 0.2 mmol, 1.0 M in toluene, 2 equiv.). The mixture was allowed to warm to room temperature over 16 hours. The solution was quenched with saturated $NaHCO_3$ solution (1 mL), extracted with Et_2O (3 × 1mL). Organic layers were combined, washed with brine (1 mL), dried over anhydrous Na_2SO_4, and concentrated. The crude product was purified by column chromatography (SiO_2, eluent: hexanes/ethyl acetate = 10:1→1:1).

***Tert-butyl (3-hydroxy-4-(1-(2-oxooxazolidin-3-yl)ethyl)phenyl) carbonate* (47)**.

White solid (17.5 mg, 54% isolated yield). **^{1}H NMR** (600 MHz, $CDCl_3$): δ 8.33 (br s, 1H), 7.18 (d, *J* = 8.3 Hz, 1H), 6.79 (d, *J* = 2.4 Hz, 1H), 6.70 (dd, *J* = 8.3, 2.4 Hz, 1H), 5.30 (q, *J* = 7.3 Hz, 1H), 4.39-4.25 (m, 2H), 3.65-3.56 (m, 1H), 3.38-3.28 (m, 1H), 1.60 (d, *J* = 7.3 Hz, 3H), 1.54 (s, 9H). **^{13}C{^{1}H} NMR** (126 MHz, $CDCl_3$): δ 160.0, 156.1, 151.8, 151.7, 126.7, 122.8, 112.7, 111.3, 83.6, 62.9, 45.5, 39.9, 27.6, 16.4. **IR** (neat, cm^{-1}): 3162, 2980, 2870, 1755, 1715, 1605, 1428. **HRMS (ESI)** m/z calculated for $C_{16}H_{21}NO_6Na$ $[M+Na]^+$: 346.1266; found 346.1274. **R_f** = 0.1 (hexanes/ethyl acetate = 3:1).

***Methyl (1-(4-((tert-butoxycarbonyl)oxy)-2-hydroxyphenyl)ethyl)(2-hydroxyethyl)carbamate* (48)**.

White solid (6.7 mg, 19% isolated yield). **^{1}H NMR** (600 MHz, $CDCl_3$): δ 7.22 (d, *J* = 8.3 Hz, 1H), 6.75 (d, *J* = 2.3 Hz, 1H), 6.72 (dd, *J* = 8.3, 2.3 Hz, 1H), 5.52 (q, *J* = 7.2 Hz, 1H), 3.79 (s, 3H), 3.44-3.29 (m, 4H), 1.59-1.57 (m, 3 H), 1.56-1.53 (m, 12 H). **^{13}C{^{1}H} NMR** (126 MHz, $CDCl_3$): δ 156.2, 152.0, 151.6, 126.8, 123.3, 112.7, 110.8, 83.7, 53.8, 48.3, 44.0, 40.8, 27.7, 17.4. **HRMS (ESI)** m/z calculated for $C_{17}H_{26}NO_7$ $[M+H]^+$: 356.1709; found 356.1714. **R_f** = 0.3 (hexanes/ethyl acetate = 3:1).

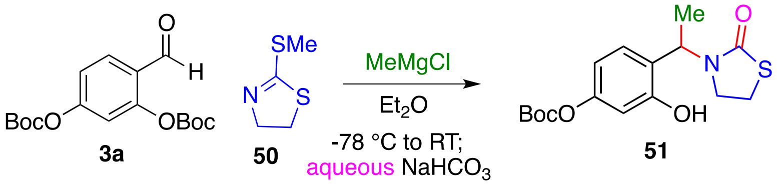

To a flame-dried Schlenk tube was charged with aldehyde **3a** solution (0.1 mL, 0.1 mmol, 1.0 M in toluene) and dry Et_2O (1 mL). Methylmagnesium chloride (0.04 mL, 0,105 mmol, 2.6M solution in THF, 1.05 equiv) was added to the mixture at -78 ºC. The mixture was allowed to stir at the same

temperature for 10 minutes before addition of **50** solution (0.2 mL, 0.2 mmol, 1.0 M in toluene, 2 equiv.). The mixture was allowed to warm to room temperature over 16 hours. The solution was quenched with saturated $NaHCO_3$ solution (1 mL), extracted with Et_2O (3 × 1mL). Organic layers were combined, washed with brine (1 mL), dried over anhydrous Na_2SO_4, and concentrated. The crude product was purified by column chromatography (SiO_2, eluent: hexanes/ethyl acetate = 5:1→1:1).

Tert-butyl (3-hydroxy-4-(1-(2-oxothiazolidin-3-yl)ethyl)phenyl) carbonate **(50)**.

White solid (24.2 mg, 71% isolated yield). **^{1}H NMR** (600 MHz, $CDCl_3$): δ 7.18 (d, *J* = 8.7 Hz, 1H), 6.78 (d, *J* = 2.4 Hz, 1H), 6.71 (dd, *J* = 8.3, 2.4 Hz, 1H), 5.51 (q, *J* = 7.0 Hz, 1H), 3.74 (ddd, *J* = 9.6, 8.2, 4.5 Hz, 1H), 3.41-3.34 (m, 1H), 3.30 (dt, *J* = 10.7, 8.4 Hz, 1H), 3.17 (ddd, *J* = 10.8, 8.0, 4.5 Hz, 1H), 1.62 (d, *J* = 7.3 Hz, 3H), 1.55 (s, 9H). **^{13}C{^{1}H} NMR** (126 MHz, $CDCl_3$): δ 175.2, 156.1, 151.9, 151.7, 126.7, 122.5, 112.7, 111.3, 83.7, 46.0, 43.8, 27.7, 25.8, 16.6. **HRMS (ESI)** m/z calculated for $C_{16}H_{21}NO_5SNa$ $[M+Na]^+$: 362.1038; found 362.1056. **R_f** = 0.1 (hexanes/ethyl acetate = 3:1).

7. Potential Application Toward Mariline B

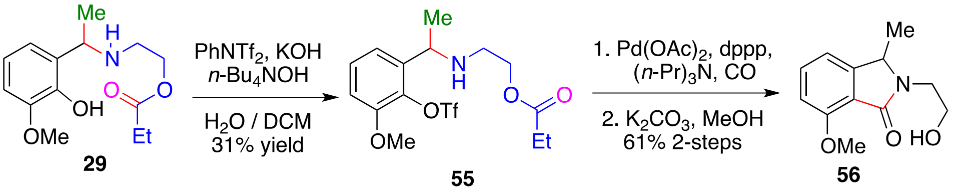

To a solution of adduct **29** (15.0 mg, 0.1 mmol) in dichloromethane (1 mL) was added 2M NaOH solution (0.5 mL), followed by tetrabutylammonium hydroxide (5.2 mg, 10 mol%, 50% solution in water) and bis(trifluoromethanesulfonyl)aniline (53.6 mg, 0.15 mmol, 1.5 equiv). The biphasic mixture was stirred at room temperature for 16 hours. The mixture was then diluted with more dichloromethane (2 mL). The aqueous layer was extracted with dichloromethane (3 × 2 mL). The combined organic layers was dried over anhydrous Na_2SO_4, concentrated *in vacuo*. The residue was purified by column chromatography (SiO_2, eluent: hexanes:ethyl acetate = 10:1→5:1) to afford impure **55** as yellow oil (12.4 mg, 31% yield). A Schlenk tube was charged with **55** (12.4 mg, 0.031 mmol) in toluene (0.5 mL) under nitrogen atmosphere. To this solution was added tri-*n*-propylamine (8.9 mg, 0.062 mmol, 2.0 equiv), $Pd(OAc)_2$ (0.7 mg, 0.0031 mmol, 10 mol%) and dppp (1.3 mg, 0.0031 mmol, 10 mol%). The solution was purged with a balloon of carbon monoxide gas for 3 minutes, after which the balloon was left attached to the Schlenk tube. The solution was heated at 100 ºC for 16 hours. After cooling to room temperature, the mixture was diluted with ethyl acetate (1 mL) and water (1 mL). Aqueous layer was extracted with ethyl acetate (3 × 1 mL). The organic layers were combined, washed with brine (1 mL), dried over anhydrous Na_2SO_4, and concentrated *in vacuo*. The residue was taken up in methanol (1 mL), potassium carbonate (12.9 mg, 0.093 mmol, 3 equiv) was added. The suspension was stirred for 30 minutes, after which the suspension was filtered. The filtrate collected was concentrated and purified by column chromatography (SiO_2, eluent: hexanes/ethyl acetate = 1:1→100% ethyl acetate) to afford **56** as yellow oil (4.2 mg, 61% isolated yield).

2-(2-Hydroxyethyl)-7-methoxy-3-methylisoindolin-1-one (56). **^{1}H NMR** (600 MHz, $CDCl_3$): δ 7.52-7.46 (m, 1H), 6.96 (d, *J* = 7.7 Hz, 1H), 6.89 (d, *J* = 8.3 Hz, 1H), 4.53 (q, *J* = 6.6 Hz, 1H), 3.97 (s, 3H), 3.91-3.83 (m, 2H), 3.81-3.75 (m, 1H), 3.57 (ddd, *J* = 14.6, 6.4, 3.7 Hz, 1H), 1.46 (d, *J* = 6.6 Hz, 3H). **^{13}C{^{1}H} NMR** (126 MHz, $CDCl_3$): δ 157.3, 150.0, 136.3, 133.5, 118.5, 114.0, 110.2, 62.6, 57.1, 55.8, 44.8, 18.5. **HRMS (ESI)** m/z calculated for $C_{12}H_{15}NO_3Na$ $[M+Na]^+$: 244.0950; found 244.0939. R_f = 0.4 (hexanes/ethyl acetate = 1:3).

8. NMR Spectra

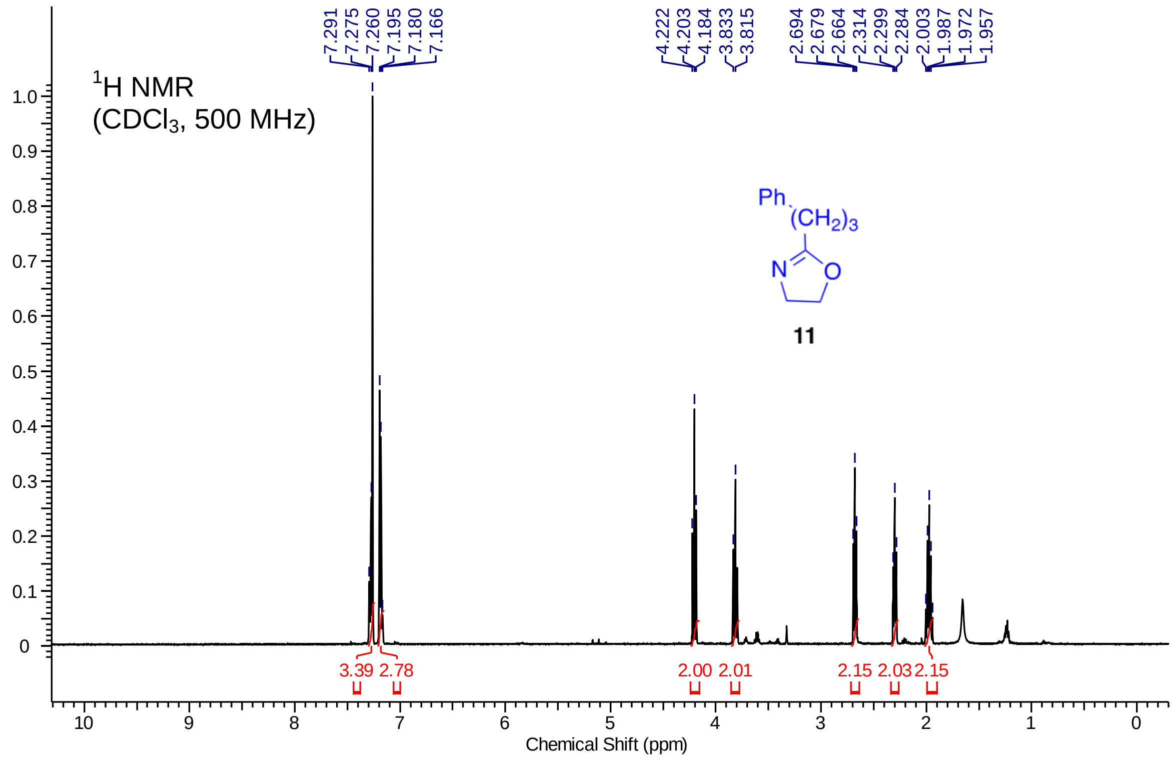

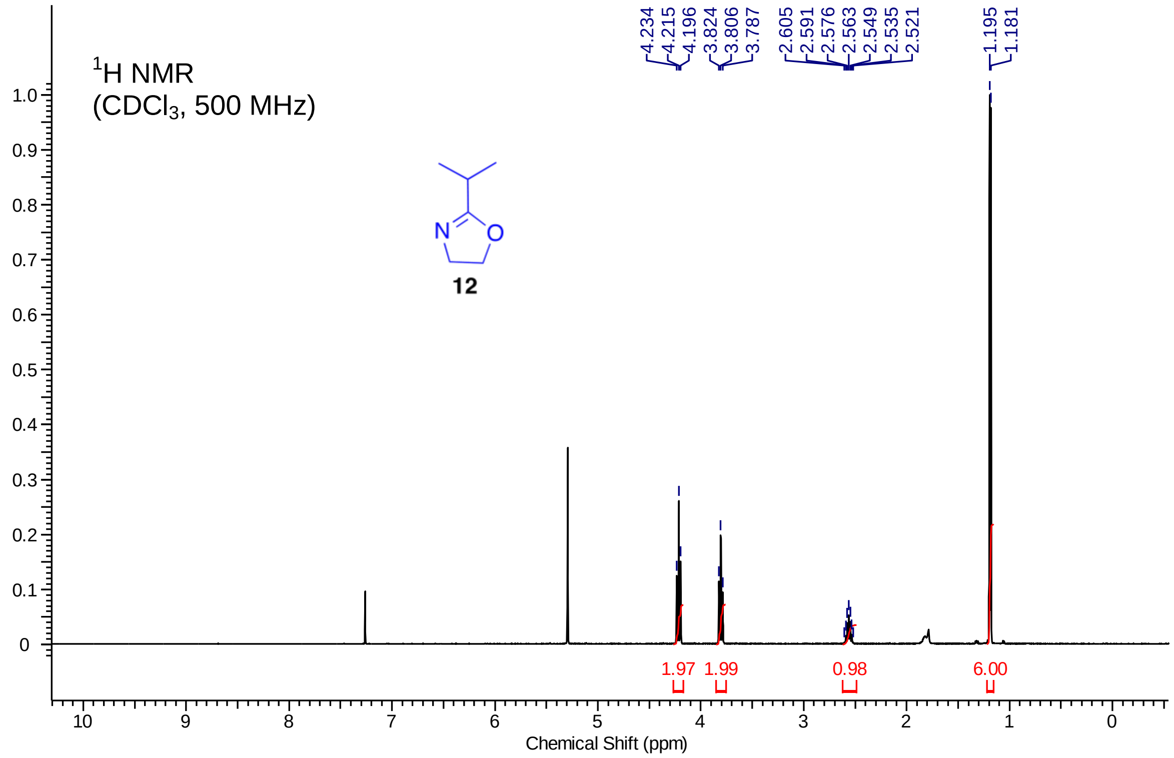

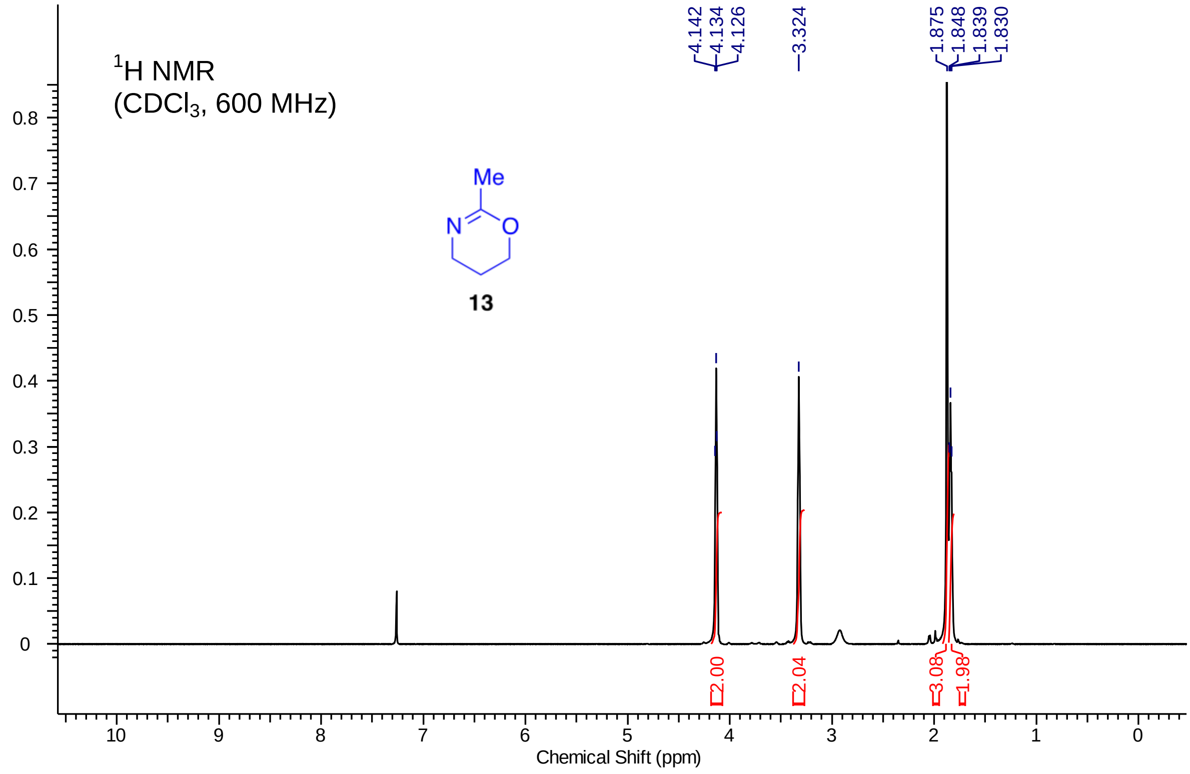

4.142
4.134
4.126
3.324
1.875
1.848
1.839
1.830
^{1}H NMR
($CDCl_3$, 600 MHz)
Me
N
O
13
2.00
2.04
3.08
1.98
Chemical Shift (ppm)

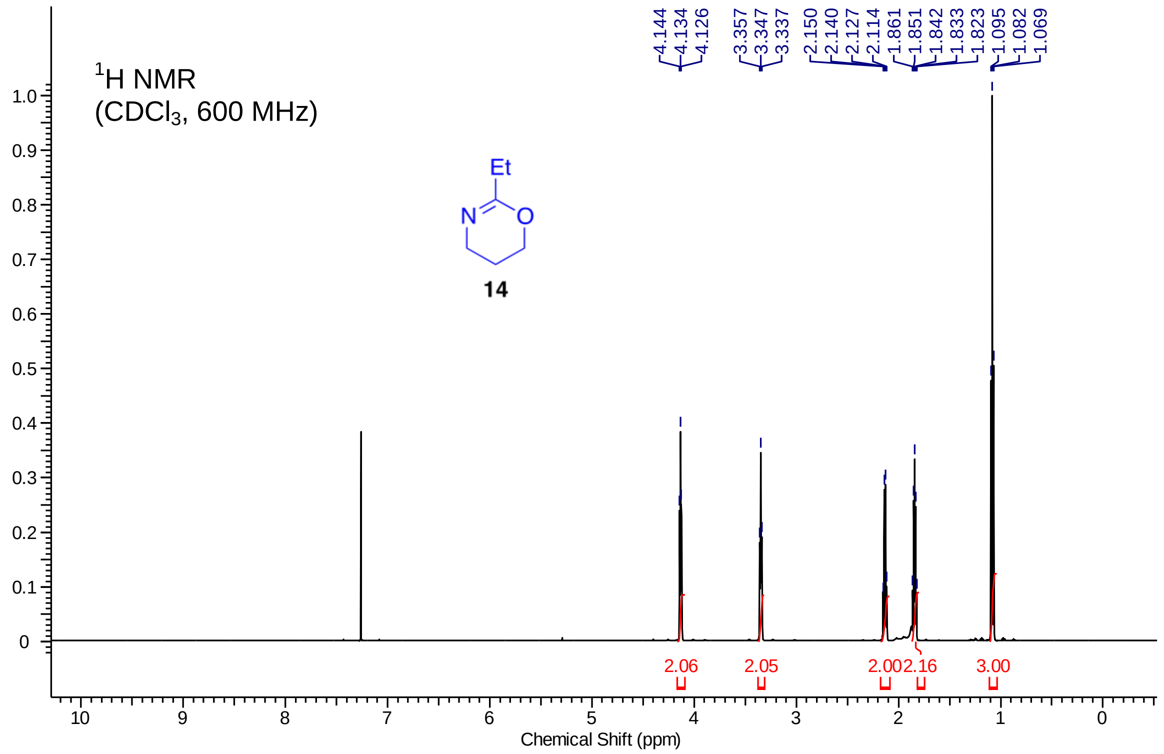

4.144
4.134
4.126
3.357
3.347
3.337
2.150
2.140
2.127
2.114
1.861
1.851
1.842
1.833
1.823
1.095
1.082
1.069
^{1}H NMR
($CDCl_3$, 600 MHz)
Et
N
O
14
2.06
2.05
2.00
2.16
3.00
Chemical Shift (ppm)

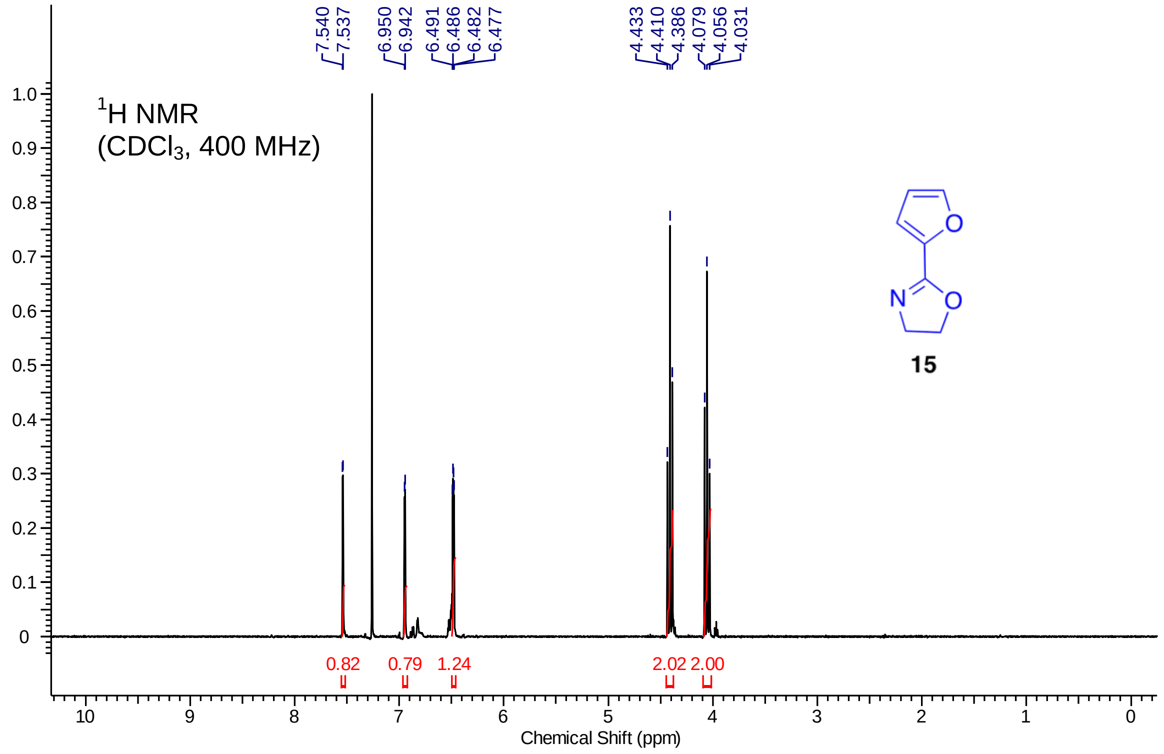

7.540
7.537
6.950
6.942
6.491
6.486
6.482
6.477
4.433
4.410
4.386
4.079
4.056
4.031
1H NMR
(CDCl3, 400 MHz)
O
N
O
15
0.82
0.79
1.24
2.02
2.00
Chemical Shift (ppm)

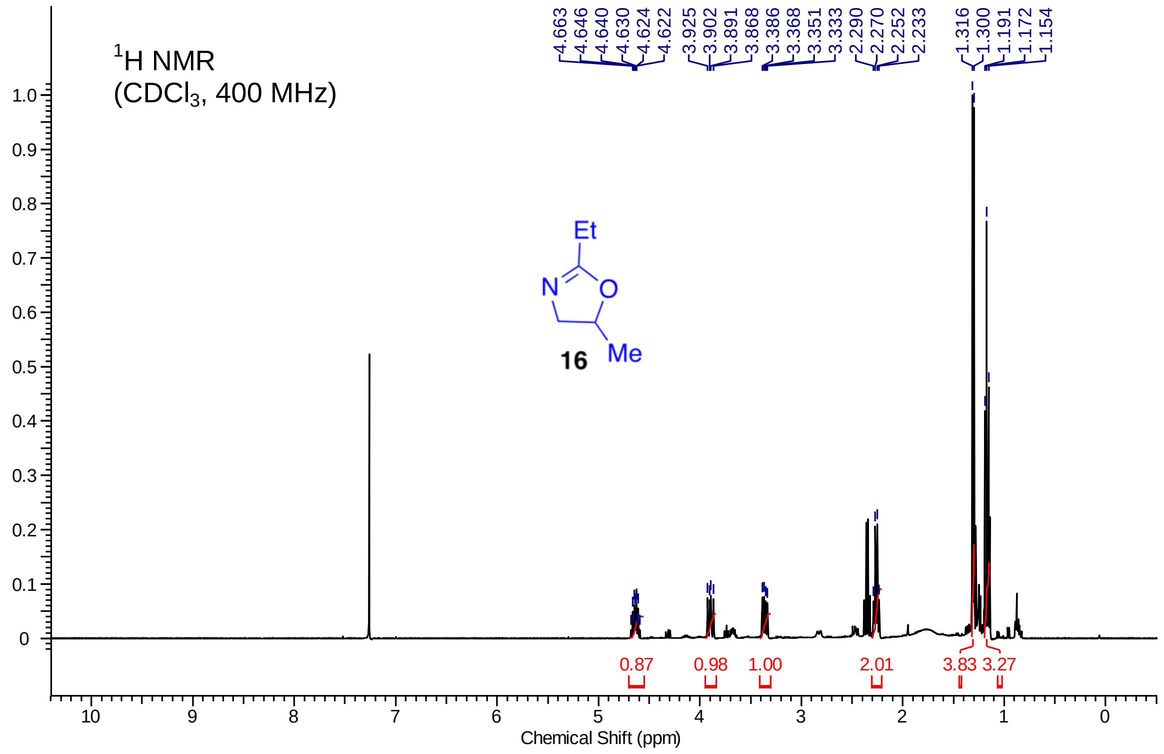

4.663
4.646
4.640
4.630
4.624
4.622
3.925
3.902
3.891
3.868
3.386
3.368
3.351
3.333
2.290
2.270
2.252
2.233
1.316
1.300
1.191
1.172
1.154
1H NMR
(CDCl3, 400 MHz)
Et
N
O
16
Me
0.87
0.98
1.00
2.01
3.83
3.27
Chemical Shift (ppm)

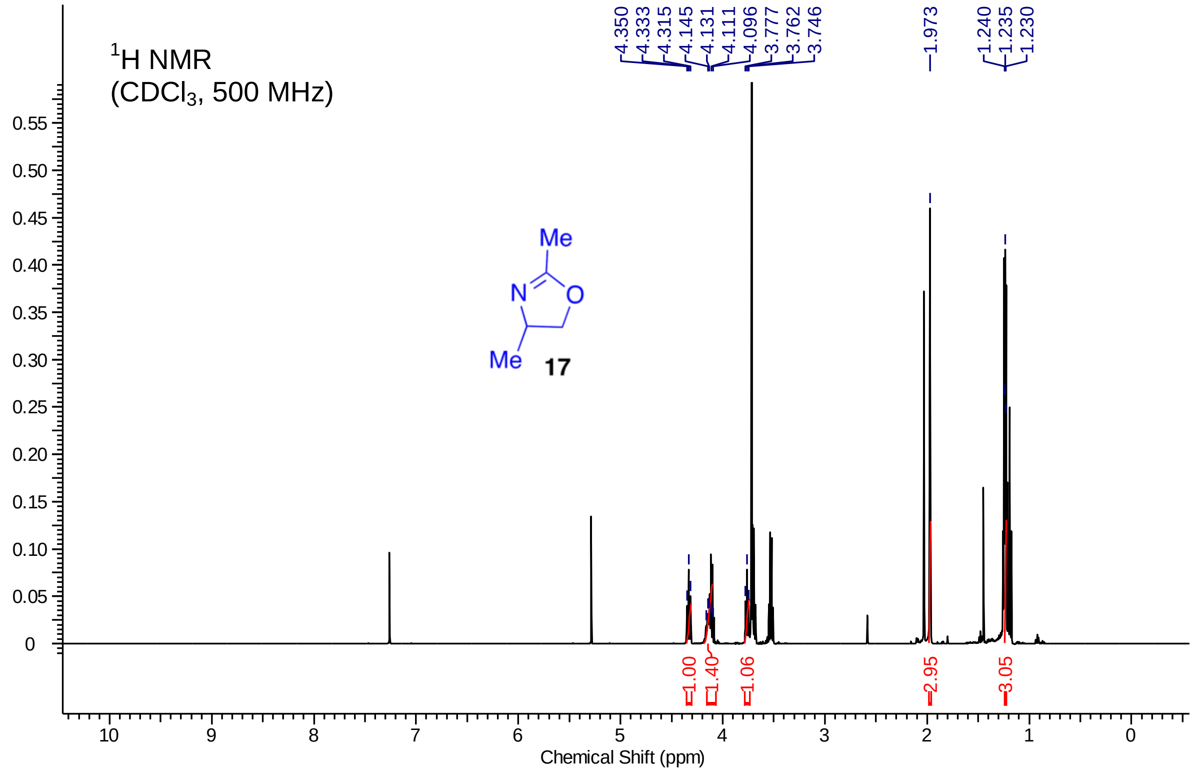

1H NMR
(CDCl3, 500 MHz)
4.350
4.333
4.315
4.145
4.131
4.111
4.096
3.777
3.762
3.746
1.973
1.240
1.235
1.230
Me
N
O
Me
17
1.00
1.40
1.06
2.95
3.05
0.55
0.50
0.45
0.40
0.35
0.30
0.25
0.20
0.15
0.10
0.05
0
10
9
8
7
6
5
4
3
2
1
0
Chemical Shift (ppm)

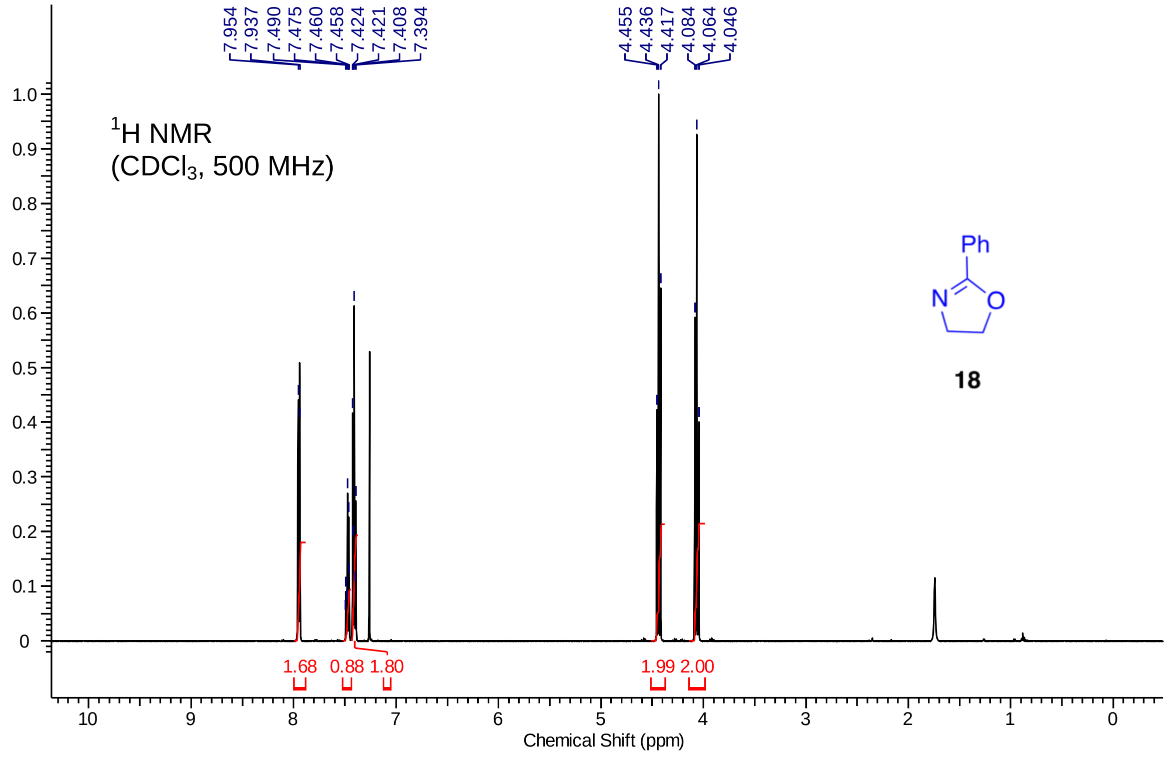

1H NMR
(CDCl3, 500 MHz)
7.954
7.937
7.490
7.475
7.460
7.458
7.424
7.421
7.408
7.394
4.455
4.436
4.417
4.084
4.064
4.046
Ph
N
O
18
1.68
0.88
1.80
1.99
2.00
1.0
0.9
0.8
0.7
0.6
0.5
0.4
0.3
0.2
0.1
0
10
9
8
7
6
5
4
3
2
1
0
Chemical Shift (ppm)

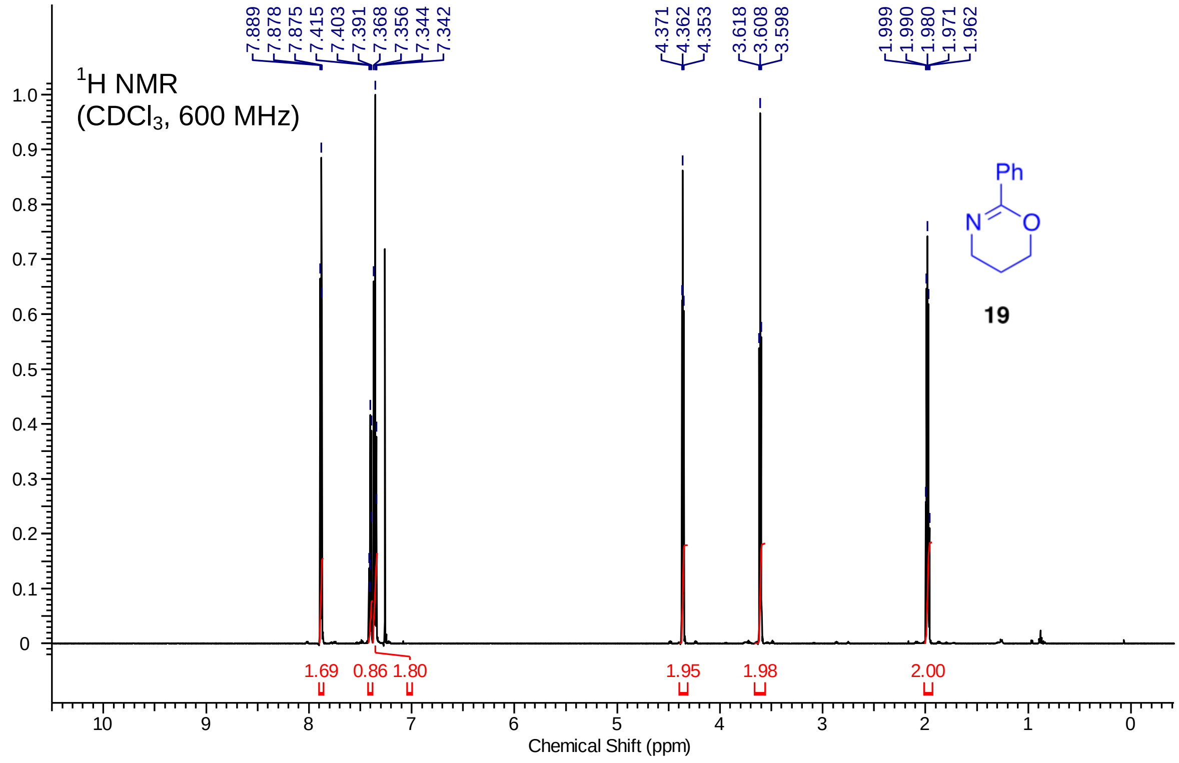
7.889
7.878
7.875
7.415
7.403
7.391
7.368
7.356
7.344
7.342
4.371
4.362
4.353
3.618
3.608
3.598
1.999
1.990
1.980
1.971
1.962
^{1}H NMR
($CDCl_3$, 600 MHz)
Ph
N
O
19
1.69
0.86
1.80
1.95
1.98
2.00
Chemical Shift (ppm)

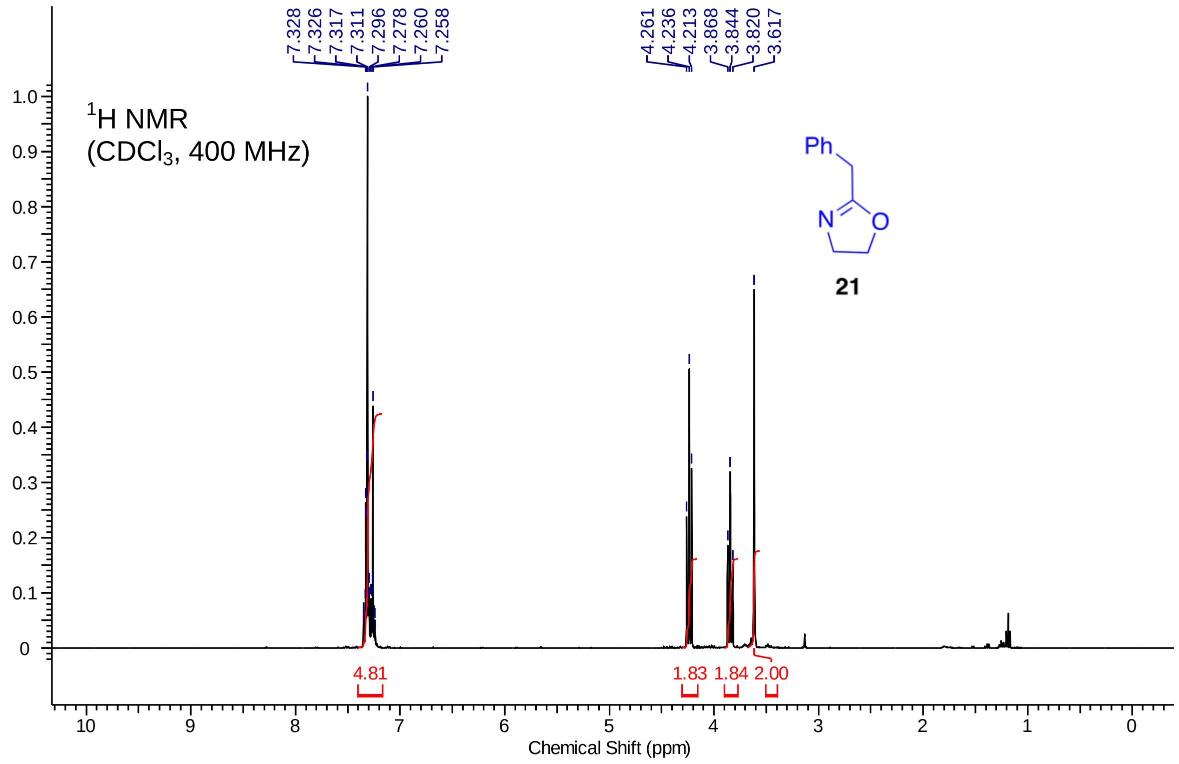
7.328
7.326
7.317
7.311
7.296
7.278
7.260
7.258
4.261
4.236
4.213
3.868
3.844
3.820
3.617
^{1}H NMR
($CDCl_3$, 400 MHz)
Ph
N
O
21
4.81
1.83
1.84
2.00
Chemical Shift (ppm)

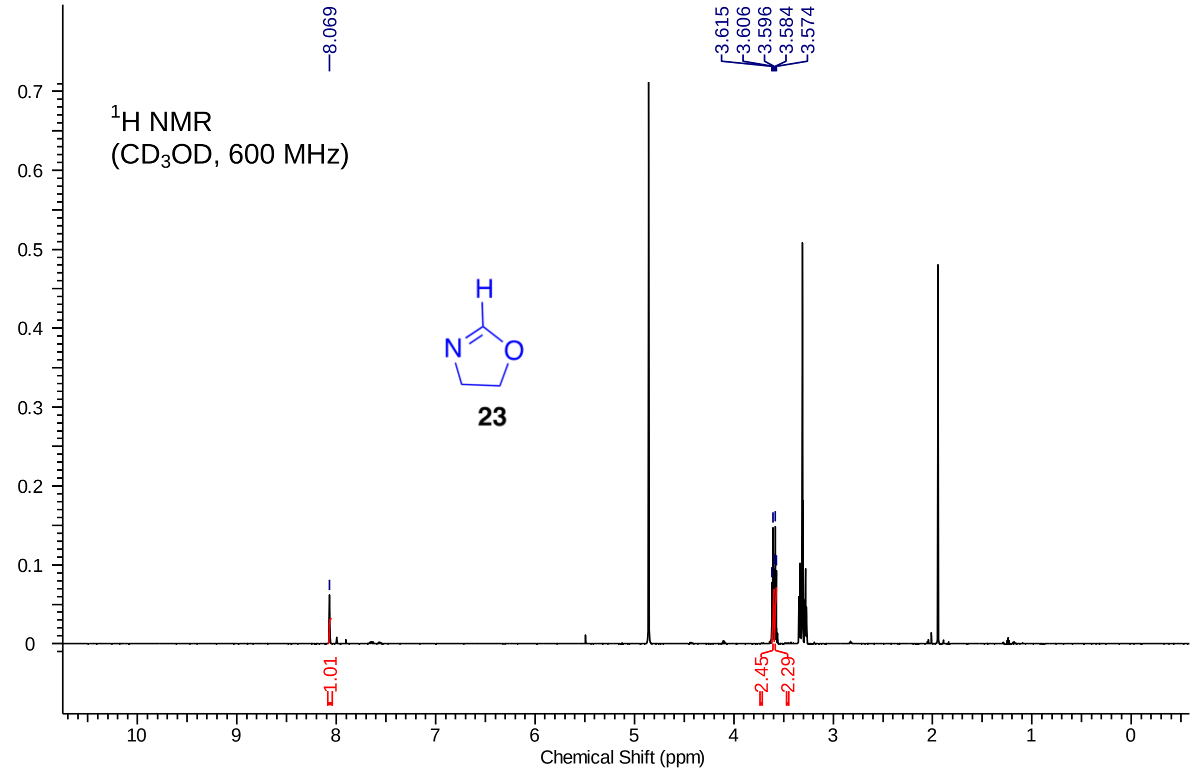

8.069
3.615
3.606
3.596
3.584
3.574
^1H NMR
(CD_3OD, 600 MHz)
H
N
O
23
1.01
2.45
2.29
Chemical Shift (ppm)

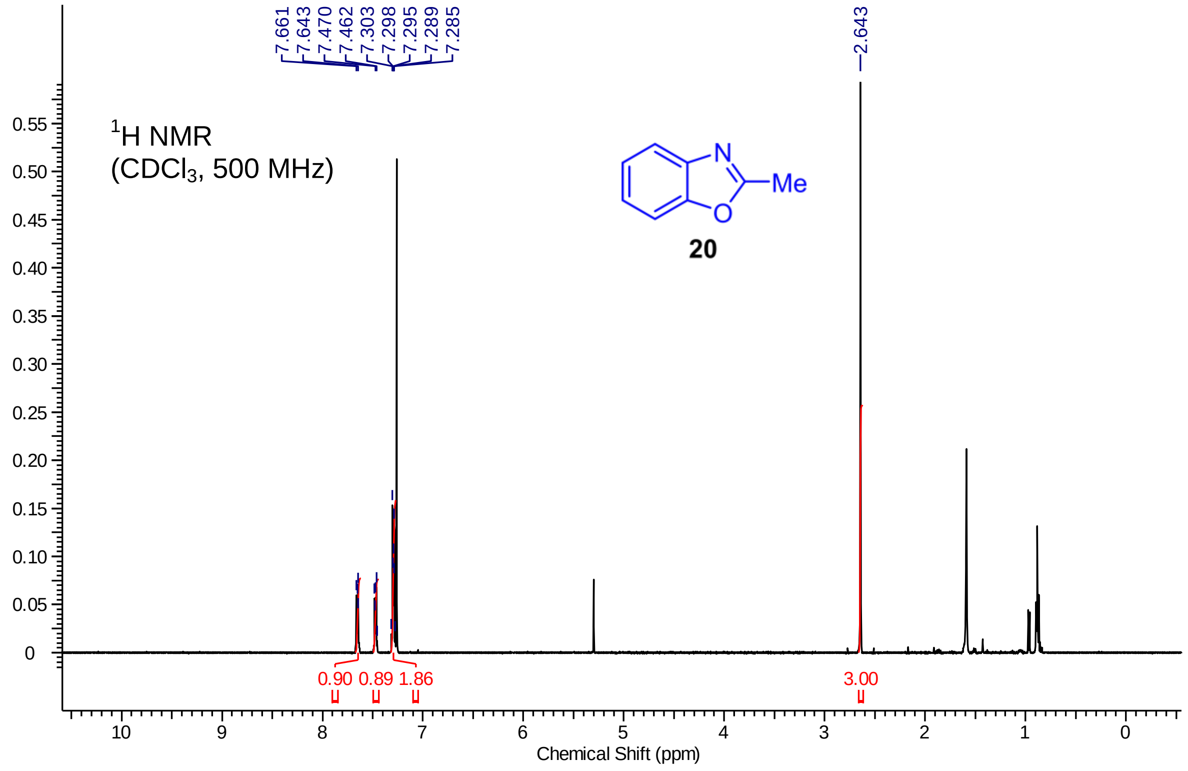

7.661
7.643
7.470
7.462
7.303
7.298
7.295
7.289
7.285
2.643
^1H NMR
($CDCl_3$, 500 MHz)
N
O
Me
20
0.90
0.89
1.86
3.00
Chemical Shift (ppm)

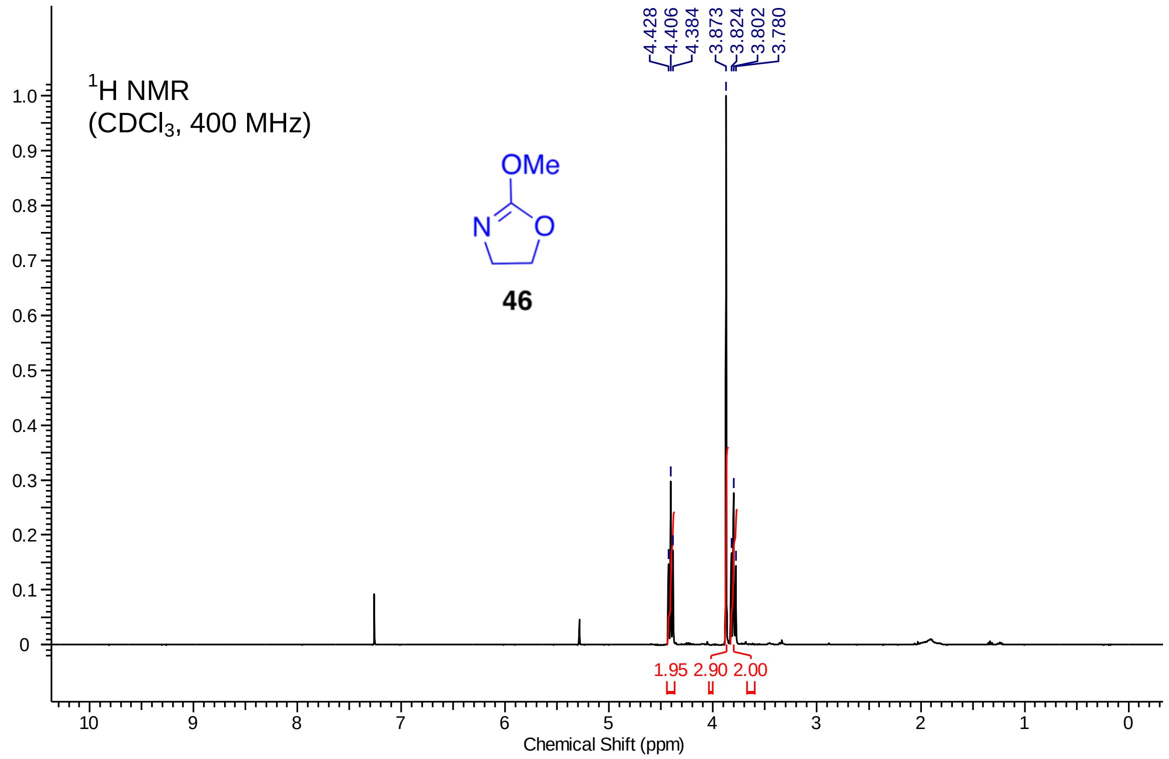
4.428
4.406
4.384
3.873
3.824
3.802
3.780
1H NMR
(CDCl3, 400 MHz)
OMe
N
O
46
1.95
2.90
2.00
Chemical Shift (ppm)

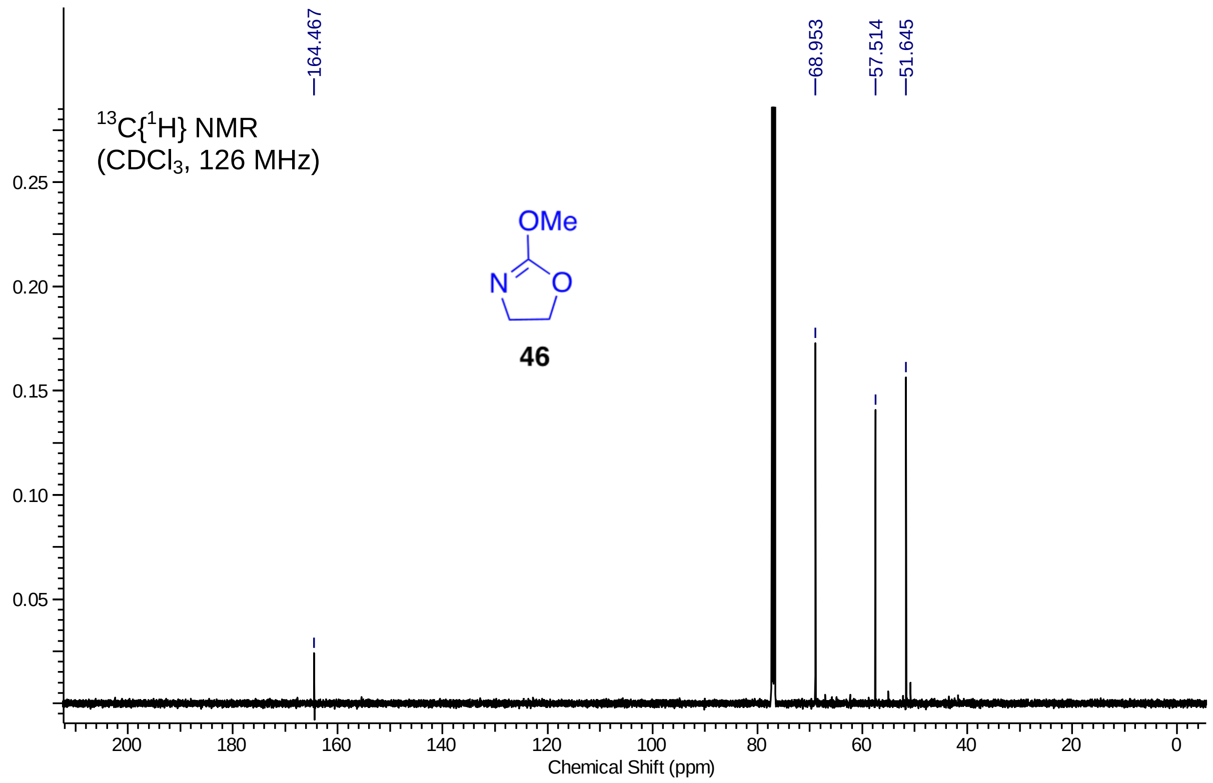
164.467
68.953
57.514
51.645
13C{1H} NMR
(CDCl3, 126 MHz)
OMe
N
O
46
Chemical Shift (ppm)

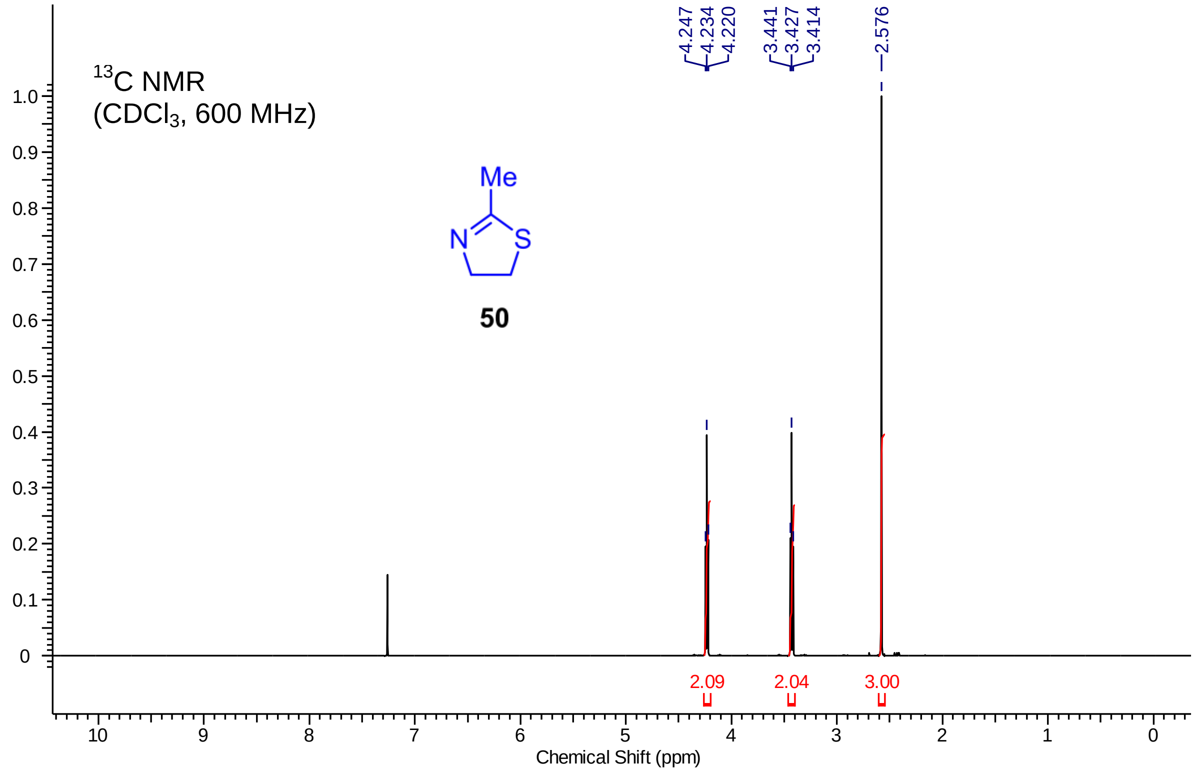
13C NMR
(CDCl3, 600 MHz)
Me
N
S
50
4.247
4.234
4.220
3.441
3.427
3.414
2.576
2.09
2.04
3.00
Chemical Shift (ppm)

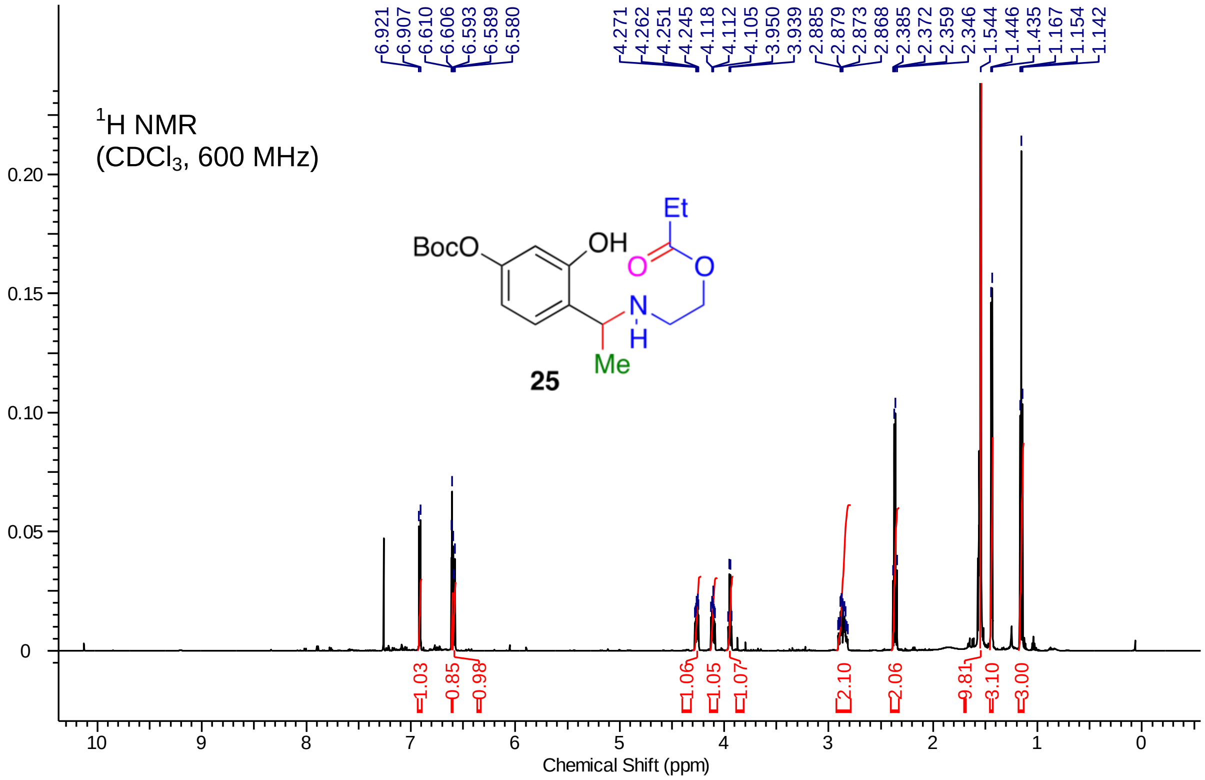

6.921
6.907
6.610
6.606
6.593
6.589
6.580
4.271
4.262
4.251
4.245
4.118
4.112
4.105
3.950
3.939
2.885
2.879
2.873
2.868
2.385
2.372
2.359
2.346
1.544
1.446
1.435
1.167
1.154
1.142
1H NMR
(CDCl3, 600 MHz)
BocO
OH
Et
O
O
N
H
Me
25
1.03
0.85
0.98
1.06
1.05
1.07
2.10
2.06
9.81
3.10
3.00
Chemical Shift (ppm)

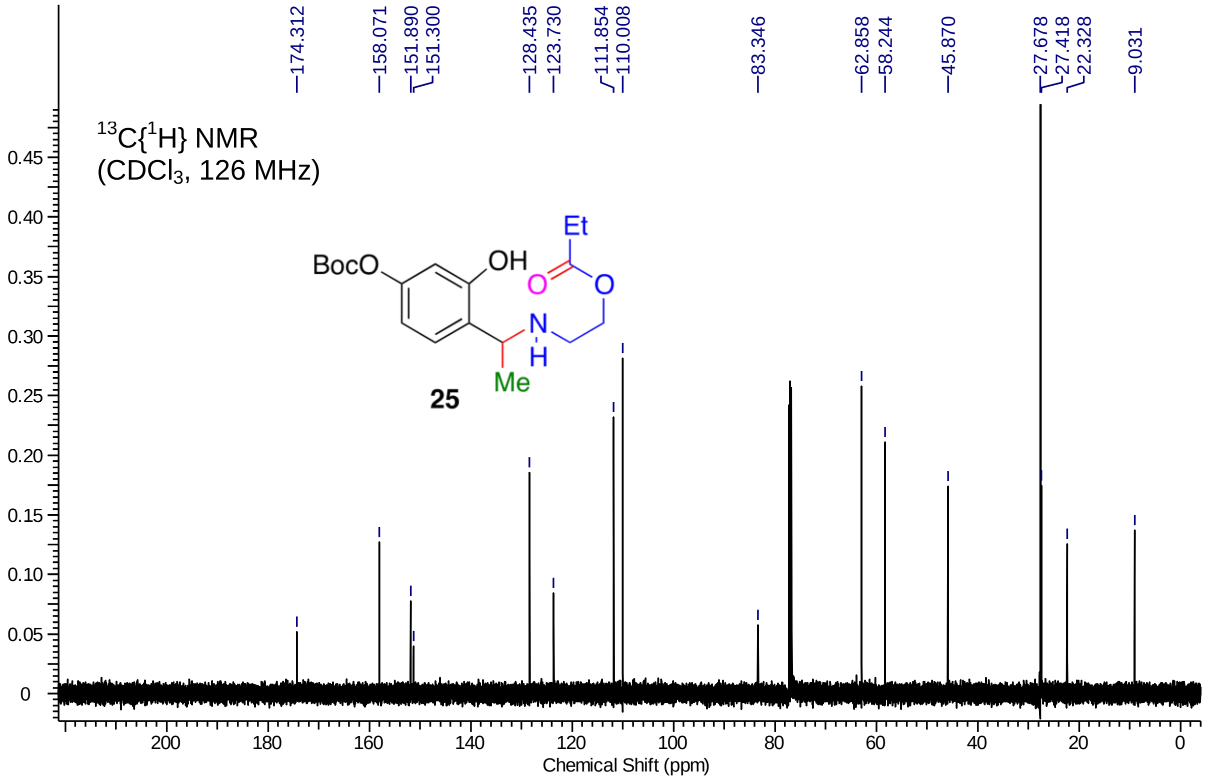

174.312
158.071
151.890
151.300
128.435
123.730
111.854
110.008
83.346
62.858
58.244
45.870
27.678
27.418
22.328
9.031
13C{1H} NMR
(CDCl3, 126 MHz)
BocO
OH
Et
O
O
N
H
Me
25
Chemical Shift (ppm)

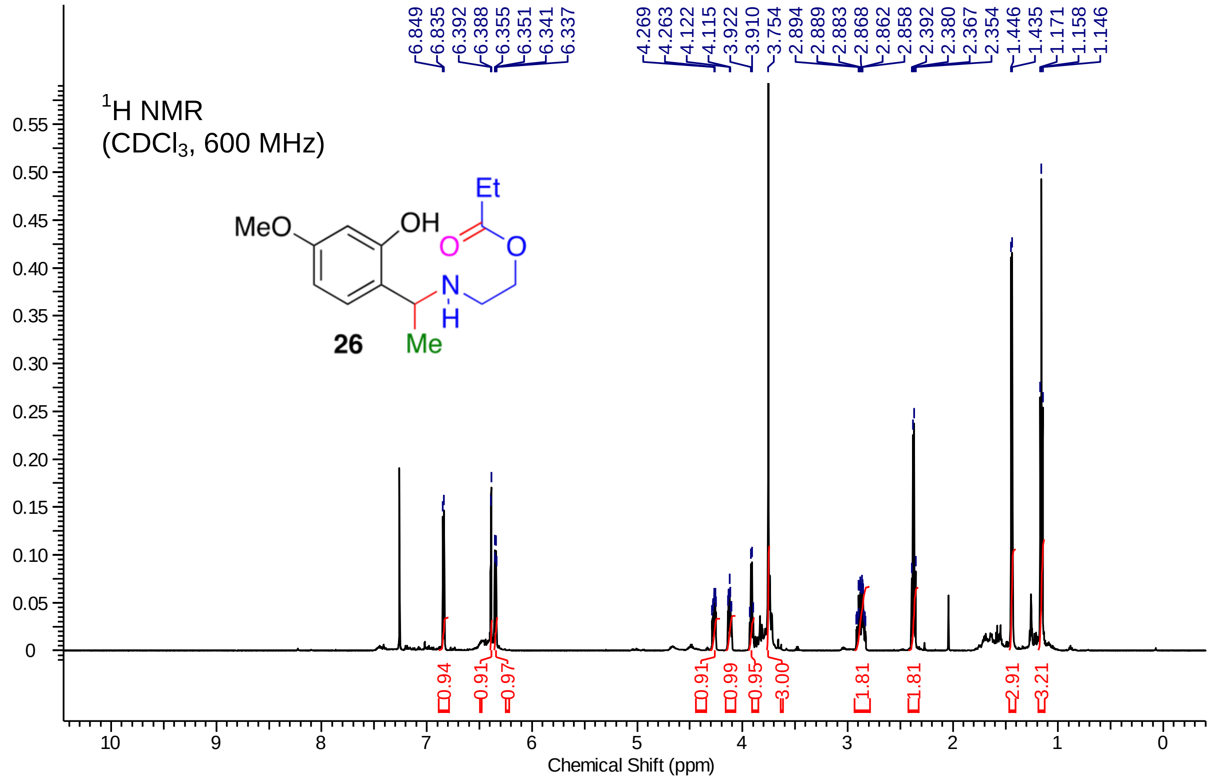

6.849
6.835
6.392
6.388
6.355
6.351
6.341
6.337
4.269
4.263
4.122
4.115
3.922
3.910
3.754
2.894
2.889
2.883
2.868
2.862
2.858
2.392
2.380
2.367
2.354
1.446
1.435
1.171
1.158
1.146
^{1}H NMR
($CDCl_3$, 600 MHz)
MeO
OH
Et
O
O
N
H
26
Me
0.94
0.91
0.97
0.91
0.99
0.95
3.00
1.81
1.81
2.91
3.21
Chemical Shift (ppm)

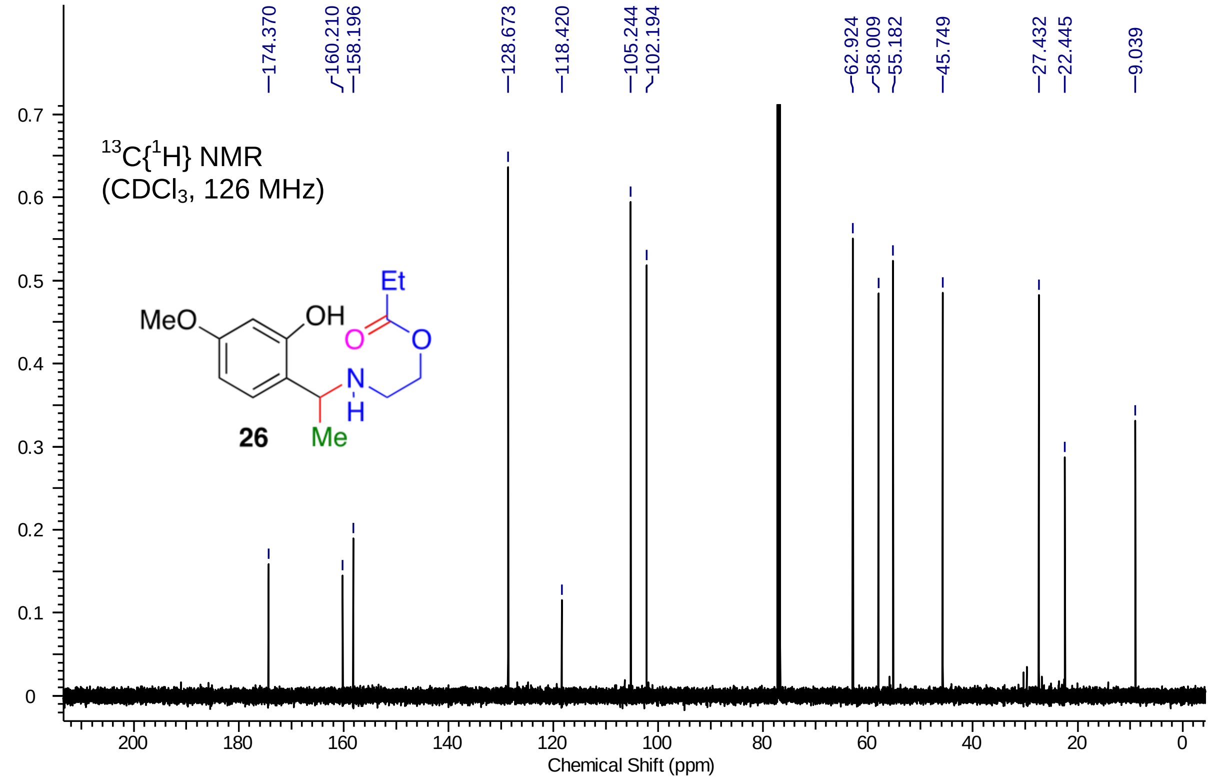

174.370
160.210
158.196
128.673
118.420
105.244
102.194
62.924
58.009
55.182
45.749
27.432
22.445
9.039
^{13}C{^{1}H} NMR
($CDCl_3$, 126 MHz)
MeO
OH
Et
O
O
N
H
26
Me
Chemical Shift (ppm)

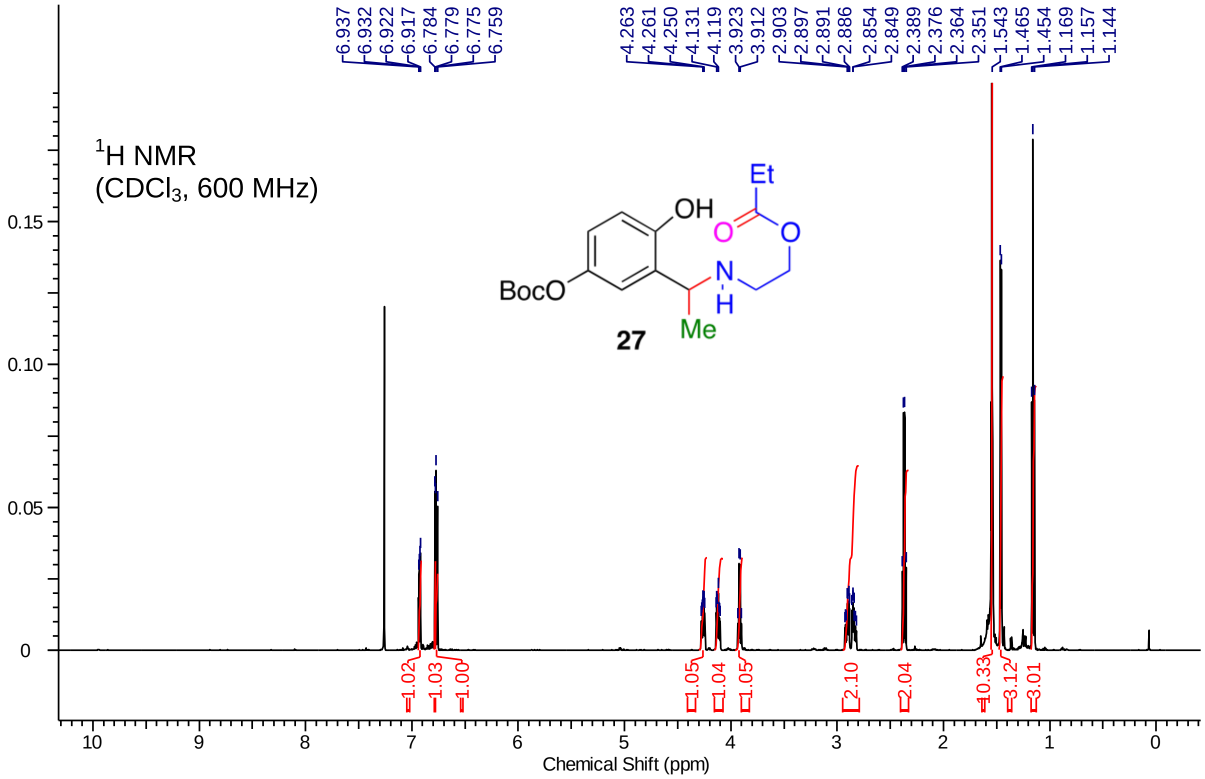
6.937
6.932
6.922
6.917
6.784
6.779
6.775
6.759
4.263
4.261
4.250
4.131
4.119
3.923
3.912
2.903
2.897
2.891
2.886
2.854
2.849
2.389
2.376
2.364
2.351
1.543
1.465
1.454
1.169
1.157
1.144
1H NMR
(CDCl3, 600 MHz)
OH
Et
O
O
N
H
BocO
Me
27
1.02
1.03
1.00
1.05
1.04
1.05
2.10
2.04
10.33
3.12
3.01
Chemical Shift (ppm)

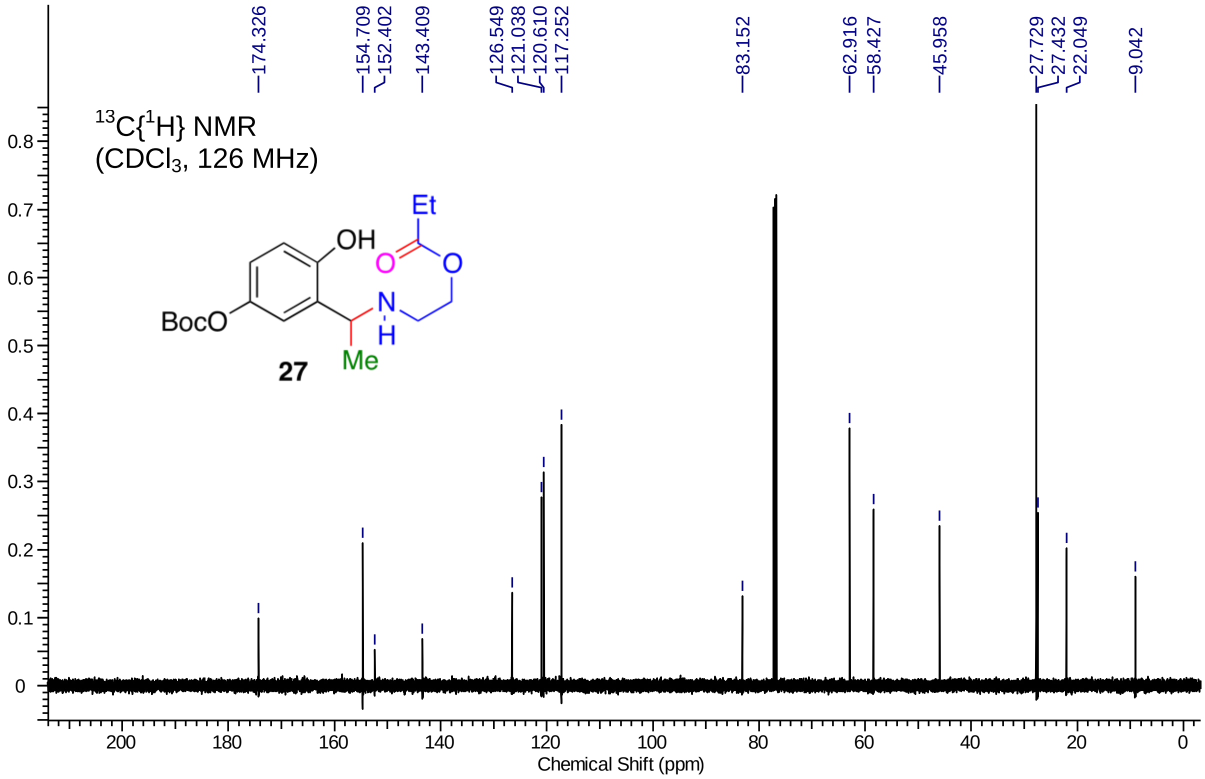
174.326
154.709
152.402
143.409
126.549
121.038
120.610
117.252
83.152
62.916
58.427
45.958
27.729
27.432
22.049
9.042
13C{1H} NMR
(CDCl3, 126 MHz)
OH
Et
O
O
N
H
BocO
Me
27
Chemical Shift (ppm)

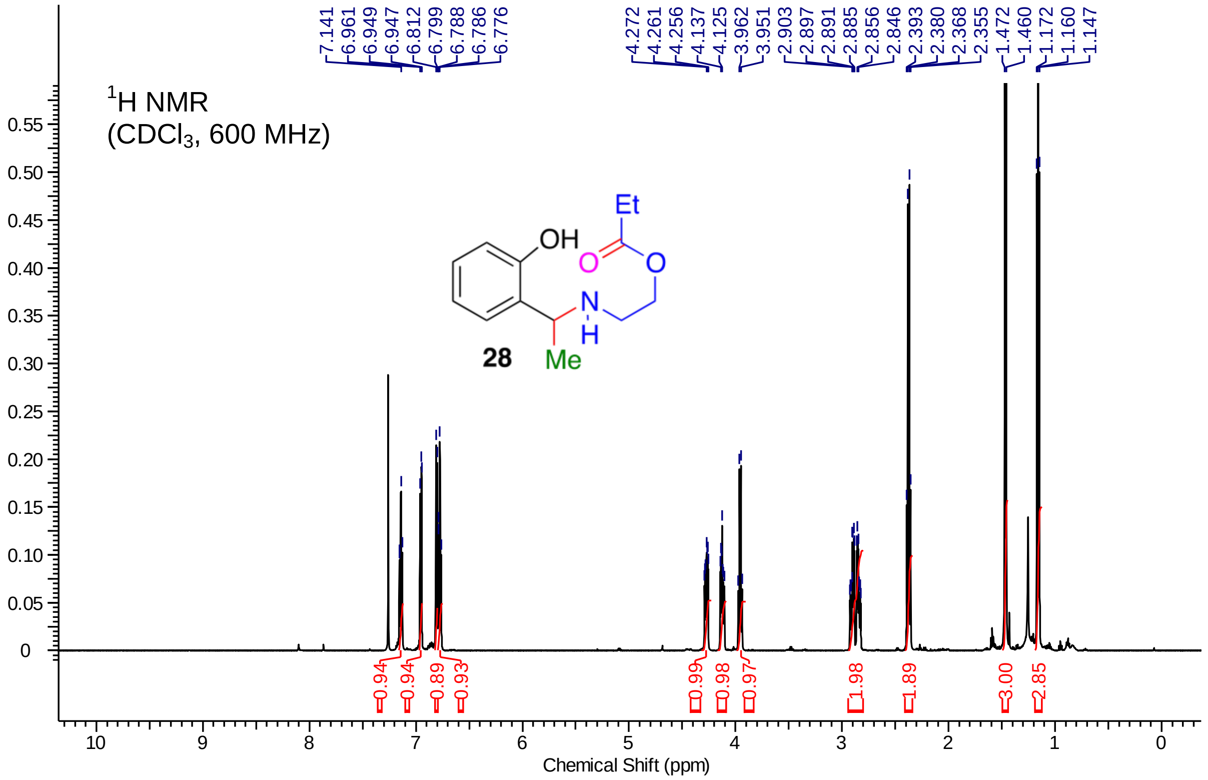
7.141
6.961
6.949
6.947
6.812
6.799
6.788
6.786
6.776
4.272
4.261
4.256
4.137
4.125
3.962
3.951
2.903
2.897
2.891
2.885
2.856
2.846
2.393
2.380
2.368
2.355
1.472
1.460
1.172
1.160
1.147
1H NMR
(CDCl3, 600 MHz)
OH
Et
O
O
N
H
28
Me
0.94
0.94
0.89
0.93
0.99
0.98
0.97
1.98
1.89
3.00
2.85
Chemical Shift (ppm)

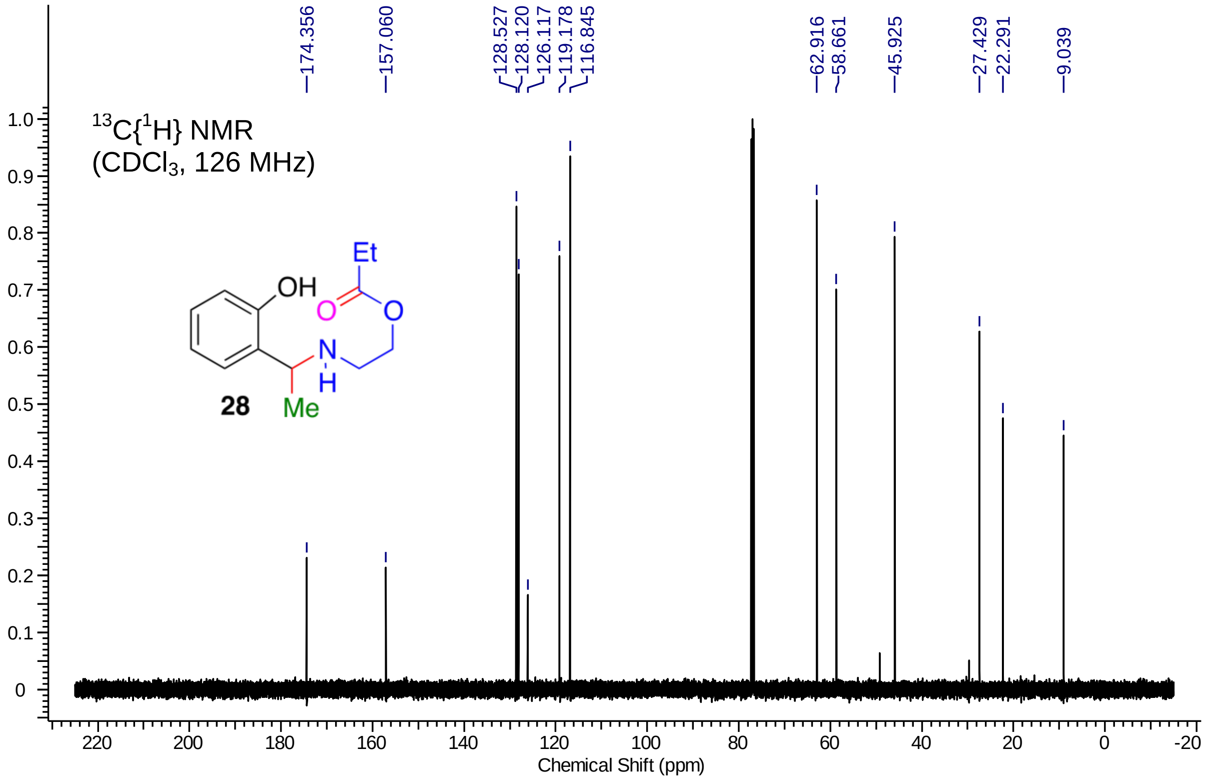
174.356
157.060
128.527
128.120
126.117
119.178
116.845
62.916
58.661
45.925
27.429
22.291
9.039
13C{1H} NMR
(CDCl3, 126 MHz)
OH
Et
O
O
N
H
28
Me
Chemical Shift (ppm)

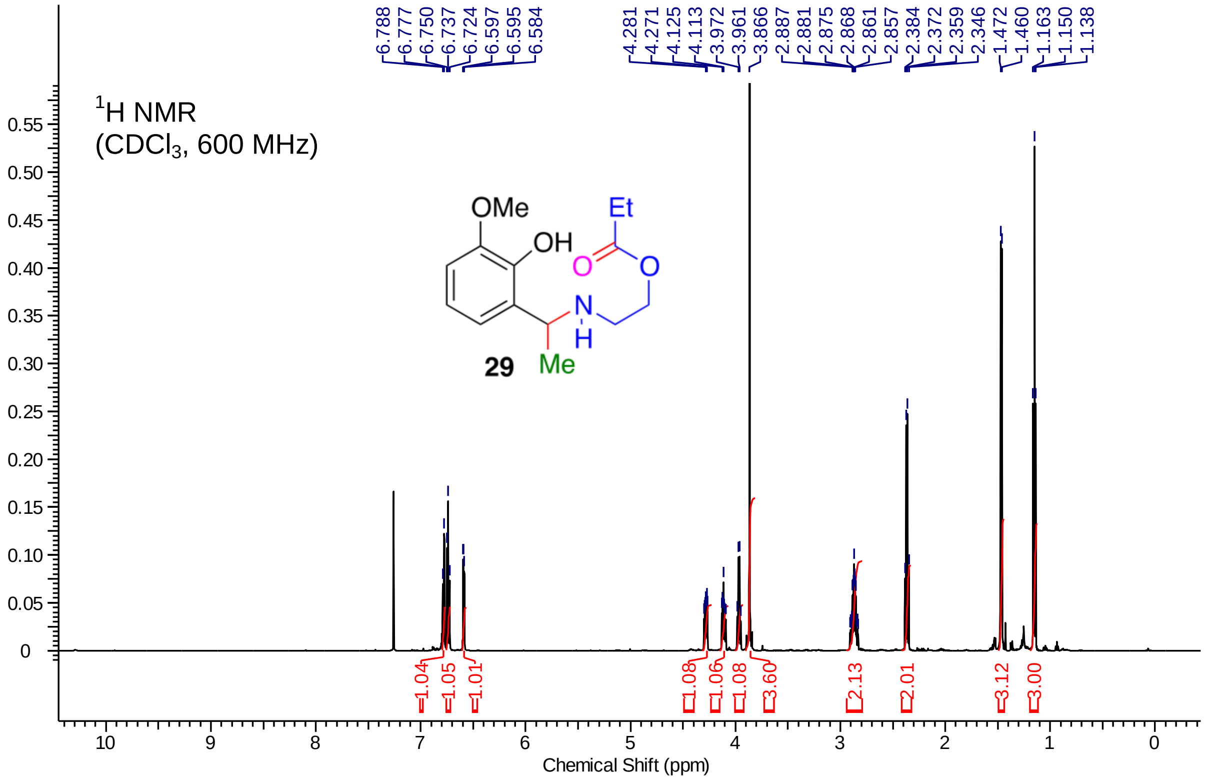

6.788
6.777
6.750
6.737
6.724
6.597
6.595
6.584
4.281
4.271
4.125
4.113
3.972
3.961
3.866
2.887
2.881
2.875
2.868
2.861
2.857
2.384
2.372
2.359
2.346
1.472
1.460
1.163
1.150
1.138
^{1}H NMR
($CDCl_3$, 600 MHz)
OMe
OH
Et
O
O
N
H
29
Me
1.04
1.05
1.01
1.08
1.06
1.08
3.60
2.13
2.01
3.12
3.00
Chemical Shift (ppm)

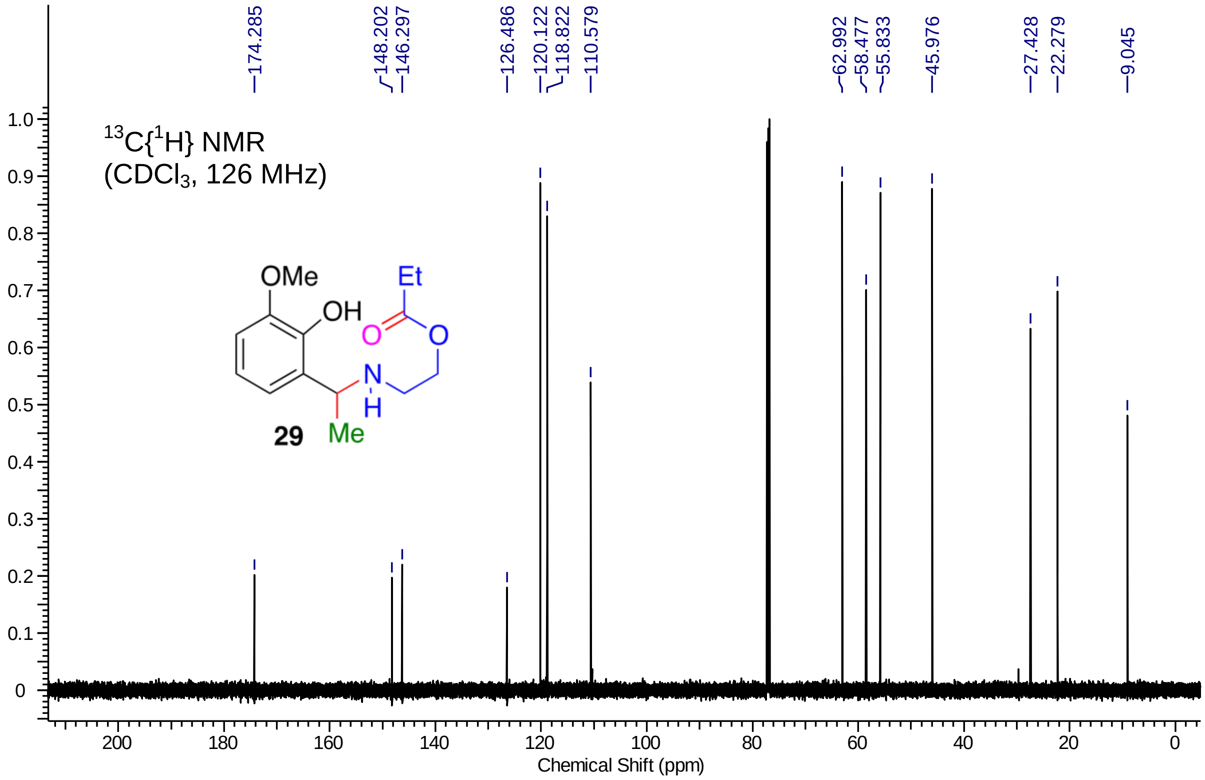

174.285
148.202
146.297
126.486
120.122
118.822
110.579
62.992
58.477
55.833
45.976
27.428
22.279
9.045
^{13}C{^{1}H} NMR
($CDCl_3$, 126 MHz)
OMe
OH
Et
O
O
N
H
29
Me
Chemical Shift (ppm)

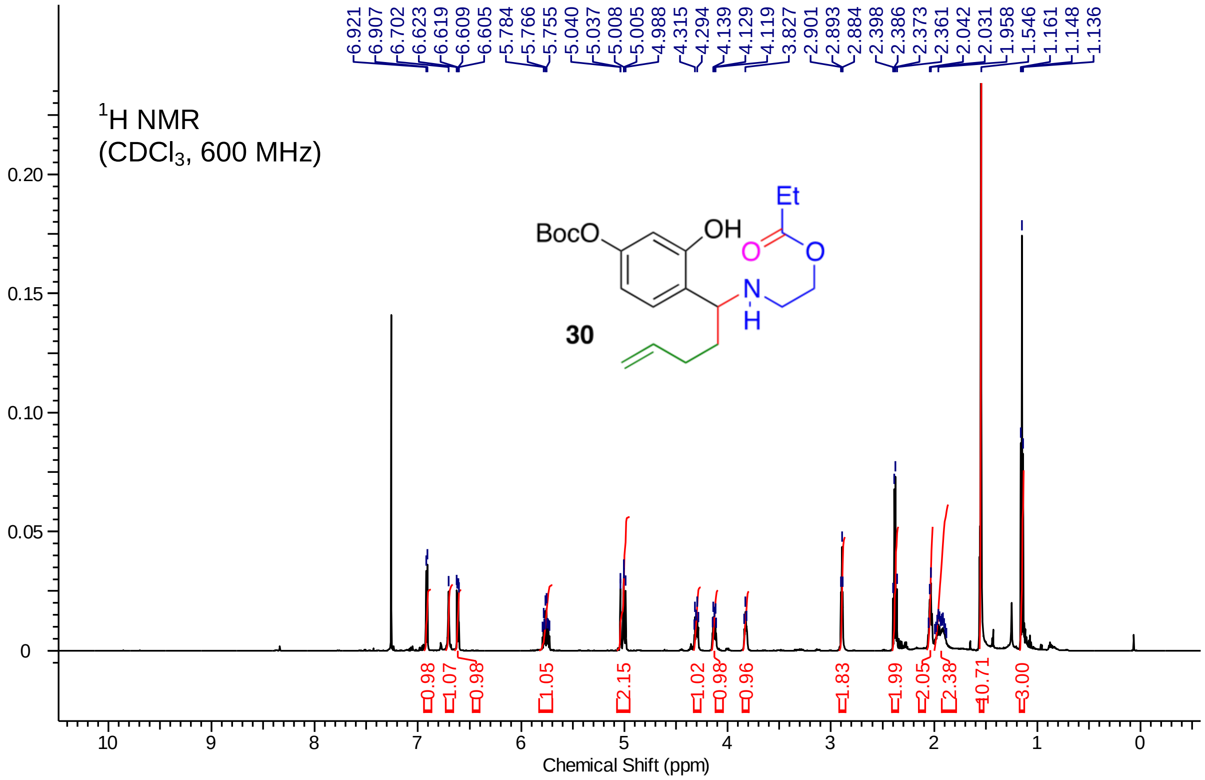
6.921
6.907
6.702
6.623
6.619
6.609
6.605
5.784
5.766
5.755
5.040
5.037
5.008
5.005
4.988
4.315
4.294
4.139
4.129
4.119
3.827
2.901
2.893
2.884
2.398
2.386
2.373
2.361
2.042
2.031
1.958
1.546
1.161
1.148
1.136
1H NMR
(CDCl3, 600 MHz)
BocO
OH
Et
O
O
N
H
30
0.98
1.07
0.98
1.05
2.15
1.02
0.98
0.96
1.83
1.99
2.05
2.38
10.71
3.00
Chemical Shift (ppm)

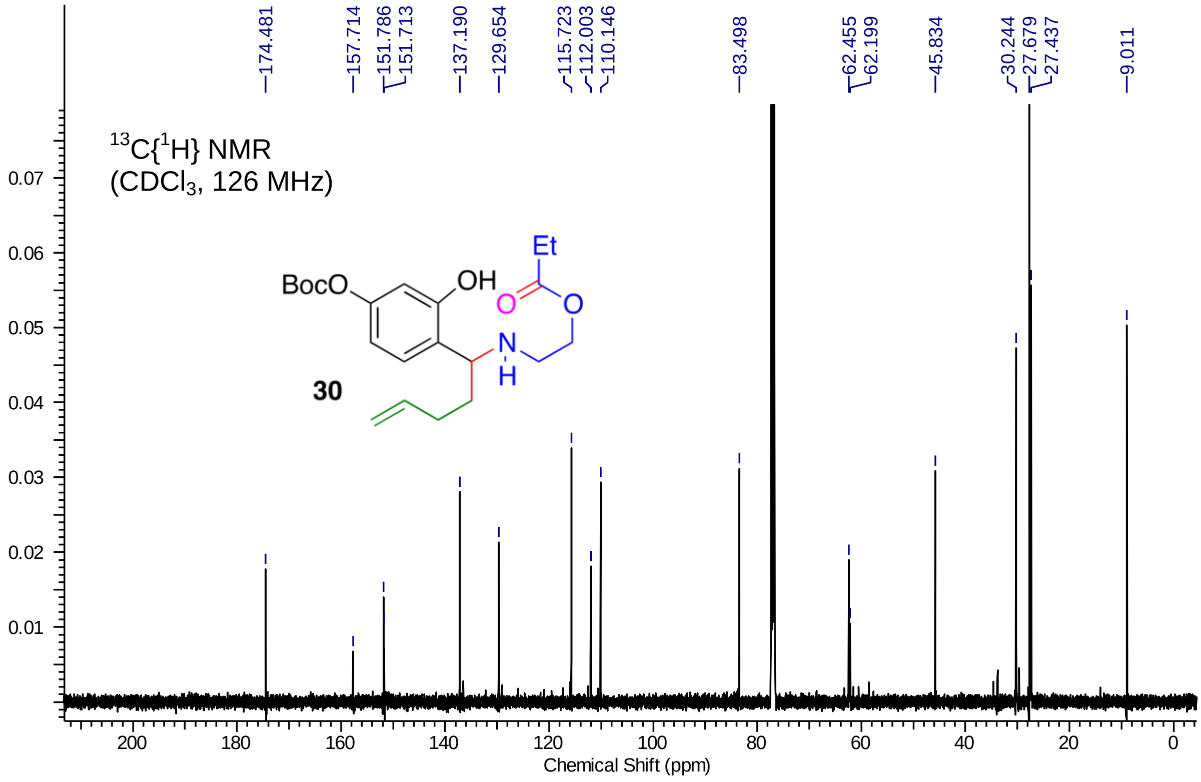
174.481
157.714
151.786
151.713
137.190
129.654
115.723
112.003
110.146
83.498
62.455
62.199
45.834
30.244
27.679
27.437
9.011
13C{1H} NMR
(CDCl3, 126 MHz)
BocO
OH
Et
O
O
N
H
30
Chemical Shift (ppm)

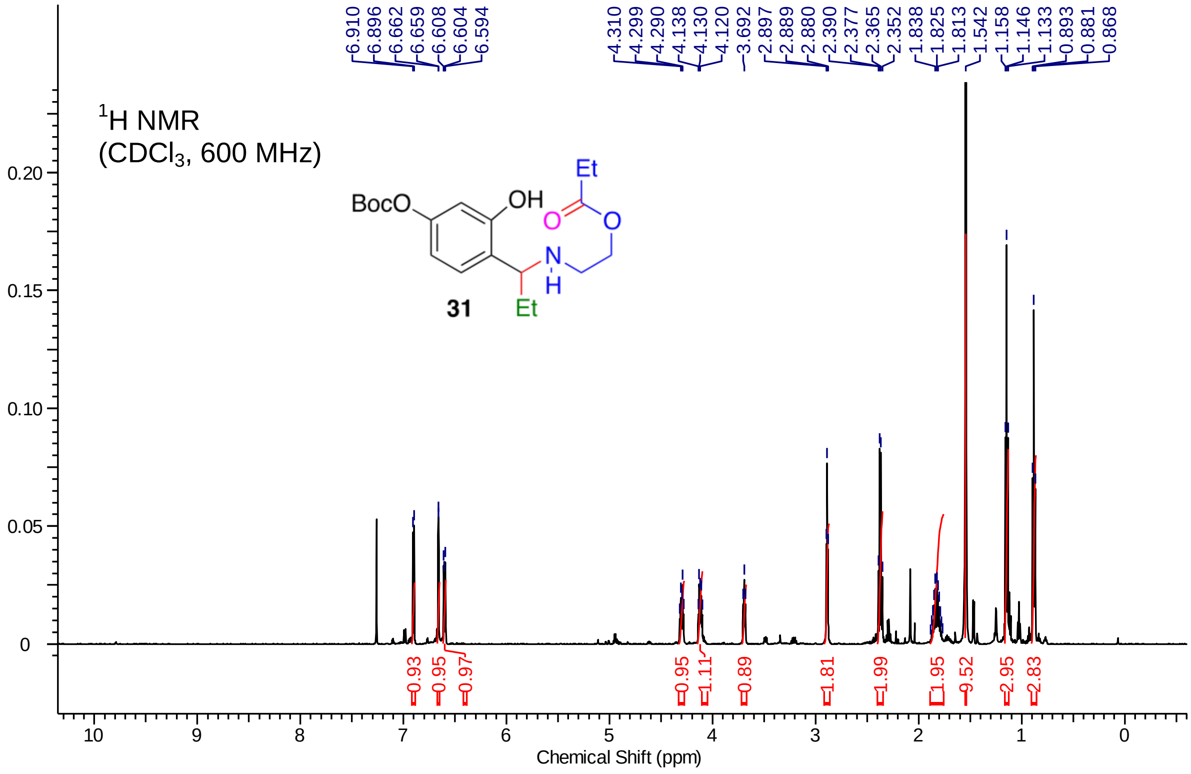
6.910
6.896
6.662
6.659
6.608
6.604
6.594
4.310
4.299
4.290
4.138
4.130
4.120
3.692
2.897
2.889
2.880
2.390
2.377
2.365
2.352
1.838
1.825
1.813
1.542
1.158
1.146
1.133
0.893
0.881
0.868
1H NMR
(CDCl3, 600 MHz)
BocO
OH
Et
O
O
N
H
31
Et
0.93
0.95
0.97
0.95
1.11
0.89
1.81
1.99
1.95
9.52
2.95
2.83
0.20
0.15
0.10
0.05
0
10
9
8
7
6
5
4
3
2
1
0
Chemical Shift (ppm)

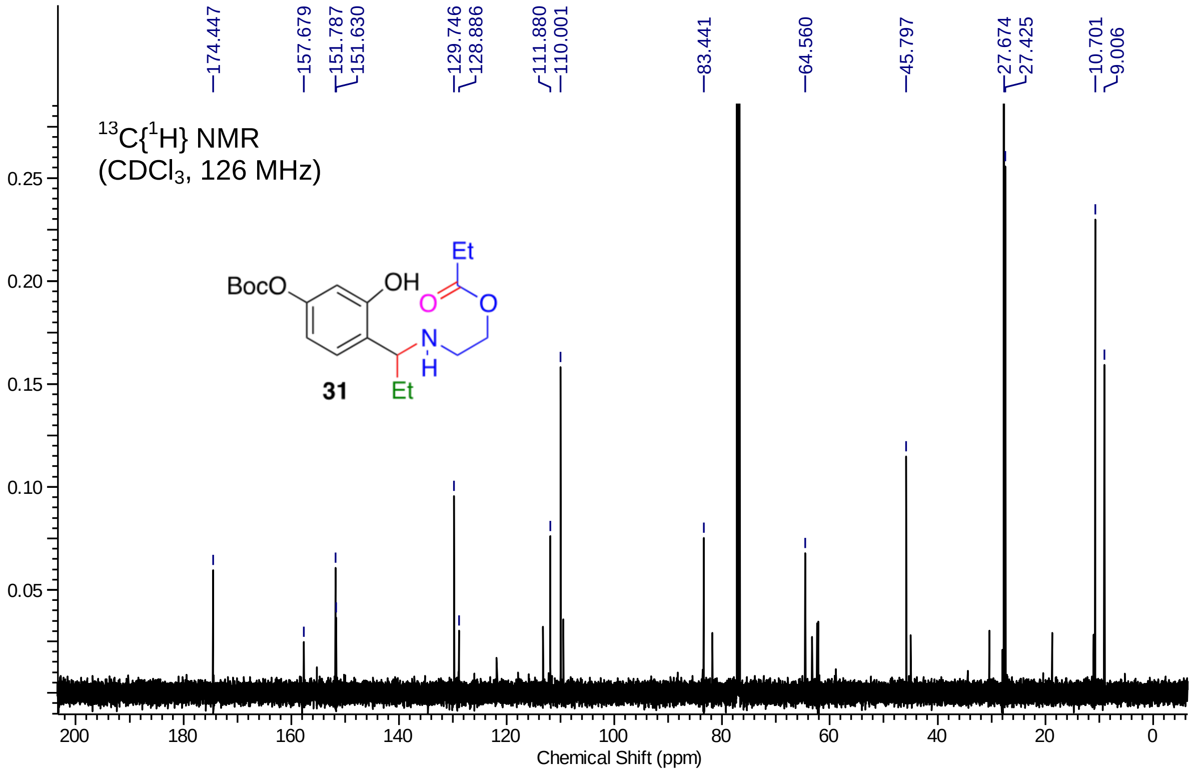
174.447
157.679
151.787
151.630
129.746
128.886
111.880
110.001
83.441
64.560
45.797
27.674
27.425
10.701
9.006
13C{1H} NMR
(CDCl3, 126 MHz)
BocO
OH
Et
O
O
N
H
31
Et
0.25
0.20
0.15
0.10
0.05
200
180
160
140
120
100
80
60
40
20
0
Chemical Shift (ppm)

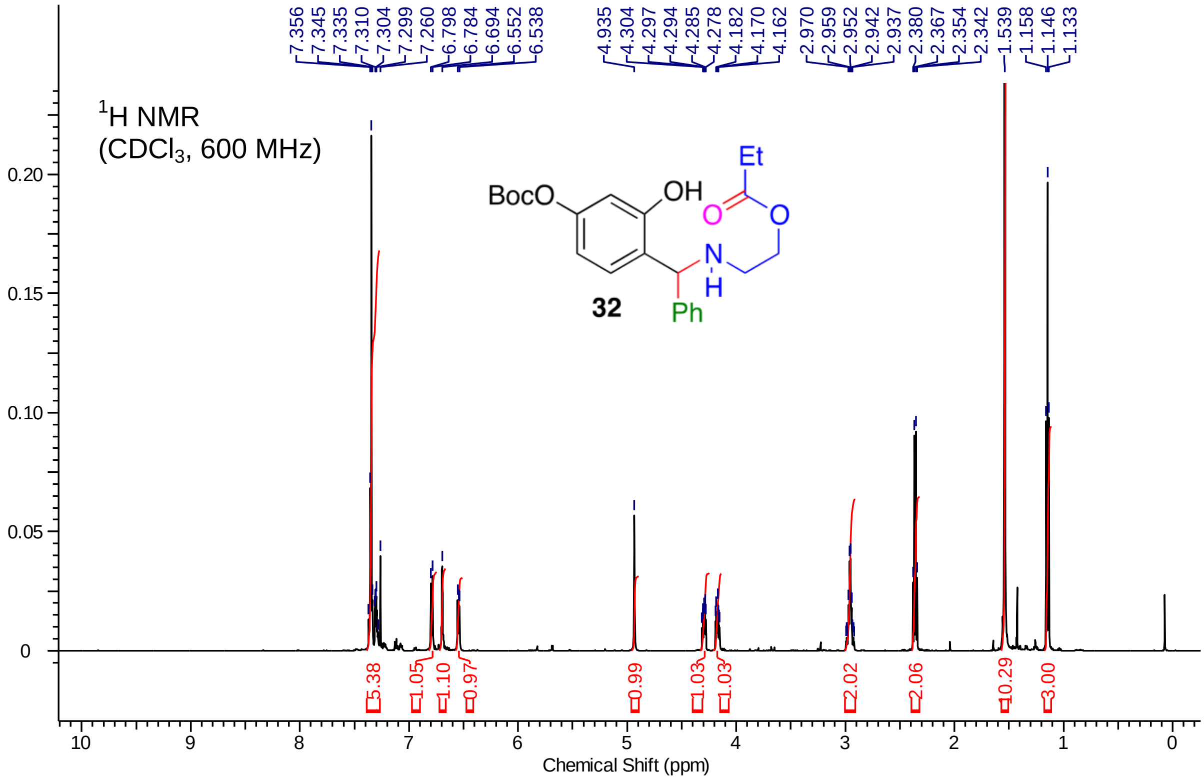

7.356 7.345 7.335 7.310 7.304 7.299 7.260 6.798 6.784 6.694 6.552 6.538
4.935 4.304 4.297 4.294 4.285 4.278 4.182 4.170 4.162 2.970 2.959 2.952 2.942 2.937 2.380 2.367 2.354 2.342 1.539 1.158 1.146 1.133
1H NMR
(CDCl3, 600 MHz)
BocO
OH
Et
O
O
N
H
32
Ph
5.38 1.05 1.10 0.97 0.99 1.03 1.03 2.02 2.06 10.29 3.00
Chemical Shift (ppm)

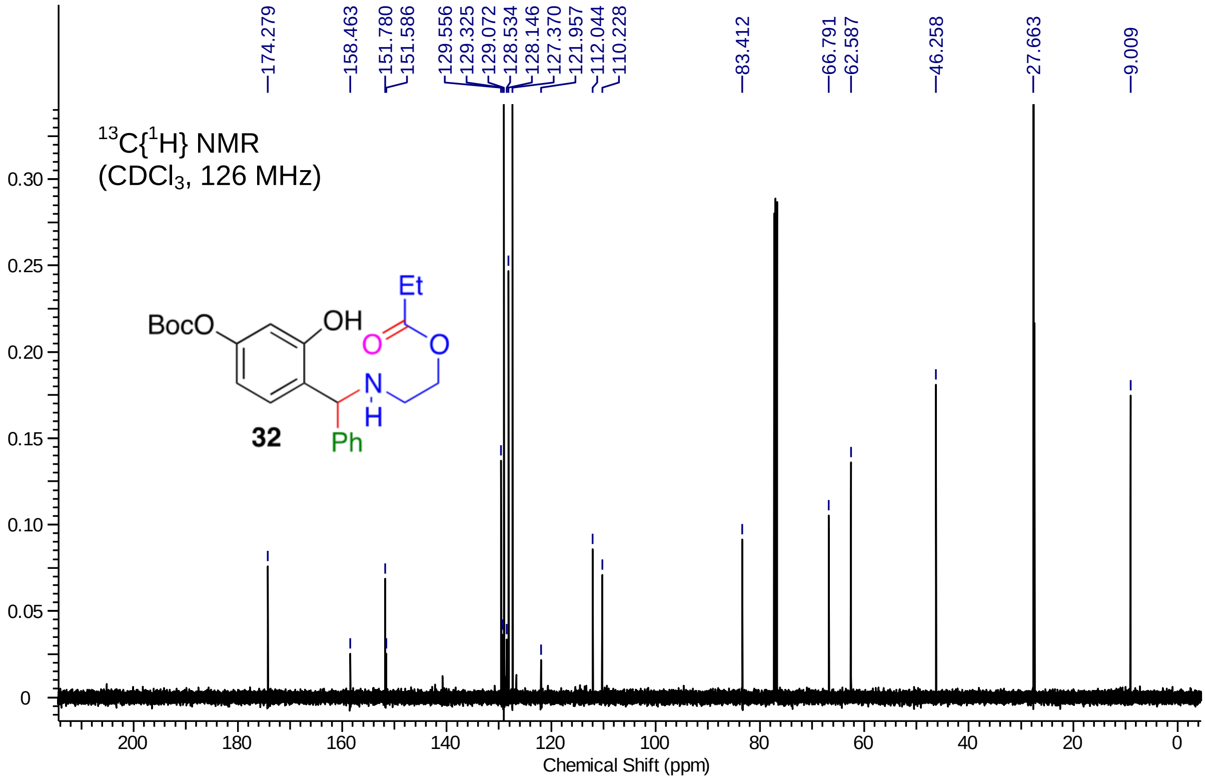

174.279 158.463 151.780 151.586 129.556 129.325 129.072 128.534 128.146 127.370 121.957 112.044 110.228 83.412 66.791 62.587 46.258 27.663 9.009
13C{1H} NMR
(CDCl3, 126 MHz)
BocO
OH
Et
O
O
N
H
32
Ph
Chemical Shift (ppm)

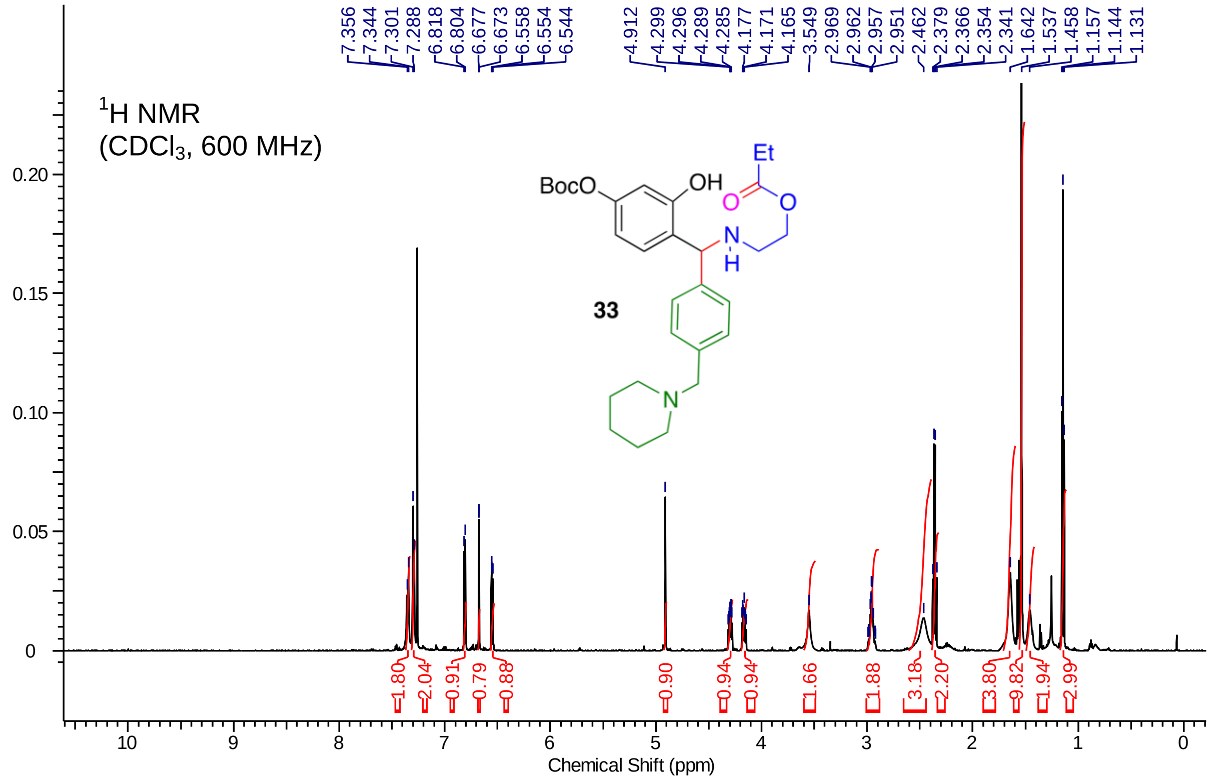
7.356
7.344
7.301
7.288
6.818
6.804
6.677
6.673
6.558
6.554
6.544
4.912
4.299
4.296
4.289
4.285
4.177
4.171
4.165
3.549
2.969
2.962
2.957
2.951
2.462
2.379
2.366
2.354
2.341
1.642
1.537
1.458
1.157
1.144
1.131
^{1}H NMR
($CDCl_3$, 600 MHz)
BocO
OH
Et
O
O
N
H
33
N
1.80
2.04
0.91
0.79
0.88
0.90
0.94
0.94
1.66
1.88
3.18
2.20
3.80
9.82
1.94
2.99
0.20
0.15
0.10
0.05
0
10
9
8
7
6
5
4
3
2
1
0
Chemical Shift (ppm)

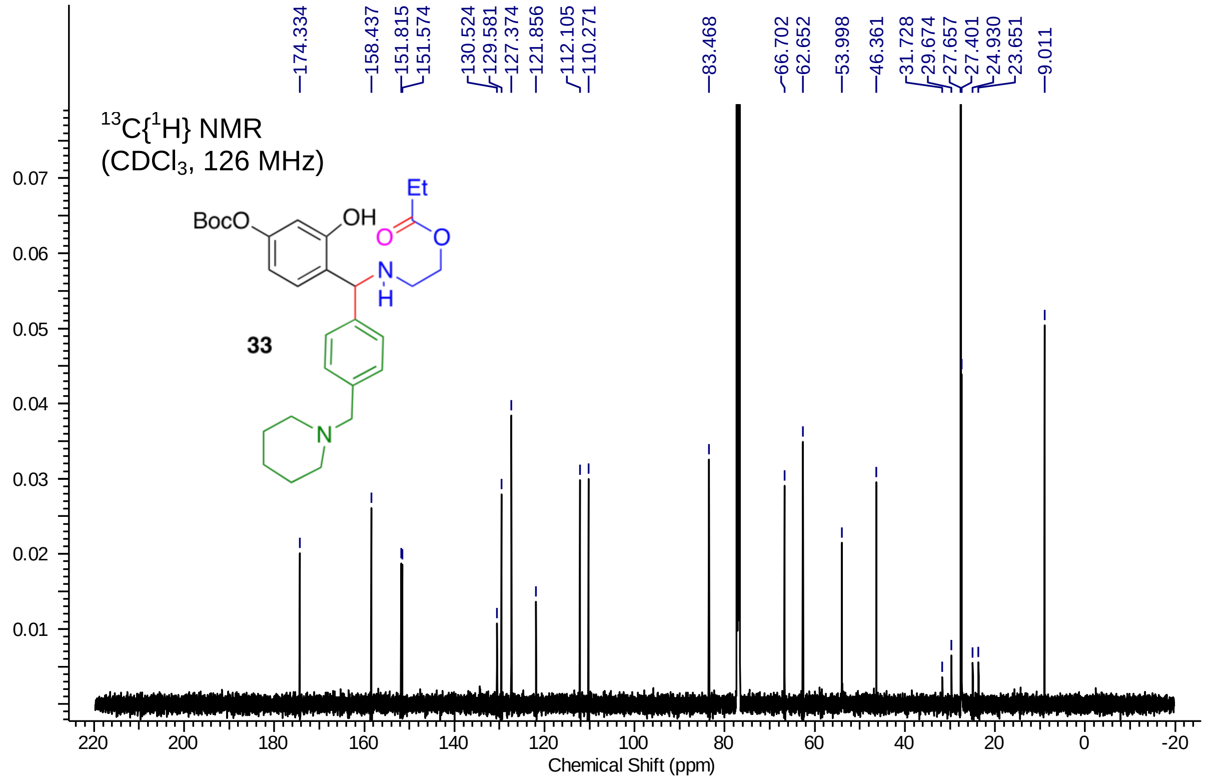
174.334
158.437
151.815
151.574
130.524
129.581
127.374
121.856
112.105
110.271
83.468
66.702
62.652
53.998
46.361
31.728
29.674
27.657
27.401
24.930
23.651
9.011
^{13}C{^{1}H} NMR
($CDCl_3$, 126 MHz)
BocO
OH
Et
O
O
N
H
33
N
0.07
0.06
0.05
0.04
0.03
0.02
0.01
220
200
180
160
140
120
100
80
60
40
20
0
-20
Chemical Shift (ppm)

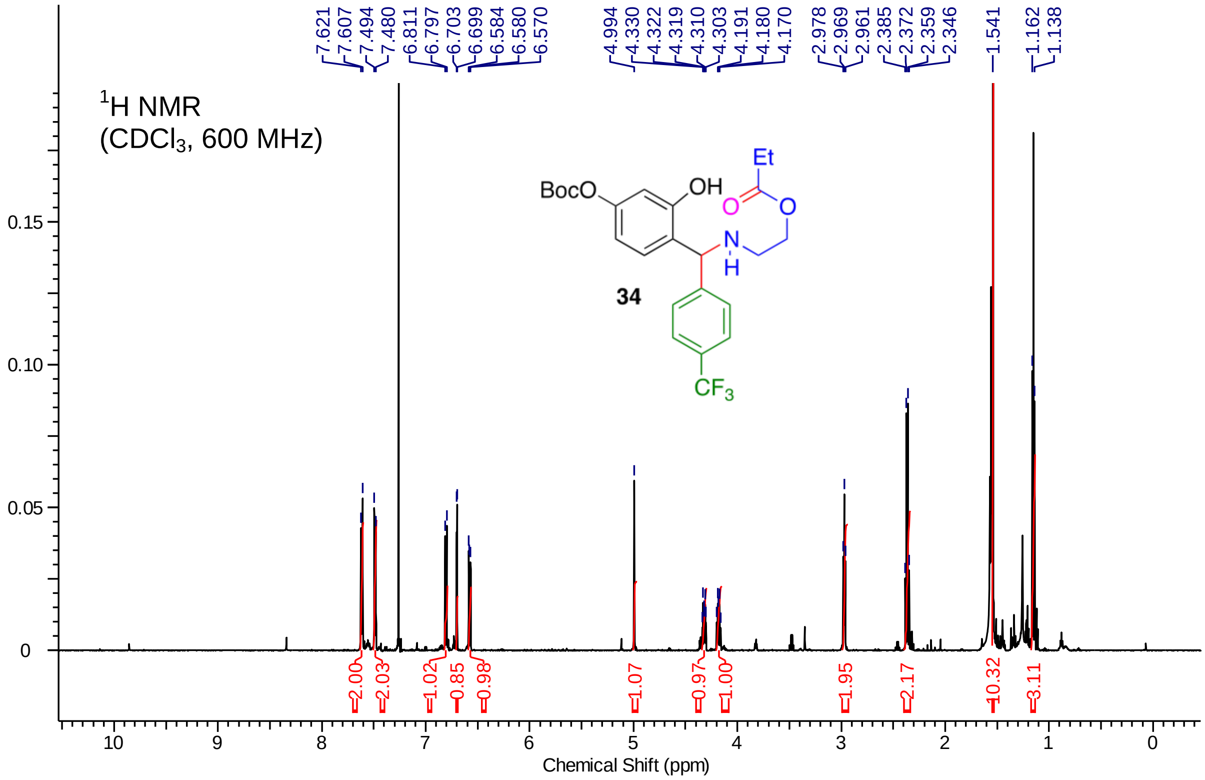
7.621
7.607
7.494
7.480
6.811
6.797
6.703
6.699
6.584
6.580
6.570
4.994
4.330
4.322
4.319
4.310
4.303
4.191
4.180
4.170
2.978
2.969
2.961
2.385
2.372
2.359
2.346
1.541
1.162
1.138
1H NMR
(CDCl3, 600 MHz)
BocO
OH
Et
O
O
N
H
34
CF3
2.00
2.03
1.02
0.85
0.98
1.07
0.97
1.00
1.95
2.17
10.32
3.11
Chemical Shift (ppm)

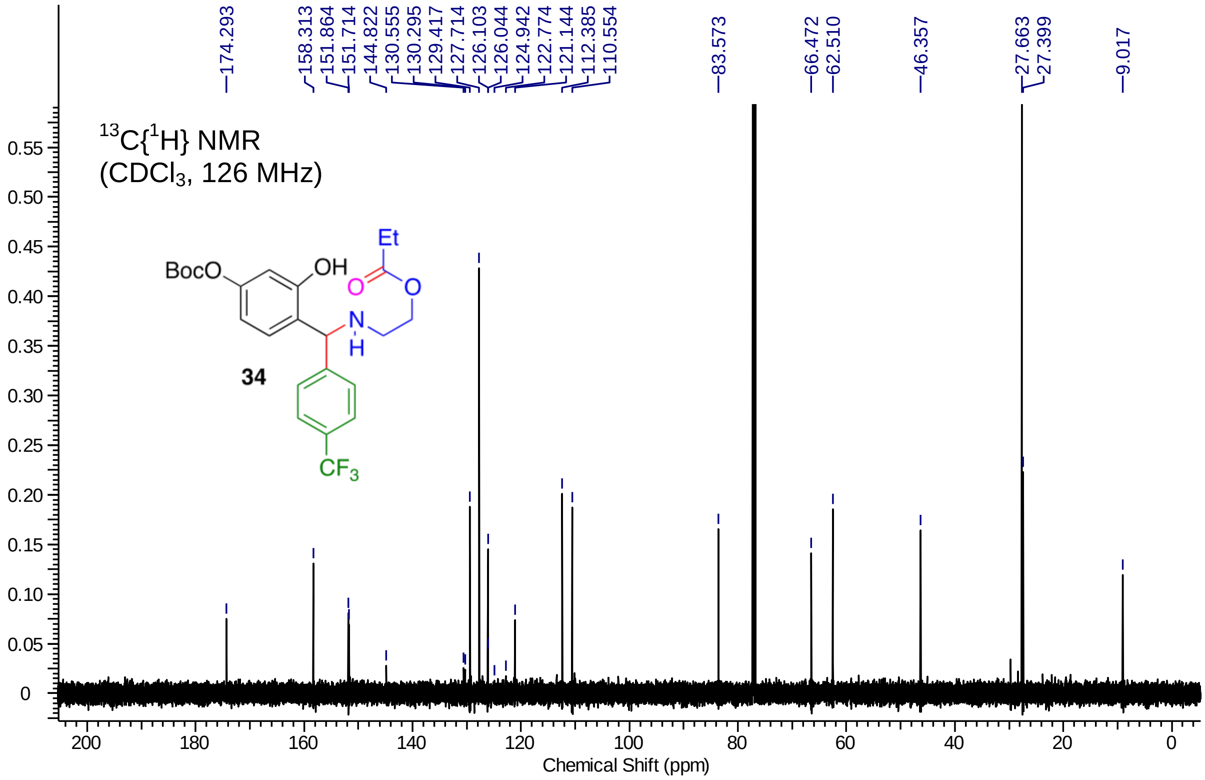
174.293
158.313
151.864
151.714
144.822
130.555
130.295
129.417
127.714
126.103
126.044
124.942
122.774
121.144
112.385
110.554
83.573
66.472
62.510
46.357
27.663
27.399
9.017
13C{1H} NMR
(CDCl3, 126 MHz)
BocO
OH
Et
O
O
N
H
34
CF3
Chemical Shift (ppm)

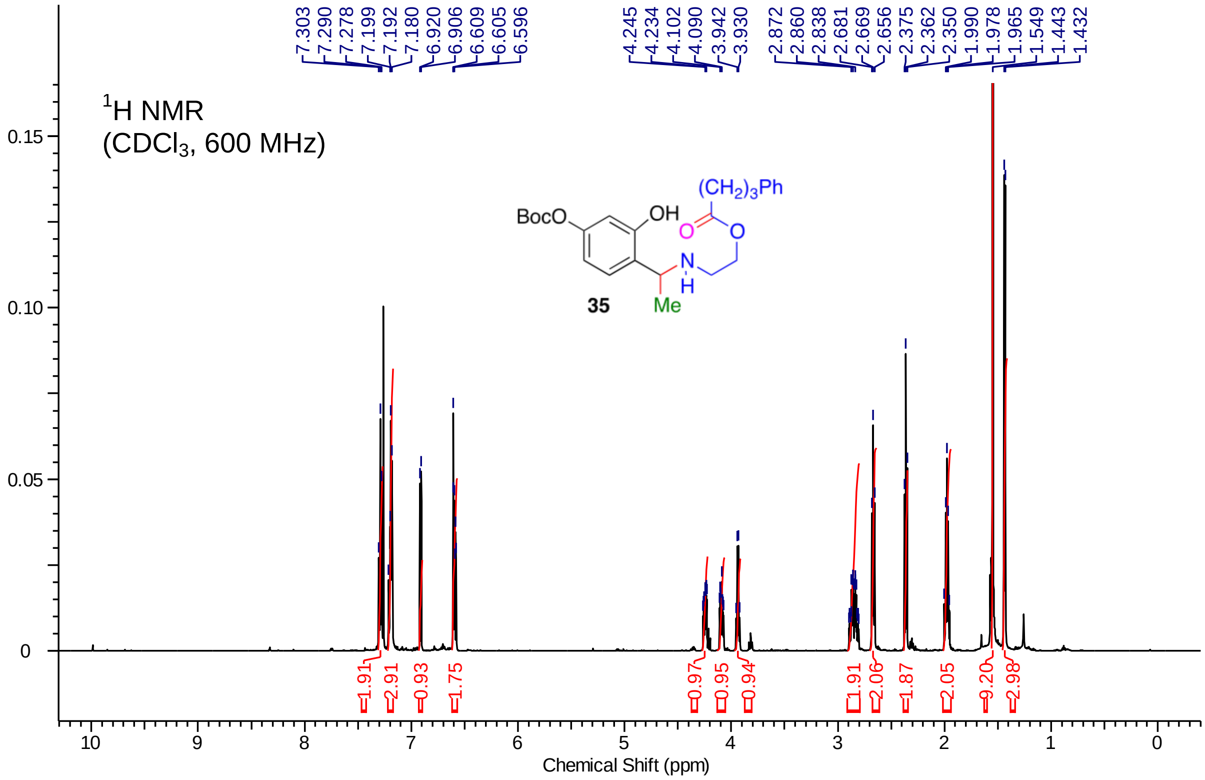

7.303 7.290 7.278 7.199 7.192 7.180 6.920 6.906 6.609 6.605 6.596
4.245 4.234 4.102 4.090 3.942 3.930
2.872 2.860 2.838 2.681 2.669 2.656 2.375 2.362 2.350 1.990 1.978 1.965 1.549 1.443 1.432
1H NMR
(CDCl3, 600 MHz)
BocO
OH
(CH2)3Ph
O
O
N
H
35
Me
1.91 2.91 0.93 1.75 0.97 0.95 0.94 1.91 2.06 1.87 2.05 9.20 2.98
Chemical Shift (ppm)

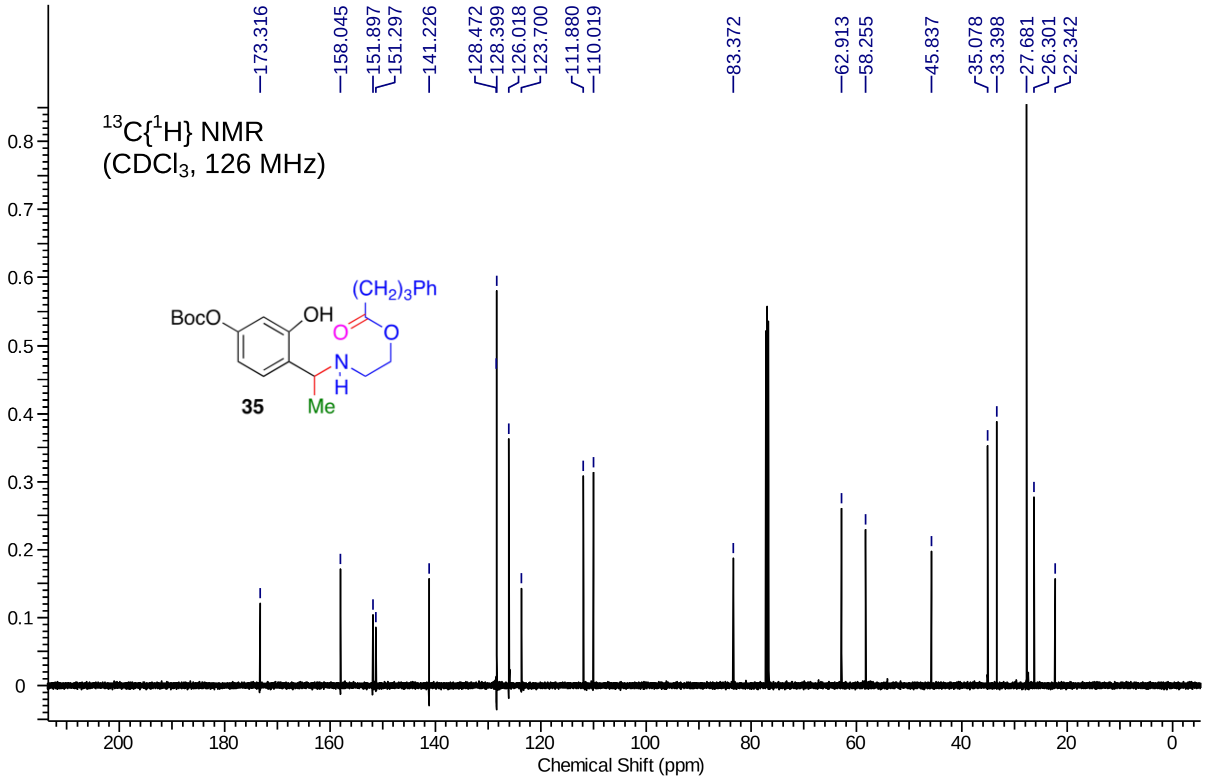

173.316
158.045 151.897 151.297
141.226
128.472 128.399 126.018 123.700
111.880 110.019
83.372
62.913 58.255
45.837
35.078 33.398
27.681 26.301 22.342
13C{1H} NMR
(CDCl3, 126 MHz)
BocO
OH
(CH2)3Ph
O
O
N
H
35
Me
Chemical Shift (ppm)

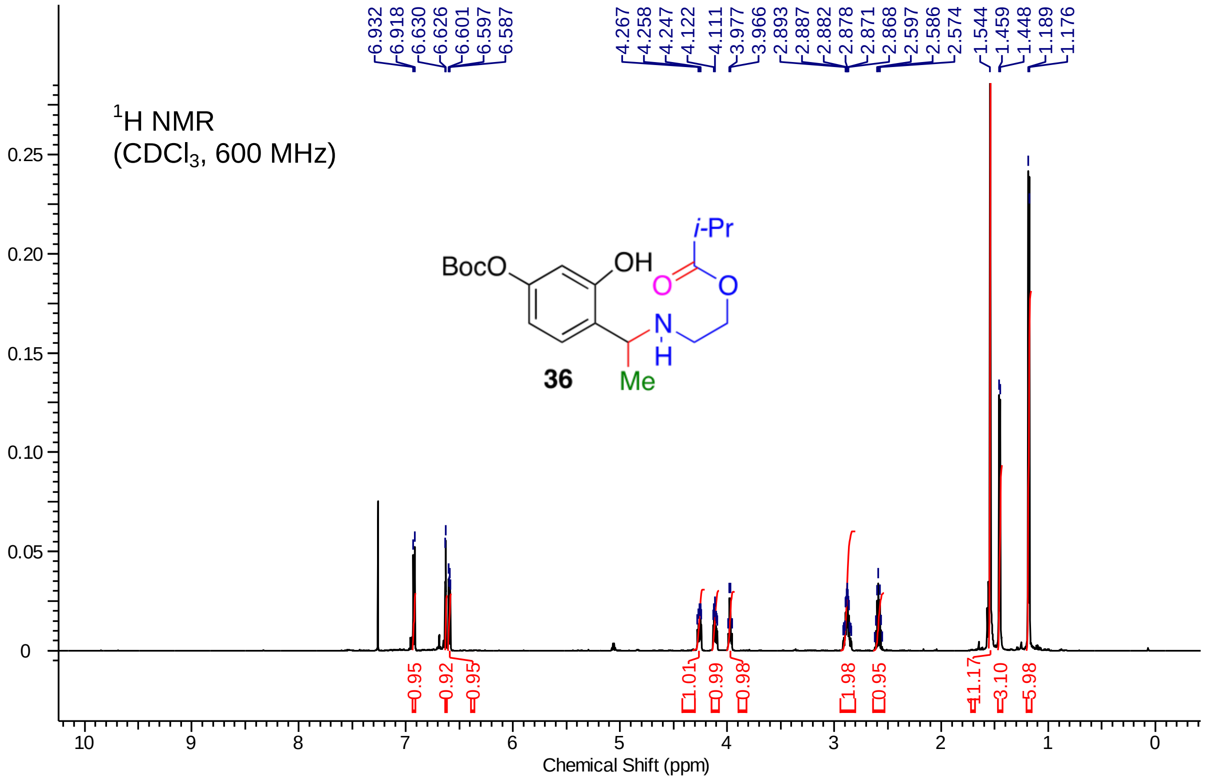
6.932
6.918
6.630
6.626
6.601
6.597
6.587
4.267
4.258
4.247
4.122
4.111
3.977
3.966
2.893
2.887
2.882
2.878
2.871
2.868
2.597
2.586
2.574
1.544
1.459
1.448
1.189
1.176
1H NMR
(CDCl3, 600 MHz)
BocO
OH
i-Pr
O
O
N
H
36
Me
0.95
0.92
0.95
1.01
0.99
0.98
1.98
0.95
1.17
3.10
5.98
Chemical Shift (ppm)

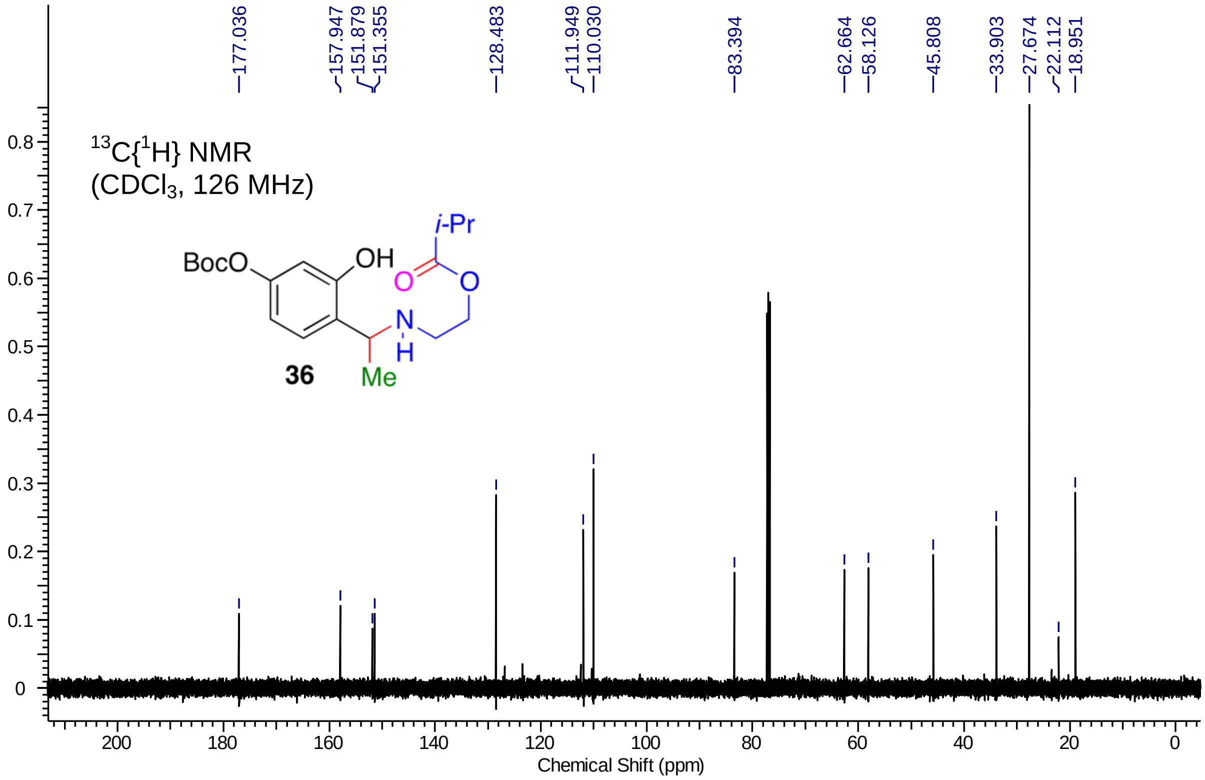
177.036
157.947
151.879
151.355
128.483
111.949
110.030
83.394
62.664
58.126
45.808
33.903
27.674
22.112
18.951
13C{1H} NMR
(CDCl3, 126 MHz)
BocO
OH
i-Pr
O
O
N
H
36
Me
Chemical Shift (ppm)

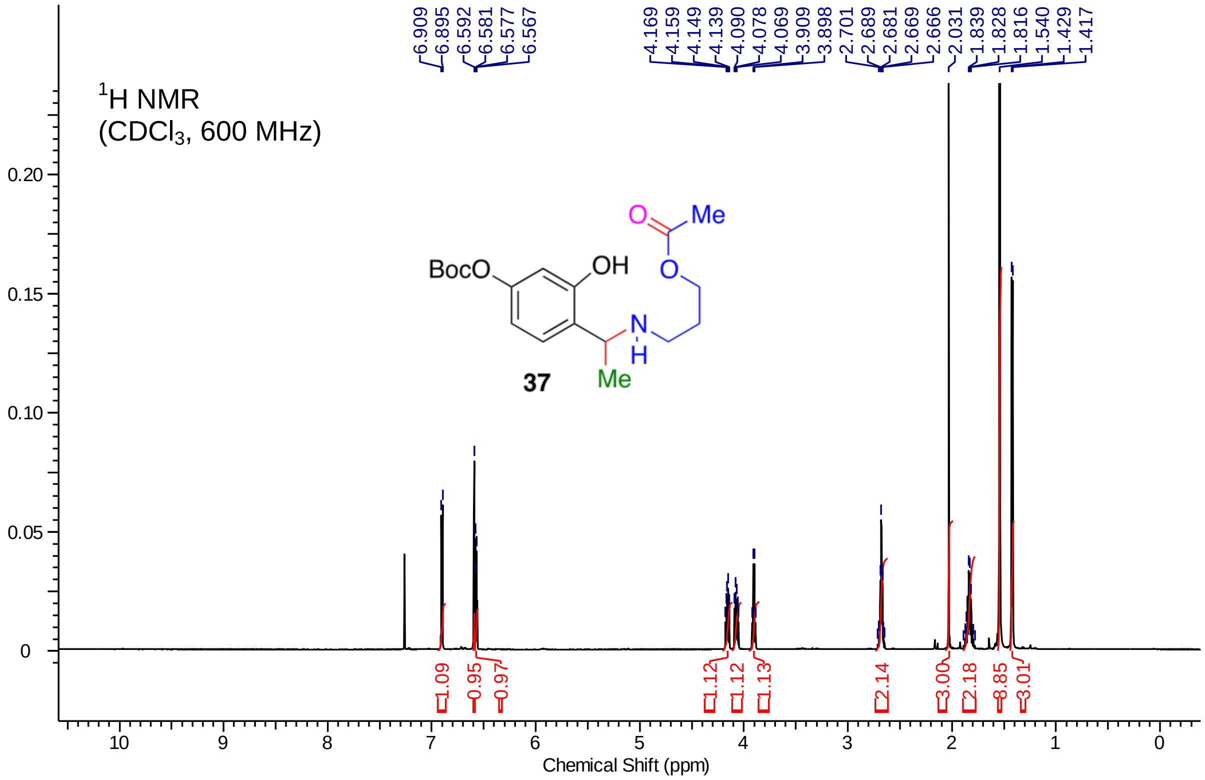

6.909
6.895
6.592
6.581
6.577
6.567
4.169
4.159
4.149
4.139
4.090
4.078
4.069
3.909
3.898
2.701
2.689
2.681
2.669
2.666
2.031
1.839
1.828
1.816
1.540
1.429
1.417
1H NMR
(CDCl3, 600 MHz)
BocO
OH
O
Me
O
N
H
37
Me
1.09
0.95
0.97
1.12
1.12
1.13
2.14
3.00
2.18
8.85
3.01
Chemical Shift (ppm)

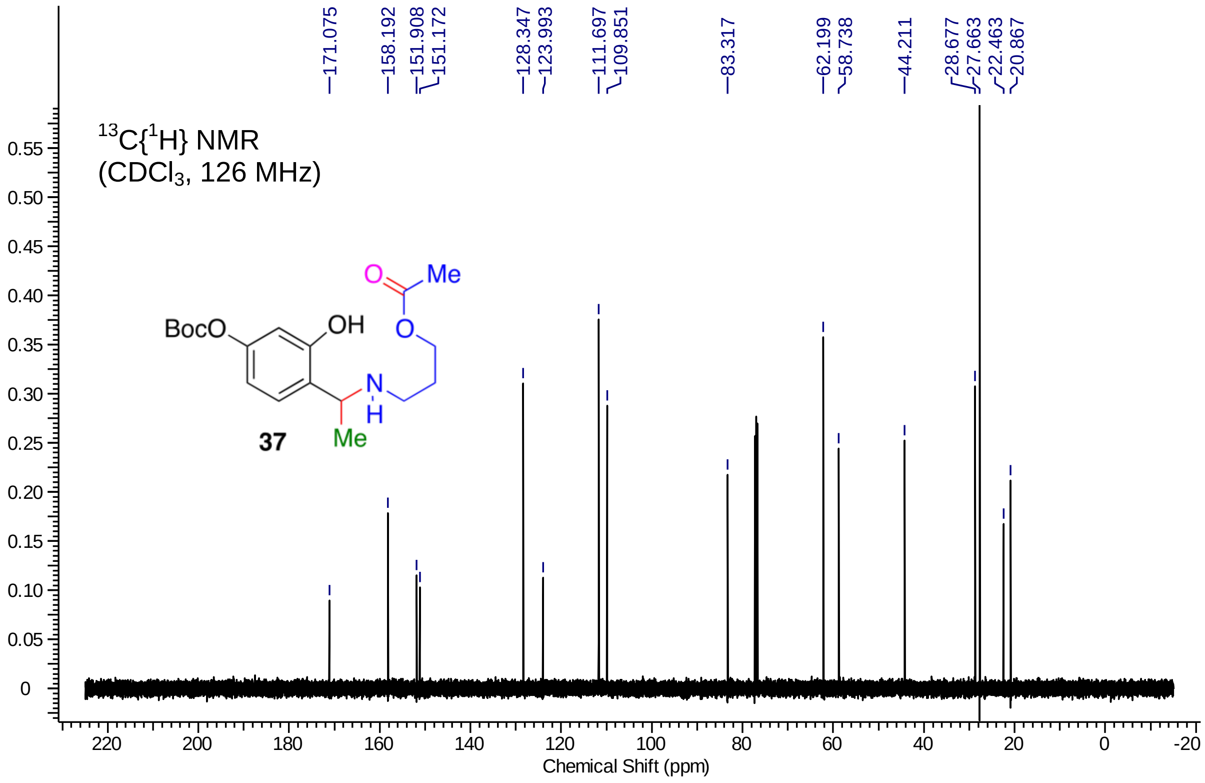

171.075
158.192
151.908
151.172
128.347
123.993
111.697
109.851
83.317
62.199
58.738
44.211
28.677
27.663
22.463
20.867
13C{1H} NMR
(CDCl3, 126 MHz)
BocO
OH
O
Me
O
N
H
37
Me
Chemical Shift (ppm)

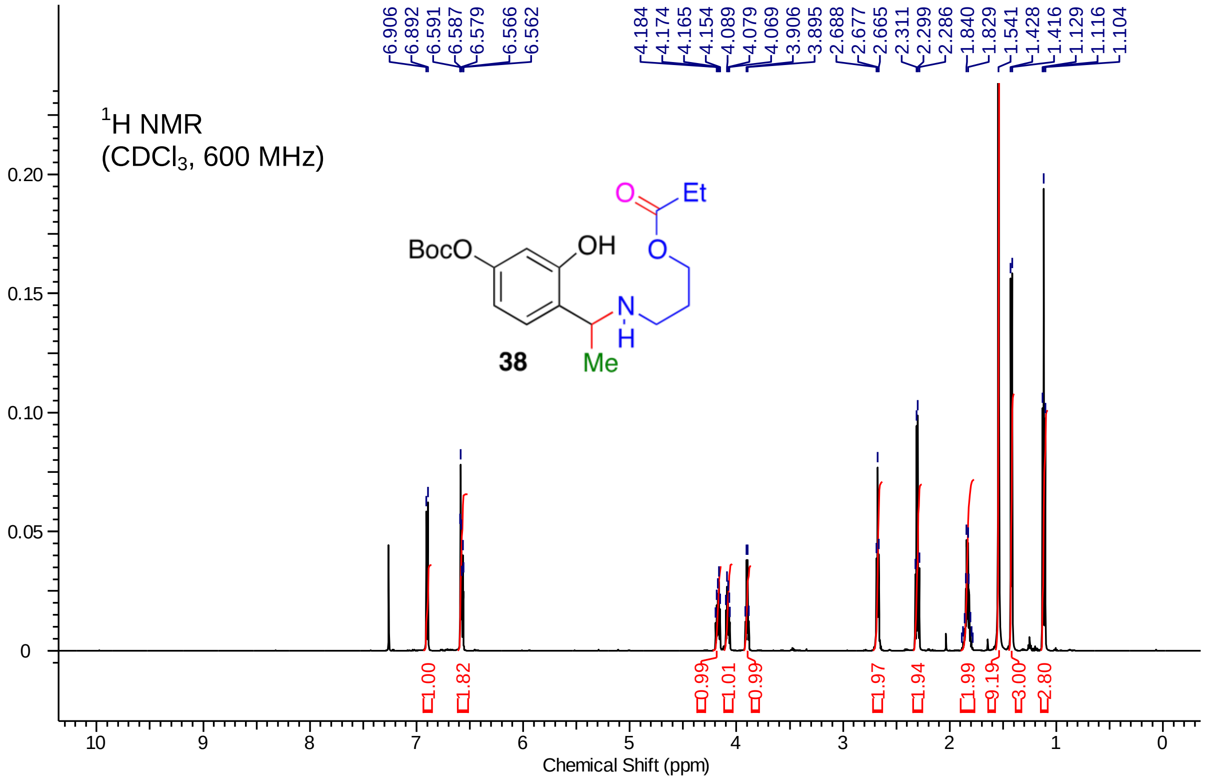

6.906
6.892
6.591
6.587
6.579
6.566
6.562
4.184
4.174
4.165
4.154
4.089
4.079
4.069
3.906
3.895
2.688
2.677
2.665
2.311
2.299
2.286
1.840
1.829
1.541
1.428
1.416
1.129
1.116
1.104
^{1}H NMR
($CDCl_3$, 600 MHz)
BocO
OH
O
Et
O
N
H
38
Me
1.00
1.82
0.99
1.01
0.99
1.97
1.94
1.99
9.19
3.00
2.80
Chemical Shift (ppm)

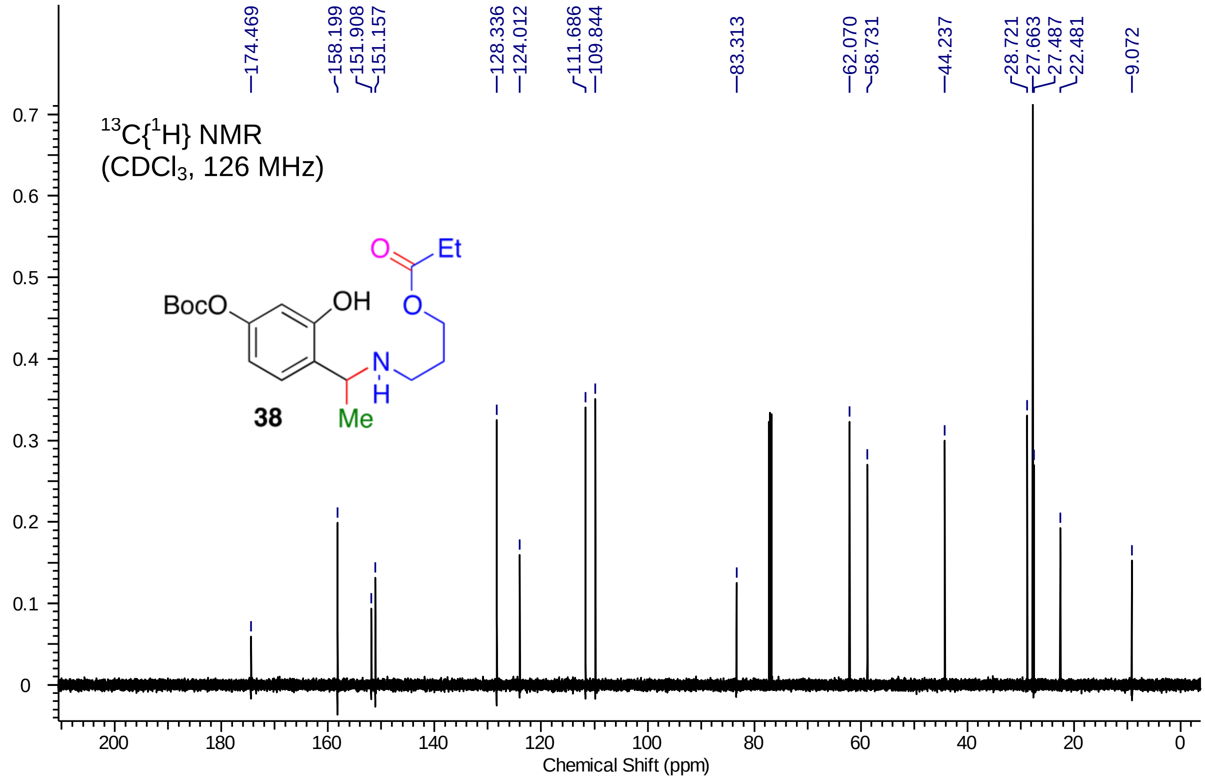

174.469
158.199
151.908
151.157
128.336
124.012
111.686
109.844
83.313
62.070
58.731
44.237
28.721
27.663
27.487
22.481
9.072
^{13}C{^{1}H} NMR
($CDCl_3$, 126 MHz)
BocO
OH
O
Et
O
N
H
38
Me
Chemical Shift (ppm)

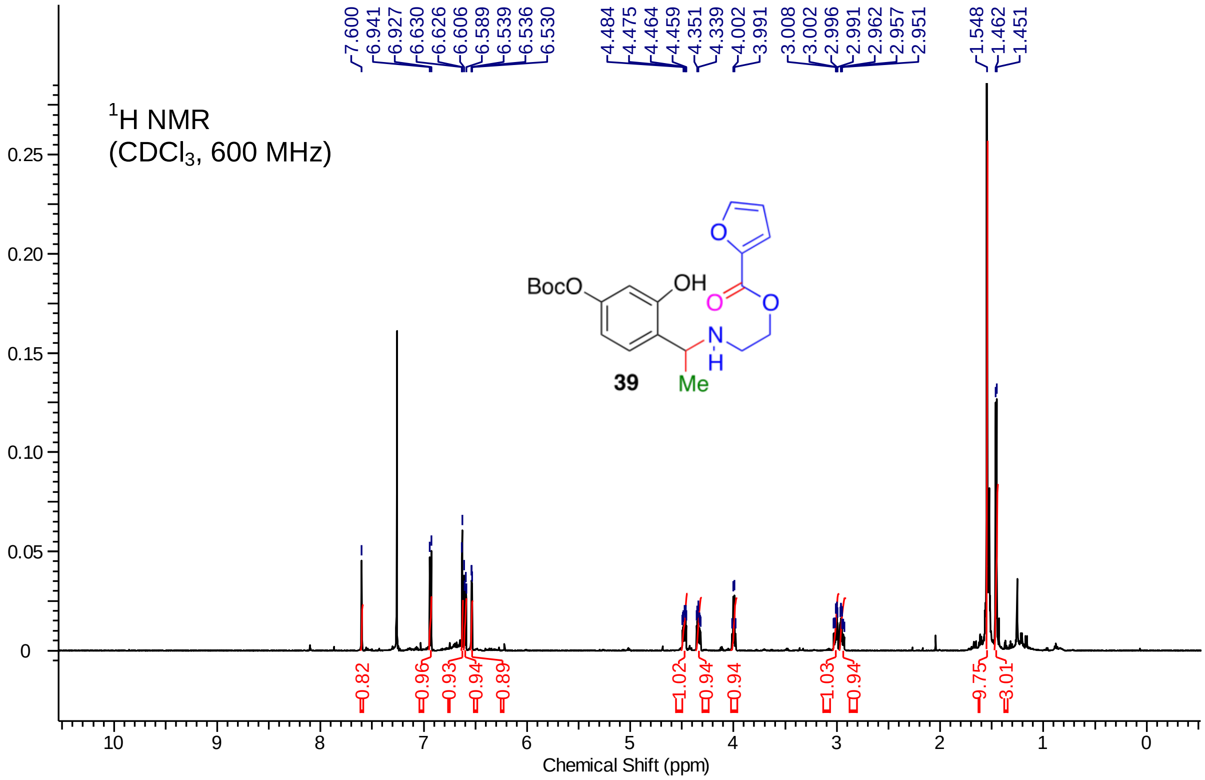

7.600
6.941
6.927
6.630
6.626
6.606
6.589
6.539
6.536
6.530
4.484
4.475
4.464
4.459
4.351
4.339
4.002
3.991
3.008
3.002
2.996
2.991
2.962
2.957
2.951
1.548
1.462
1.451
1H NMR
(CDCl3, 600 MHz)
BocO
OH
O
O
O
N
H
39
Me
0.82
0.96
0.93
0.94
0.89
1.02
0.94
0.94
1.03
0.94
9.75
3.01
Chemical Shift (ppm)

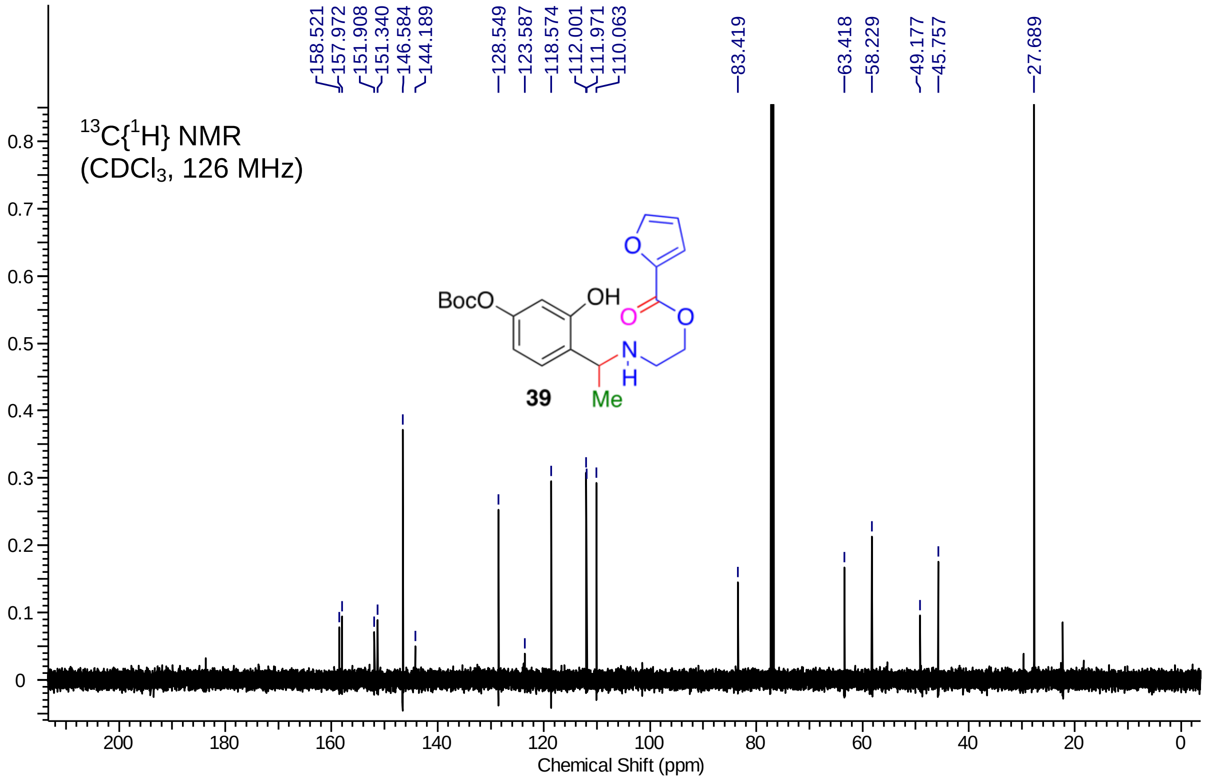

158.521
157.972
151.908
151.340
146.584
144.189
128.549
123.587
118.574
112.001
111.971
110.063
83.419
63.418
58.229
49.177
45.757
27.689
13C{1H} NMR
(CDCl3, 126 MHz)
BocO
OH
O
O
O
N
H
39
Me
Chemical Shift (ppm)

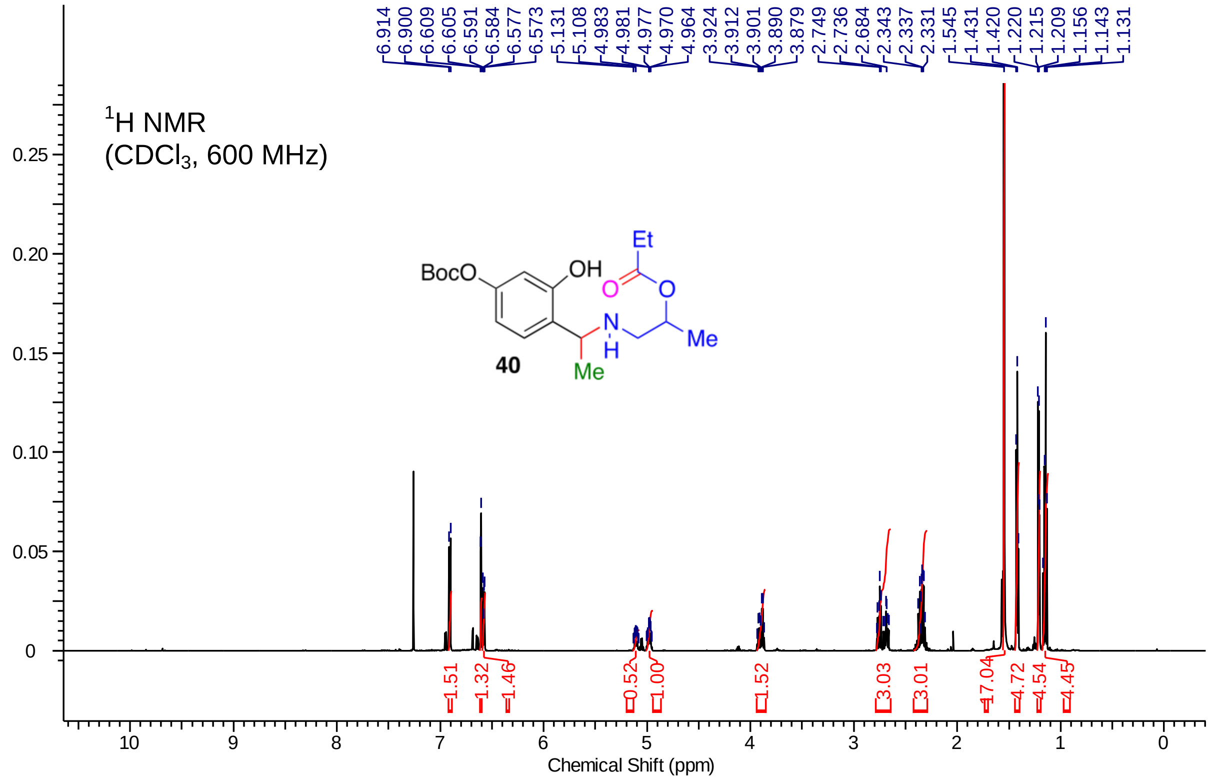
6.914
6.900
6.609
6.605
6.591
6.584
6.577
6.573
5.131
5.108
4.983
4.981
4.977
4.970
4.964
3.924
3.912
3.901
3.890
3.879
2.749
2.736
2.684
2.343
2.337
2.331
1.545
1.431
1.420
1.220
1.215
1.209
1.156
1.143
1.131
1H NMR
(CDCl3, 600 MHz)
BocO
OH
Et
O
O
N
H
Me
40
Me
1.51
1.32
1.46
0.52
1.00
1.52
3.03
3.01
17.04
4.72
4.54
4.45
Chemical Shift (ppm)

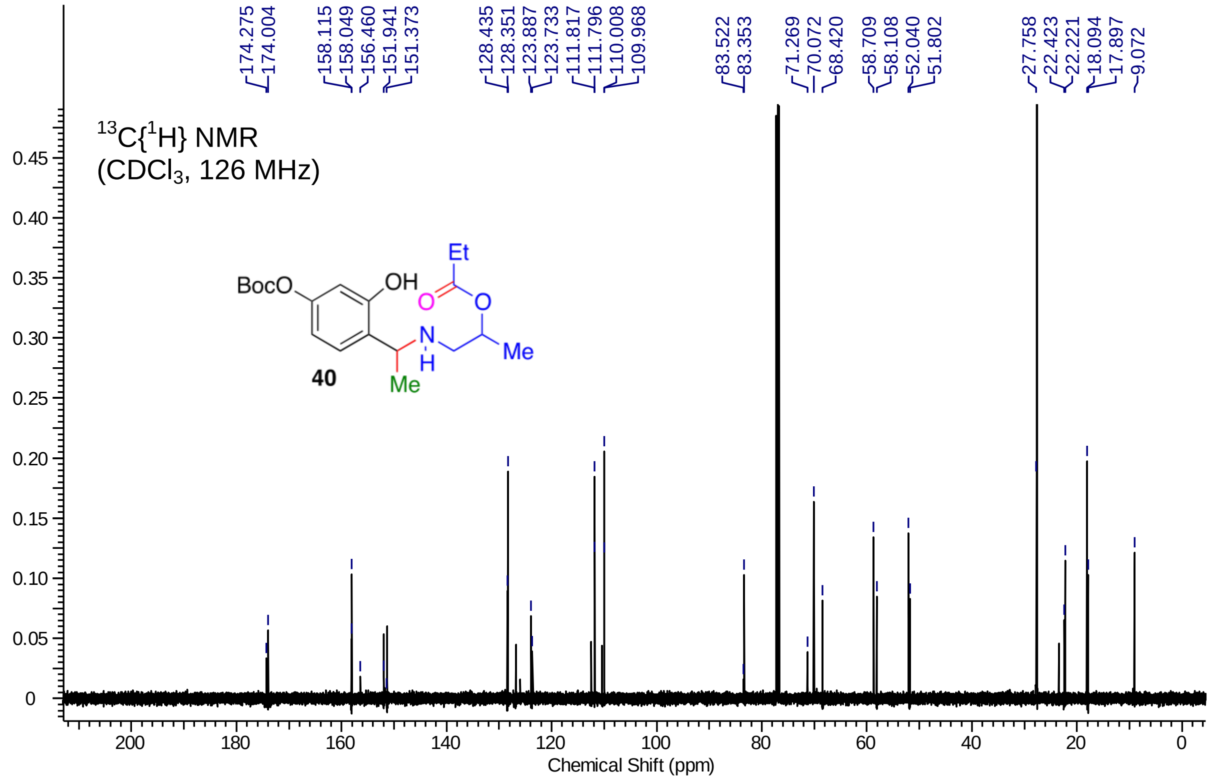
174.275
174.004
158.115
158.049
156.460
151.941
151.373
128.435
128.351
123.887
123.733
111.817
111.796
110.008
109.968
83.522
83.353
71.269
70.072
68.420
58.709
58.108
52.040
51.802
27.758
22.423
22.221
18.094
17.897
9.072
13C{1H} NMR
(CDCl3, 126 MHz)
BocO
OH
Et
O
O
N
H
Me
40
Me
Chemical Shift (ppm)

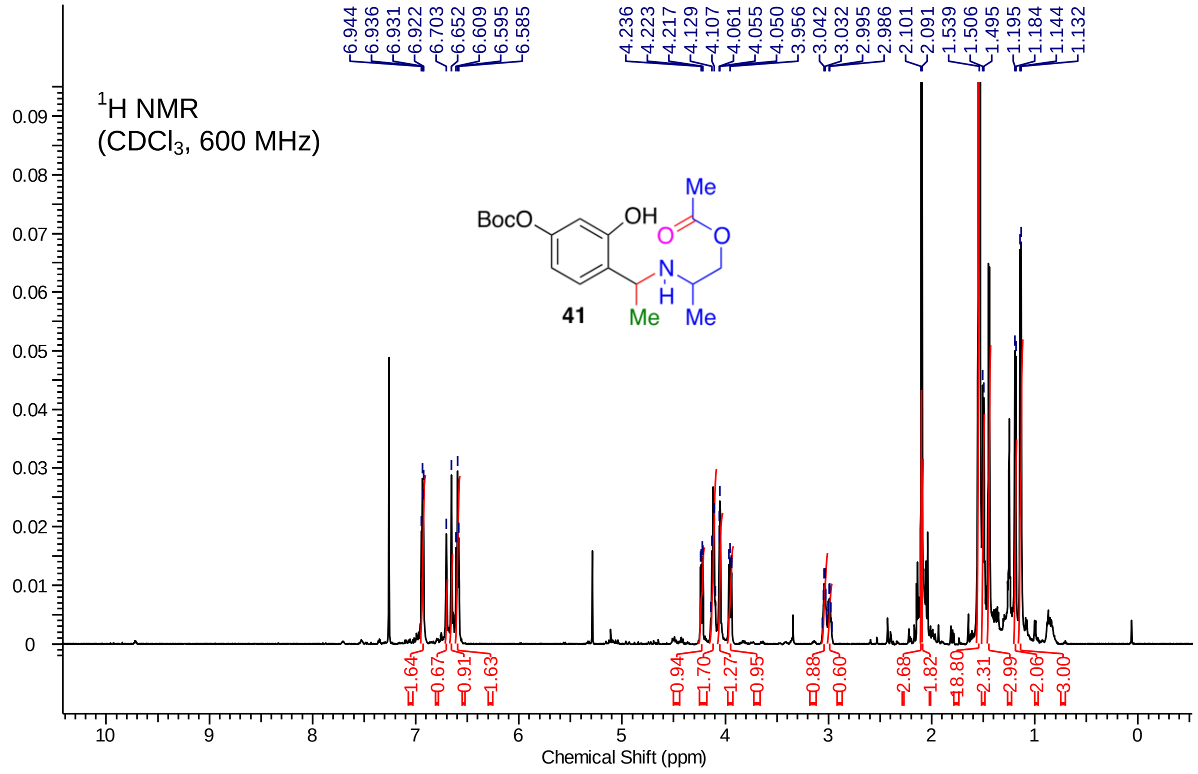
6.944 6.936 6.931 6.922 6.703 6.652 6.609 6.595 6.585
4.236 4.223 4.217 4.129 4.107 4.061 4.055 4.050 3.956 3.042 3.032 2.995 2.986 2.101 2.091 1.539 1.506 1.495 1.195 1.184 1.144 1.132
^{1}H NMR
($CDCl_3$, 600 MHz)
BocO
OH
Me
O
O
N
H
41
Me
Me
1.64 0.67 0.91 1.63 0.94 1.70 1.27 0.95 0.88 0.60 2.68 1.82 18.80 2.31 2.99 2.06 3.00
Chemical Shift (ppm)

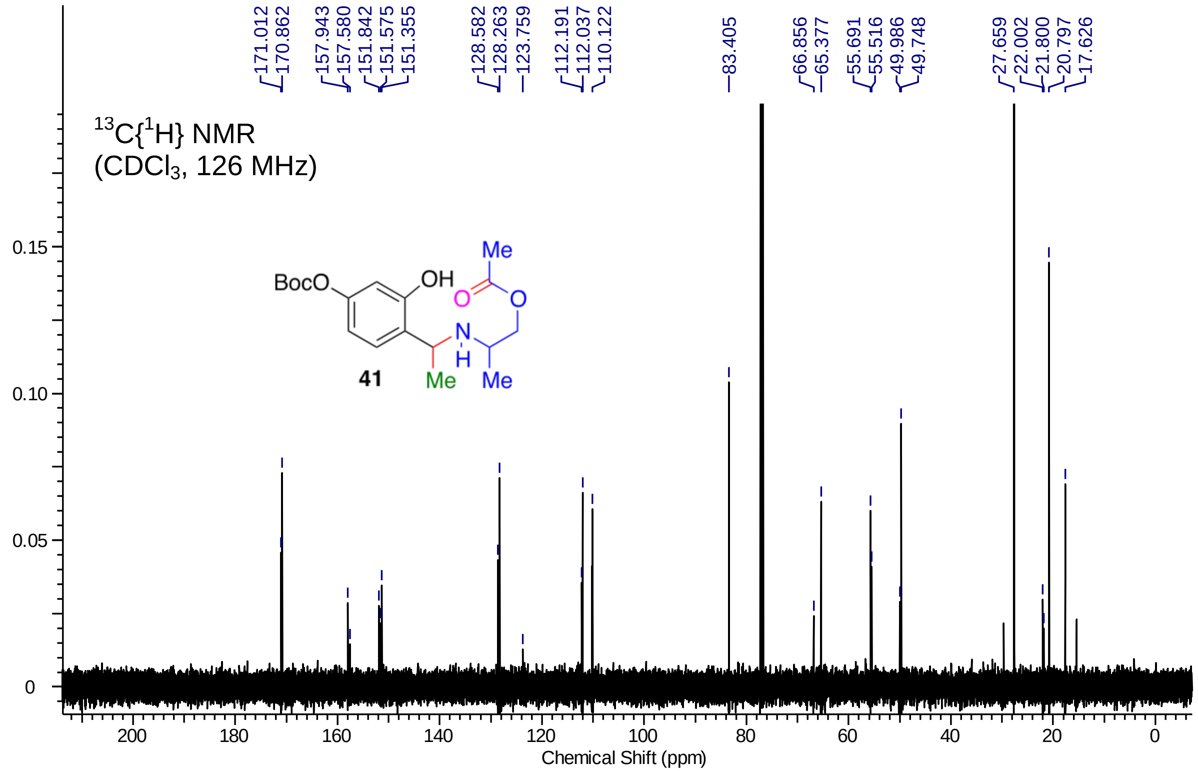
171.012 170.862 157.943 157.580 151.842 151.575 151.355 128.582 128.263 123.759 112.191 112.037 110.122 83.405 66.856 65.377 55.691 55.516 49.986 49.748 27.659 22.002 21.800 20.797 17.626
$^{13}C\{^1H\}$ NMR
($CDCl_3$, 126 MHz)
BocO
OH
Me
O
O
N
H
41
Me
Me
Chemical Shift (ppm)

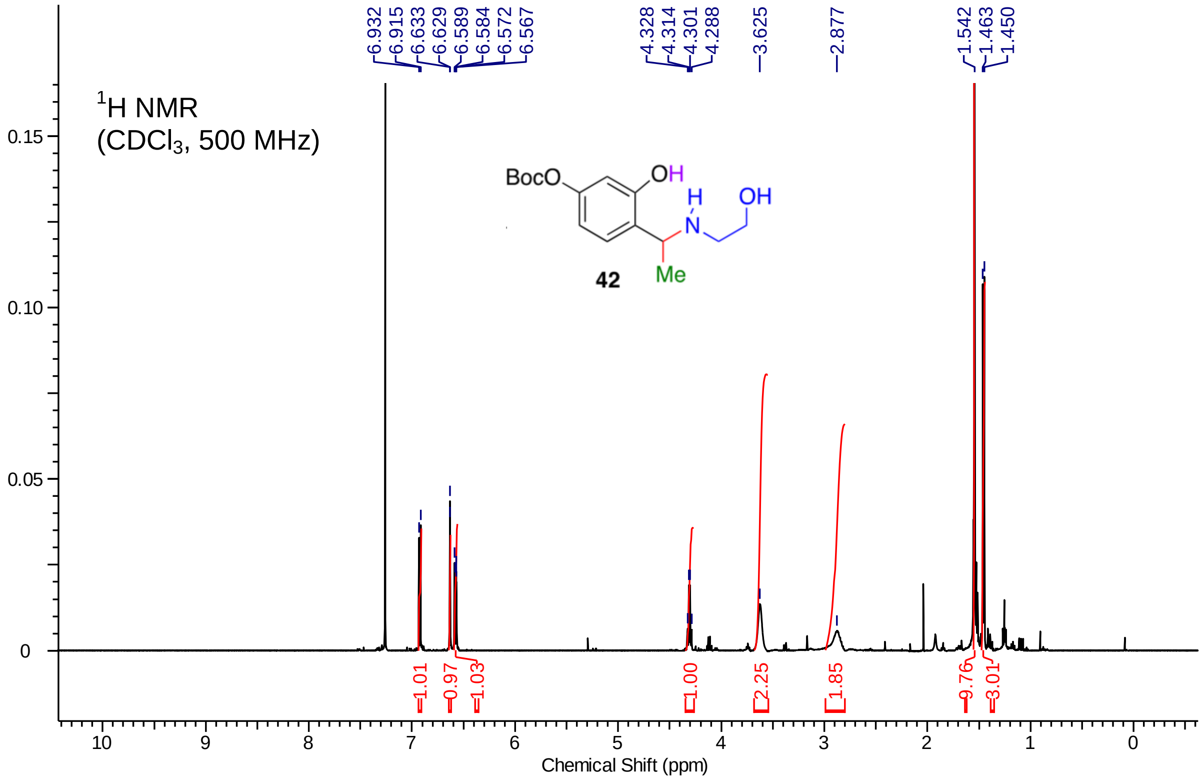

6.932
6.915
6.633
6.629
6.589
6.584
6.572
6.567
4.328
4.314
4.301
4.288
3.625
2.877
1.542
1.463
1.450
1H NMR
(CDCl3, 500 MHz)
BocO
OH
H
N
OH
Me
42
1.01
0.97
1.03
1.00
2.25
1.85
9.76
3.01
Chemical Shift (ppm)

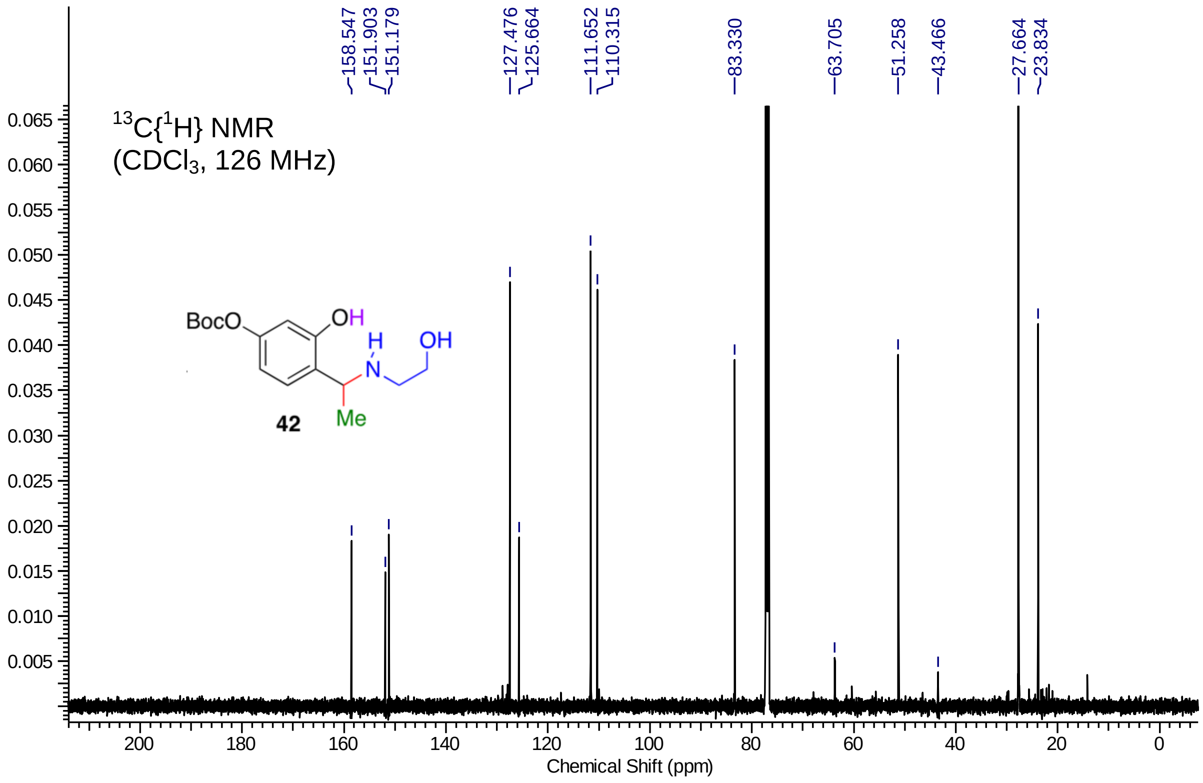

158.547
151.903
151.179
127.476
125.664
111.652
110.315
83.330
63.705
51.258
43.466
27.664
23.834
13C{1H} NMR
(CDCl3, 126 MHz)
BocO
OH
H
N
OH
Me
42
Chemical Shift (ppm)

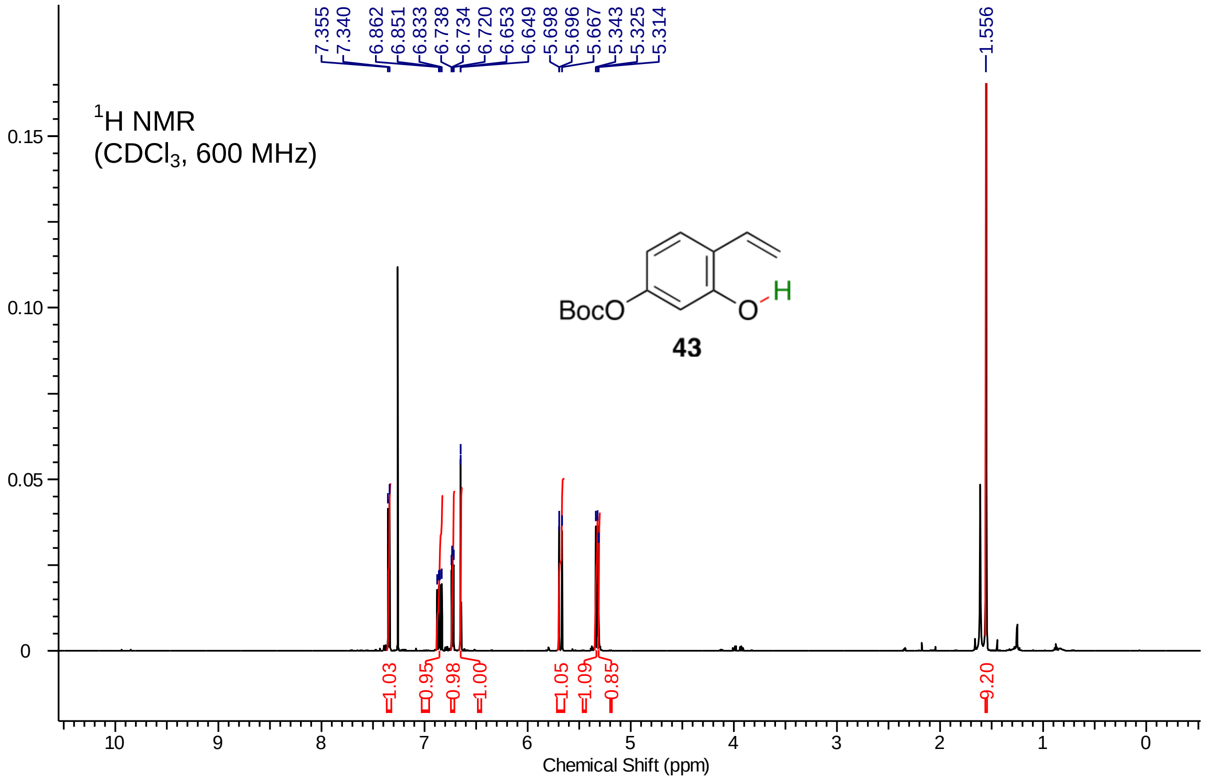

7.355
7.340
6.862
6.851
6.833
6.738
6.734
6.720
6.653
6.649
5.698
5.696
5.667
5.343
5.325
5.314
1.556
^{1}H NMR
($CDCl_3$, 600 MHz)
BocO
O
H
43
1.03
0.95
0.98
1.00
1.05
1.09
0.85
9.20
Chemical Shift (ppm)

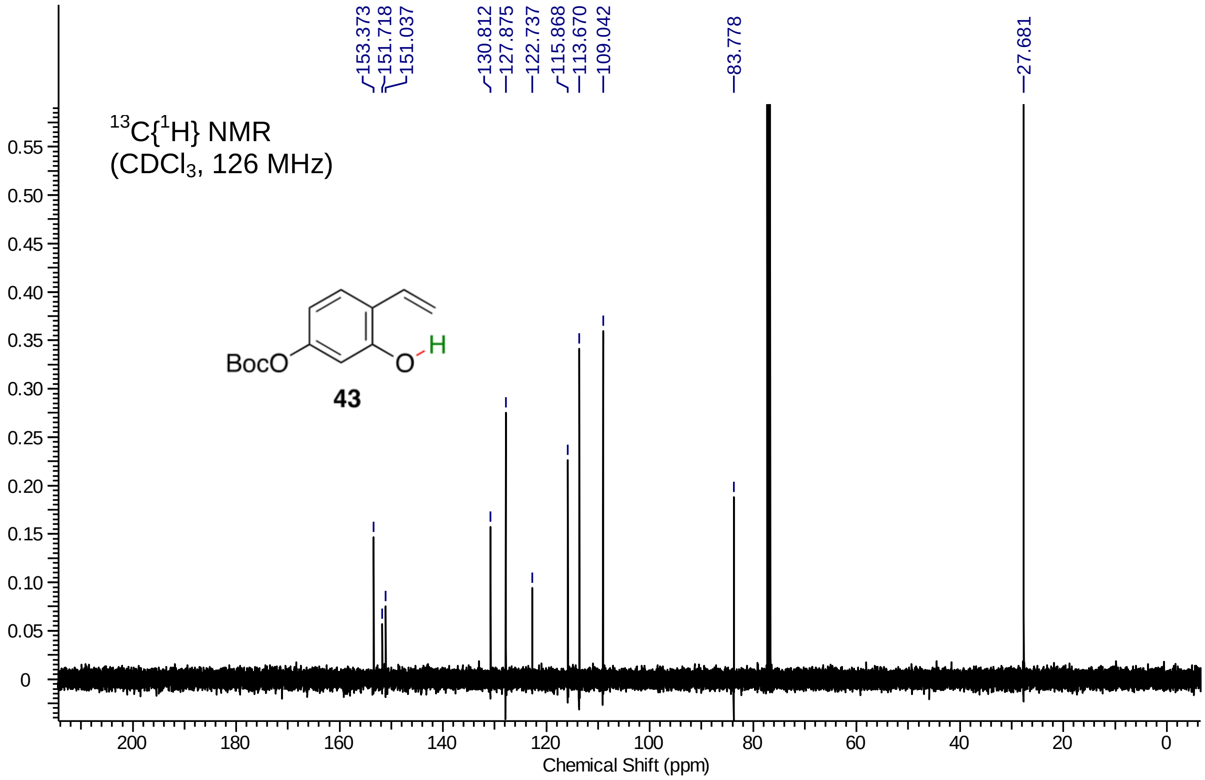

153.373
151.718
151.037
130.812
127.875
122.737
115.868
113.670
109.042
83.778
27.681
^{13}C{^{1}H} NMR
($CDCl_3$, 126 MHz)
BocO
O
H
43
Chemical Shift (ppm)

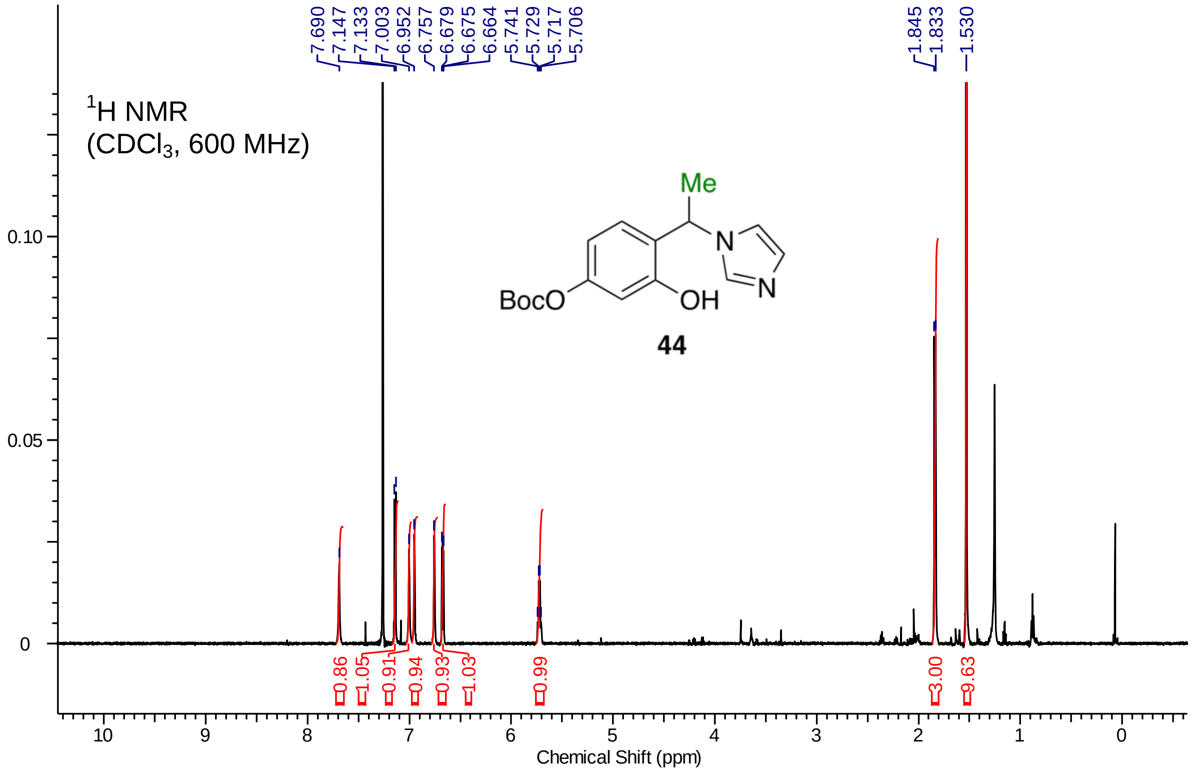

7.690
7.147
7.133
7.003
6.952
6.757
6.679
6.675
6.664
5.741
5.729
5.717
5.706
1.845
1.833
1.530
^{1}H NMR
($CDCl_3$, 600 MHz)
Me
N
N
BocO
OH
44
0.10
0.05
0
0.86
1.05
0.91
0.94
0.93
1.03
0.99
3.00
9.63
10
9
8
7
6
5
4
3
2
1
0
Chemical Shift (ppm)

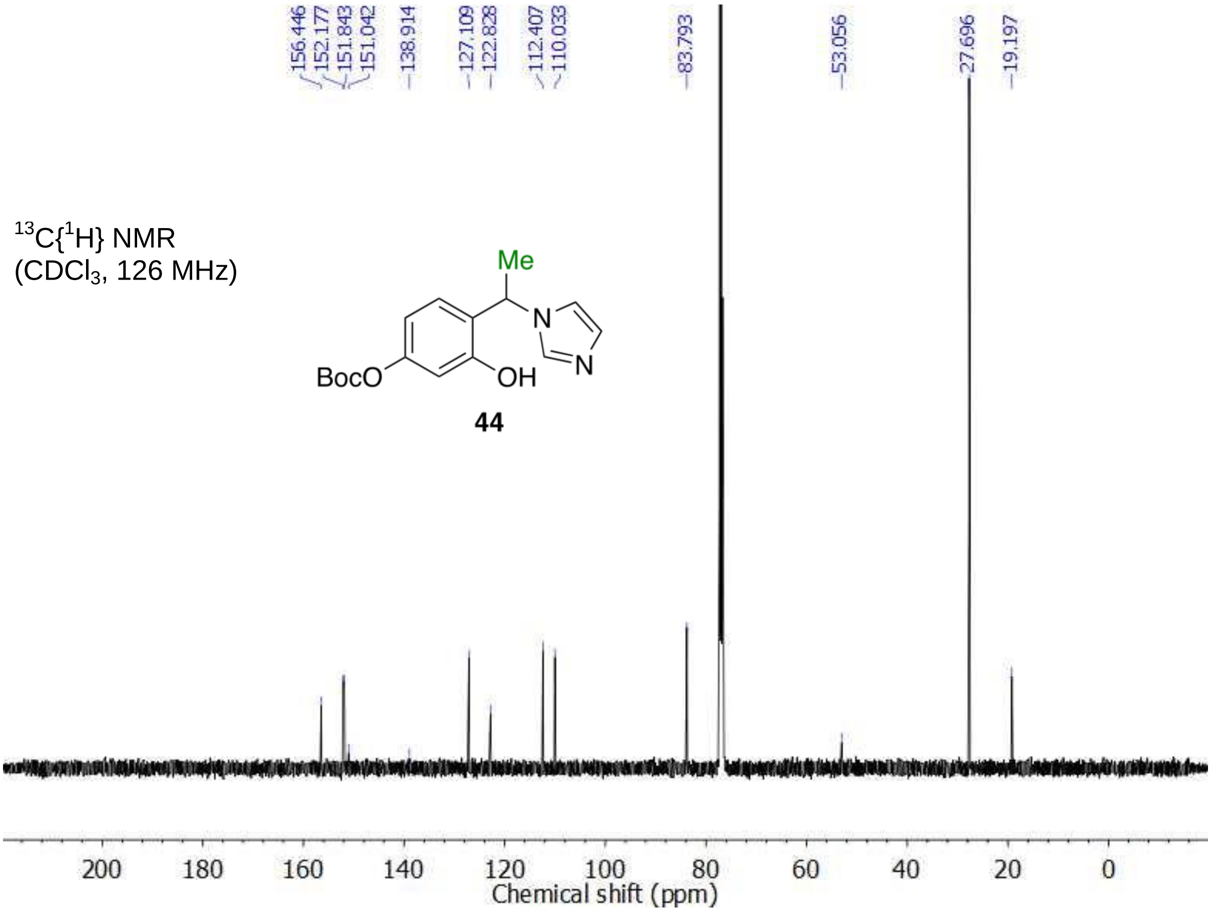

156.446
152.177
151.843
151.042
138.914
127.109
122.828
112.407
110.033
83.793
53.056
27.696
19.197
$^{13}C\{^1H\}$ NMR
($CDCl_3$, 126 MHz)
Me
N
N
BocO
OH
44
200
180
160
140
120
100
80
60
40
20
0
Chemical shift (ppm)

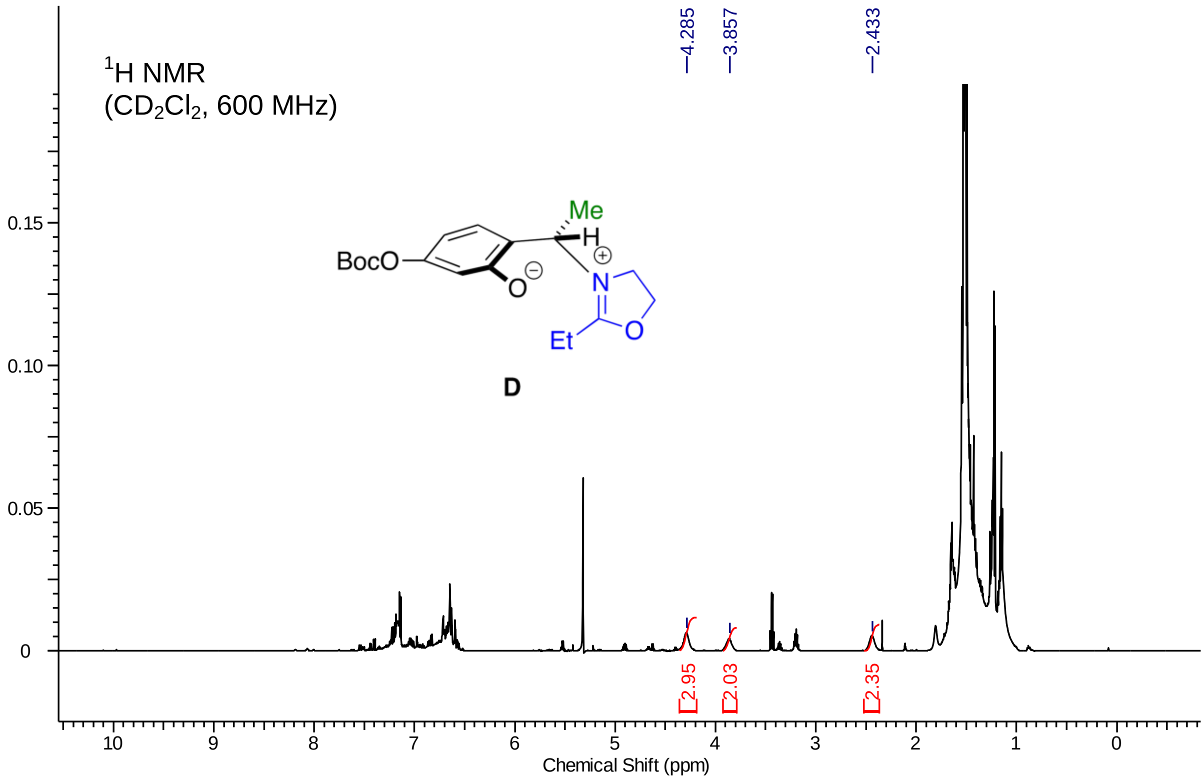

4.285
3.857
2.433
1H NMR
(CD2Cl2, 600 MHz)
Me
H
BocO
O
N
O
Et
D
2.95
2.03
2.35
Chemical Shift (ppm)

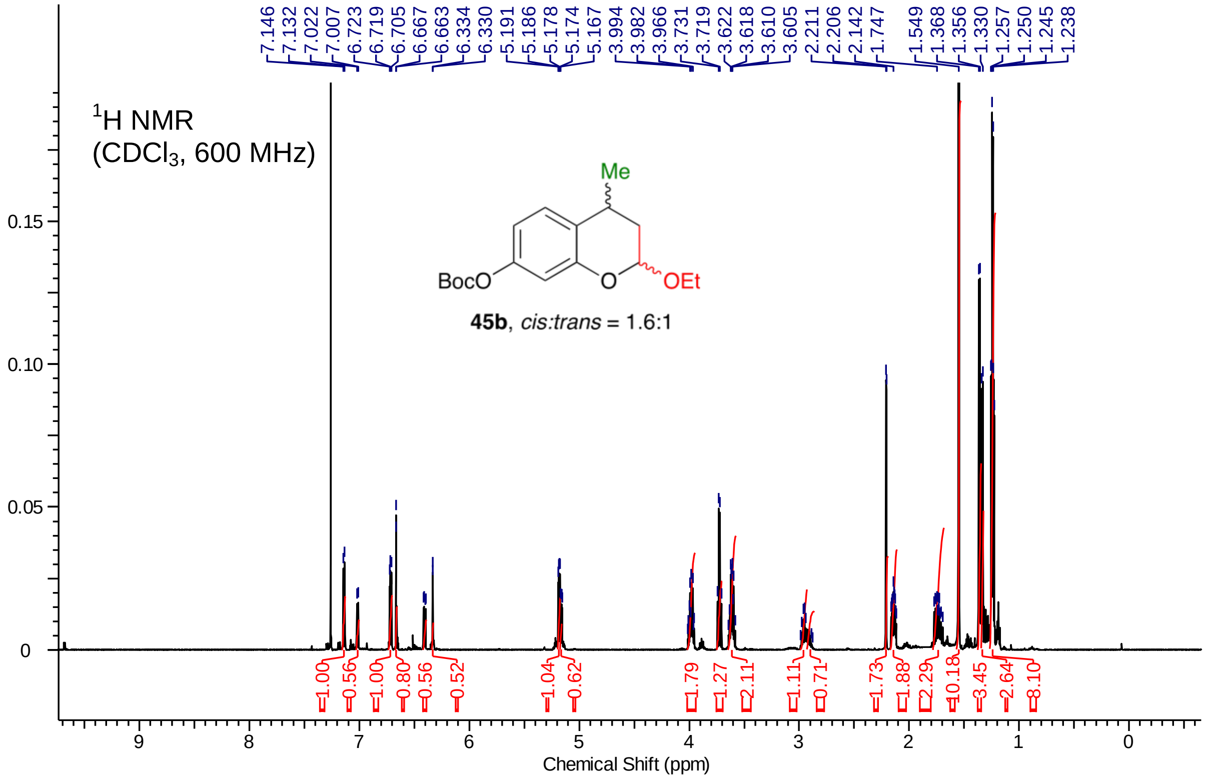

7.146
7.132
7.022
7.007
6.723
6.719
6.705
6.667
6.663
6.334
6.330
5.191
5.186
5.178
5.174
5.167
3.994
3.982
3.966
3.731
3.719
3.622
3.618
3.610
3.605
2.211
2.206
2.142
1.747
1.549
1.368
1.356
1.330
1.257
1.250
1.245
1.238
1H NMR
(CDCl3, 600 MHz)
Me
BocO
O
OEt
45b, cis:trans = 1.6:1
1.00
0.56
1.00
0.80
0.56
0.52
1.04
0.62
1.79
1.27
2.11
1.11
0.71
1.73
1.88
2.29
10.18
3.45
2.64
8.10
Chemical Shift (ppm)

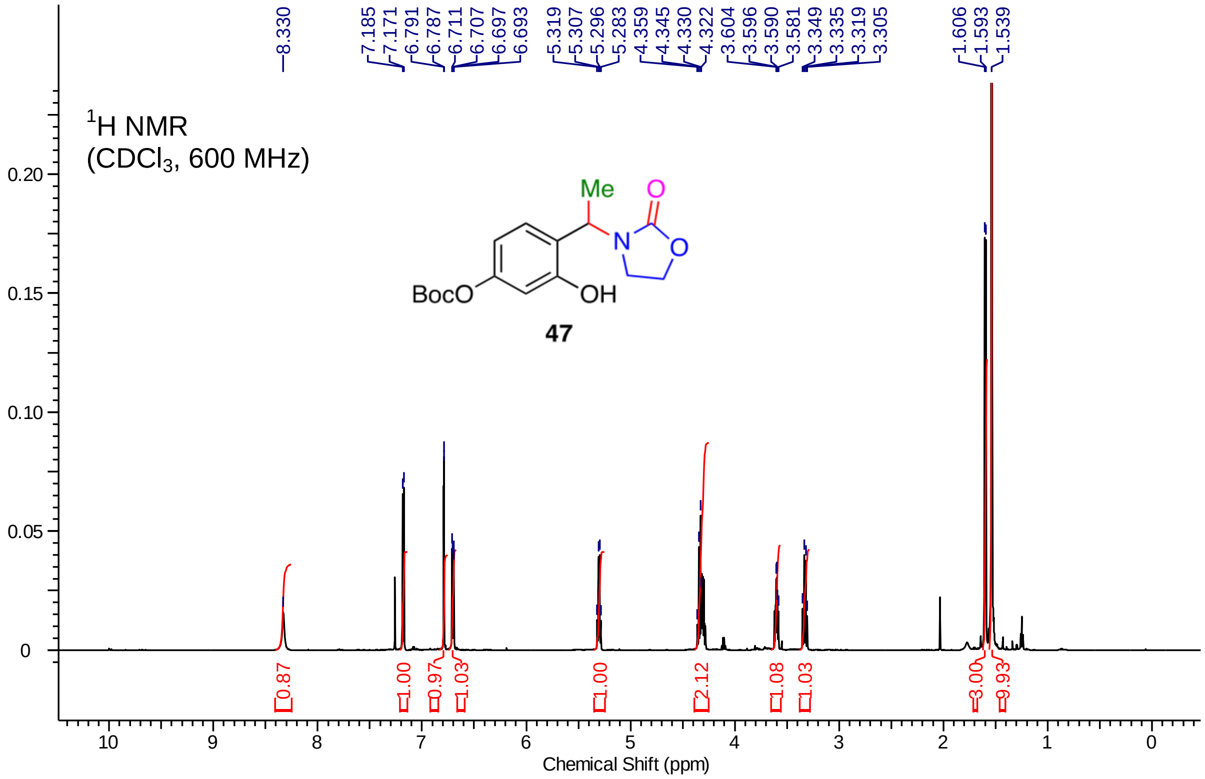

8.330
7.185
7.171
6.791
6.787
6.711
6.707
6.697
6.693
5.319
5.307
5.296
5.283
4.359
4.345
4.330
4.322
3.604
3.596
3.590
3.581
3.349
3.335
3.319
3.305
1.606
1.593
1.539
^{1}H NMR
($CDCl_3$, 600 MHz)
Me
O
N
O
BocO
OH
47
0.87
1.00
0.97
1.03
1.00
2.12
1.08
1.03
3.00
9.93
Chemical Shift (ppm)

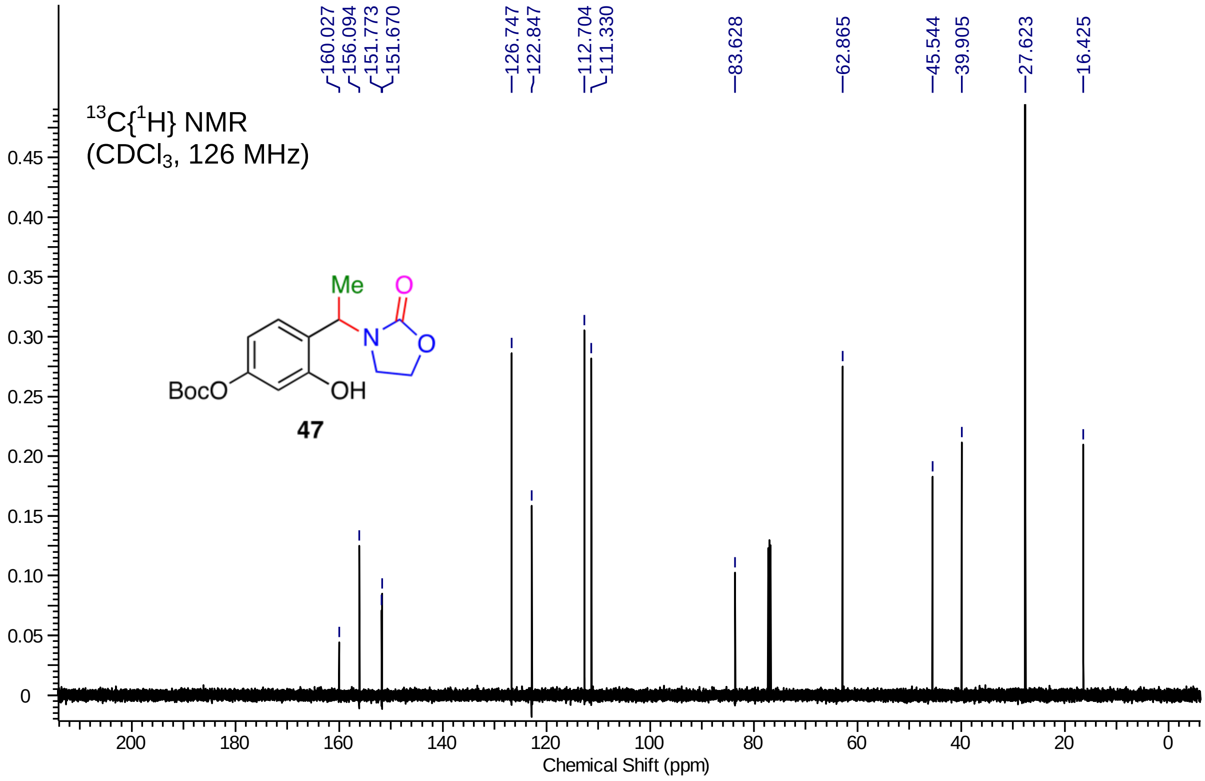

160.027
156.094
151.773
151.670
126.747
122.847
112.704
111.330
83.628
62.865
45.544
39.905
27.623
16.425
^{13}C{^{1}H} NMR
($CDCl_3$, 126 MHz)
Me
O
N
O
BocO
OH
47
Chemical Shift (ppm)

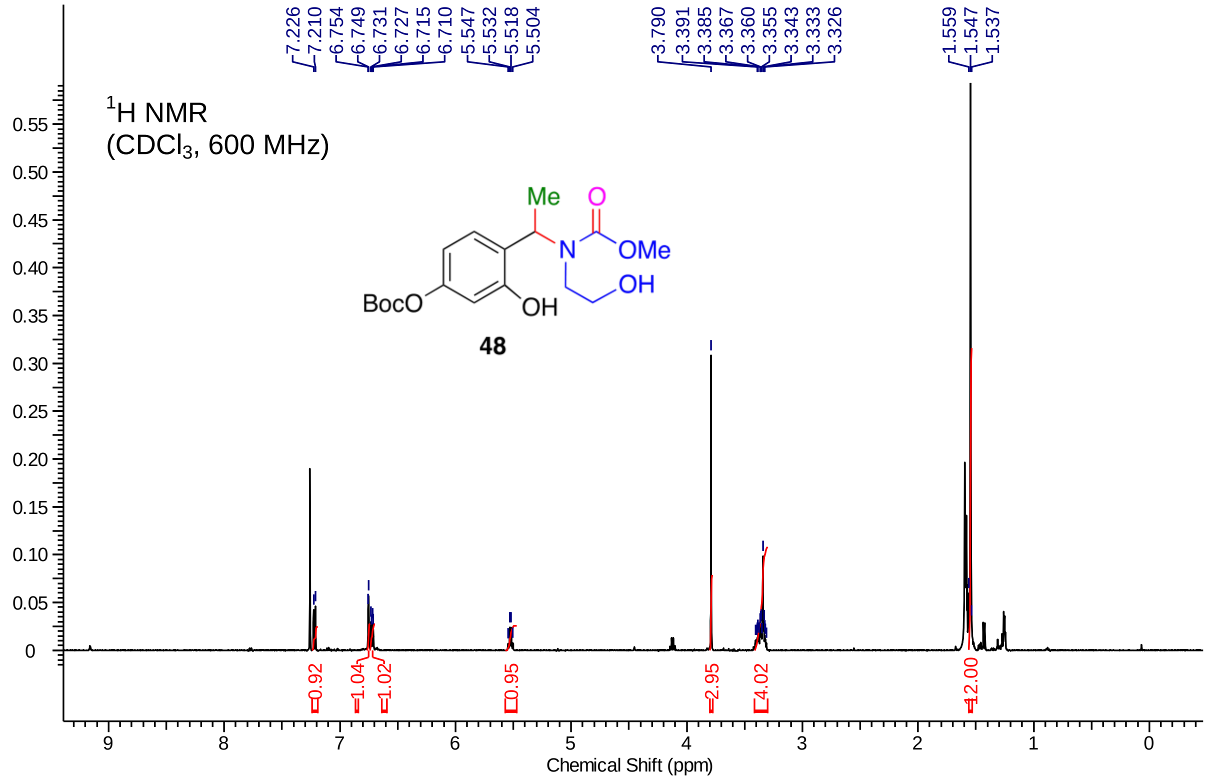

7.226
7.210
6.754
6.749
6.731
6.727
6.715
6.710
5.547
5.532
5.518
5.504
3.790
3.391
3.385
3.367
3.360
3.355
3.343
3.333
3.326
1.559
1.547
1.537
^{1}H NMR
(CDCl3, 600 MHz)
Me
O
N
OMe
OH
BocO
OH
48
0.92
1.04
1.02
0.95
2.95
4.02
12.00
Chemical Shift (ppm)

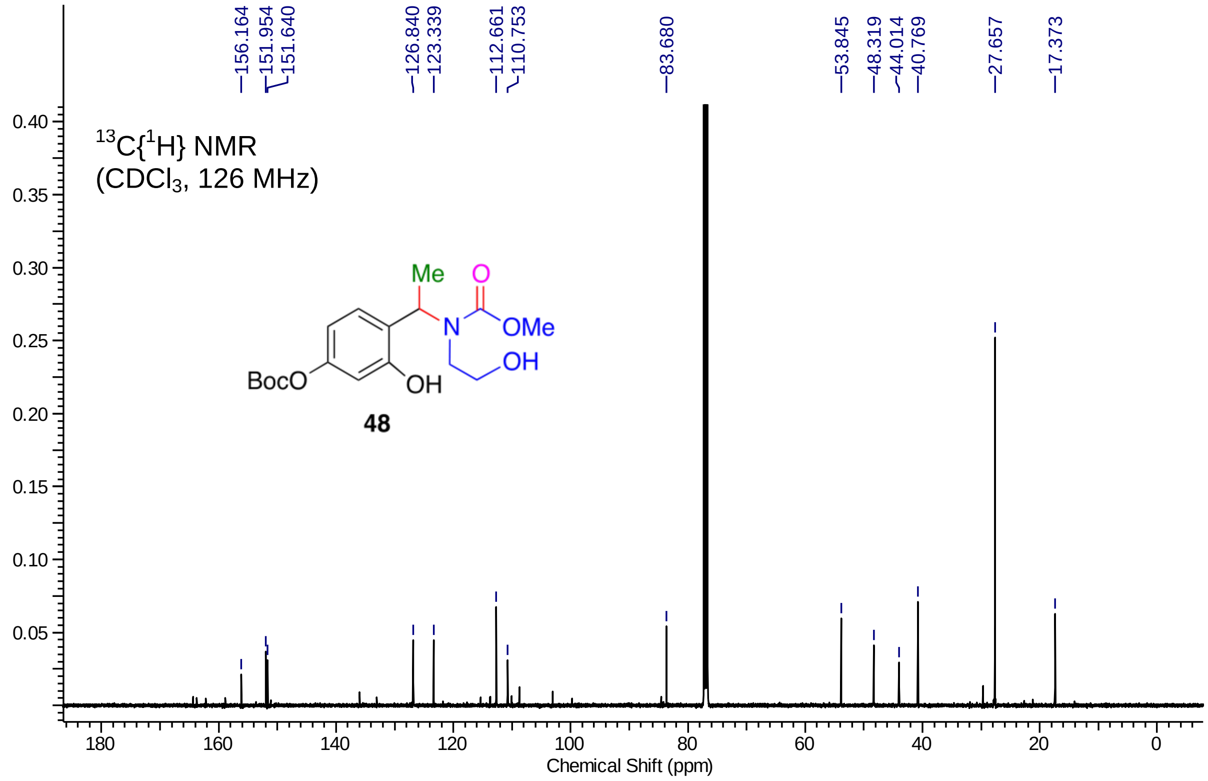

156.164
151.954
151.640
126.840
123.339
112.661
110.753
83.680
53.845
48.319
44.014
40.769
27.657
17.373
^{13}C{^{1}H} NMR
(CDCl3, 126 MHz)
Me
O
N
OMe
OH
BocO
OH
48
Chemical Shift (ppm)

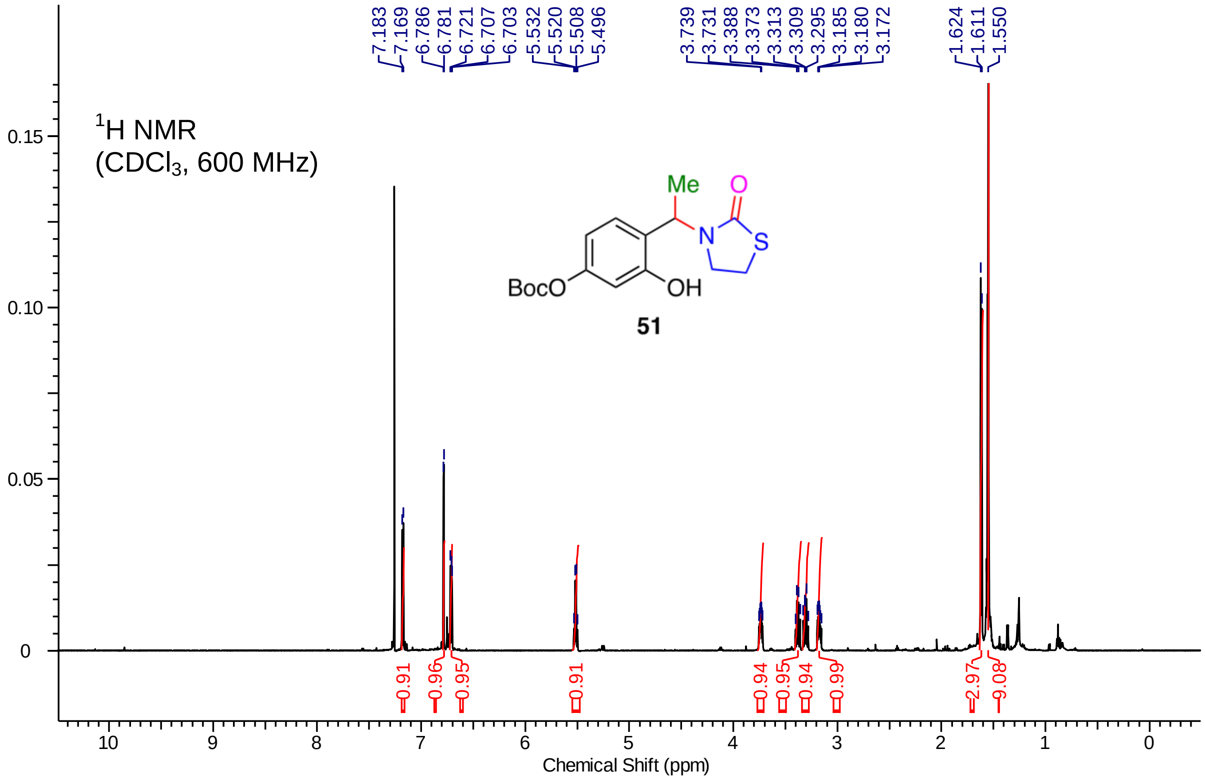
7.183
7.169
6.786
6.781
6.721
6.707
6.703
5.532
5.520
5.508
5.496
3.739
3.731
3.388
3.373
3.313
3.309
3.295
3.185
3.180
3.172
1.624
1.611
1.550
^{1}H NMR
($CDCl_3$, 600 MHz)
Me
O
N
S
BocO
OH
51
0.91
0.96
0.95
0.91
0.94
0.95
0.94
0.99
2.97
9.08
Chemical Shift (ppm)

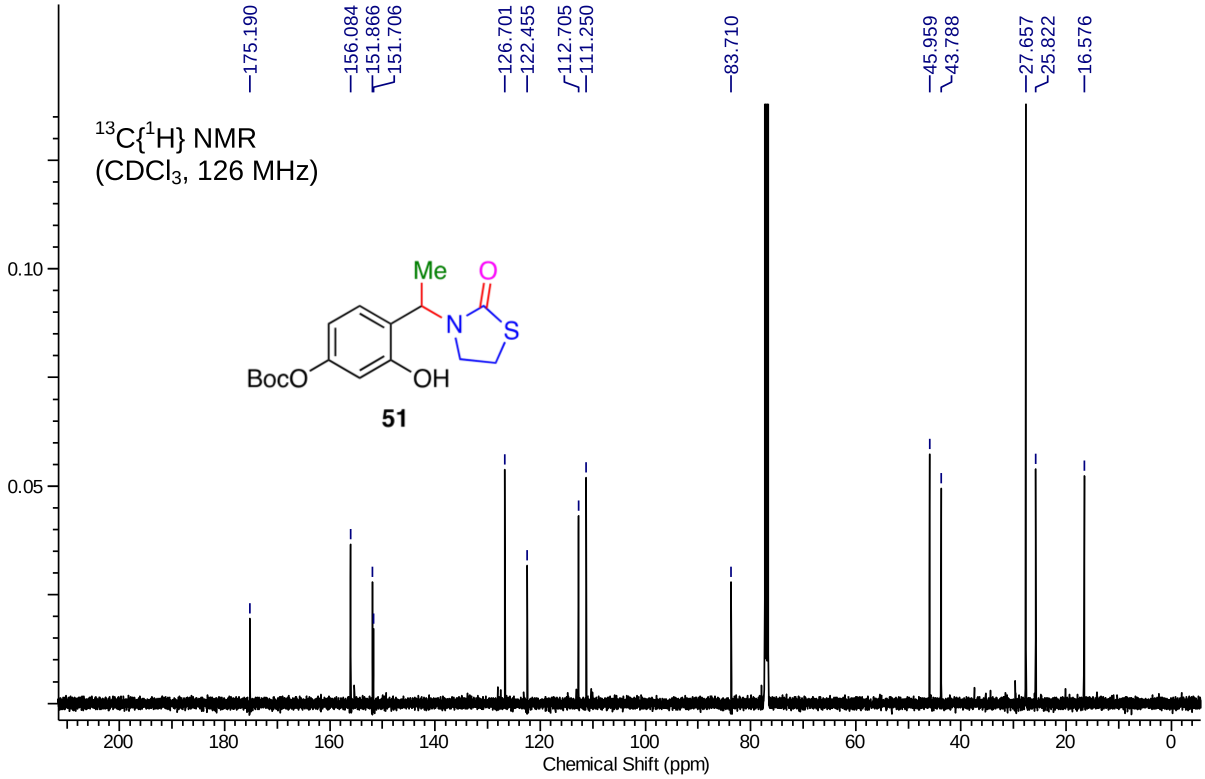
175.190
156.084
151.866
151.706
126.701
122.455
112.705
111.250
83.710
45.959
43.788
27.657
25.822
16.576
^{13}C{^{1}H} NMR
($CDCl_3$, 126 MHz)
Me
O
N
S
BocO
OH
51
Chemical Shift (ppm)

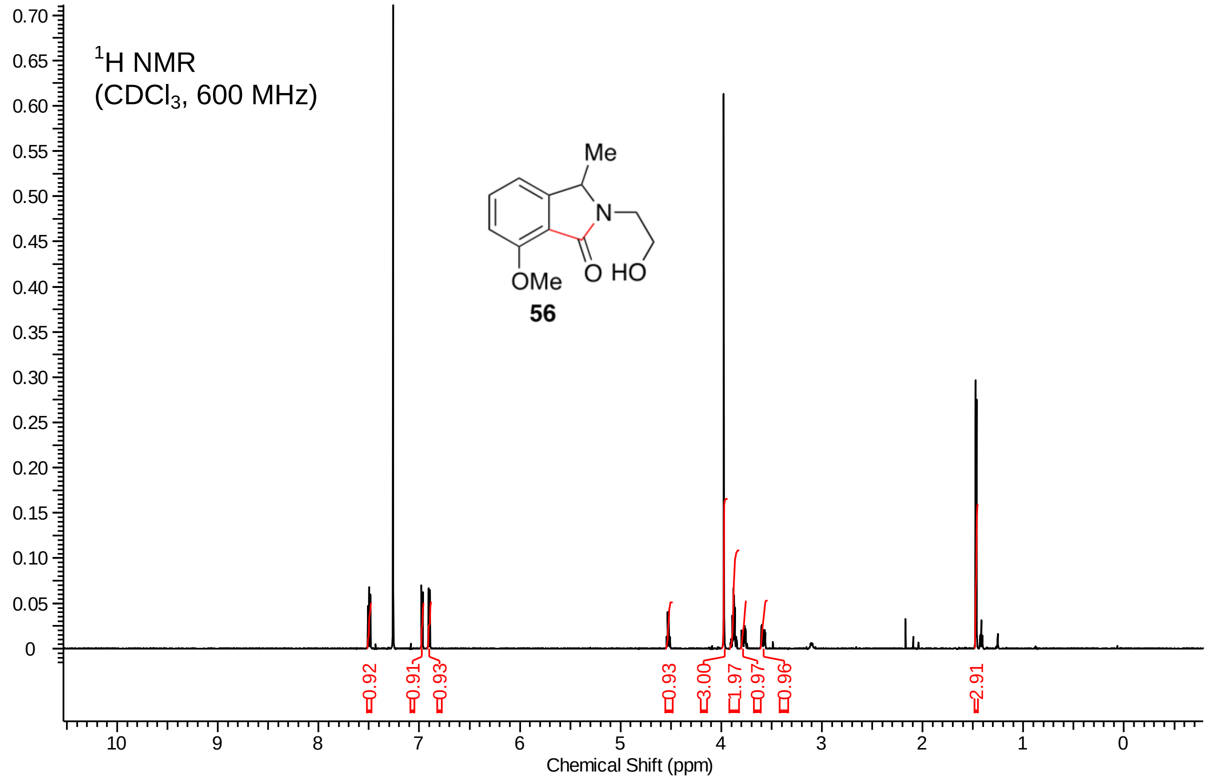

^{1}H NMR
(CDCl$_3$, 600 MHz)
Me
N
OMe
O
HO
56
0.92
0.91
0.93
0.93
3.00
1.97
0.97
0.96
2.91
Chemical Shift (ppm)

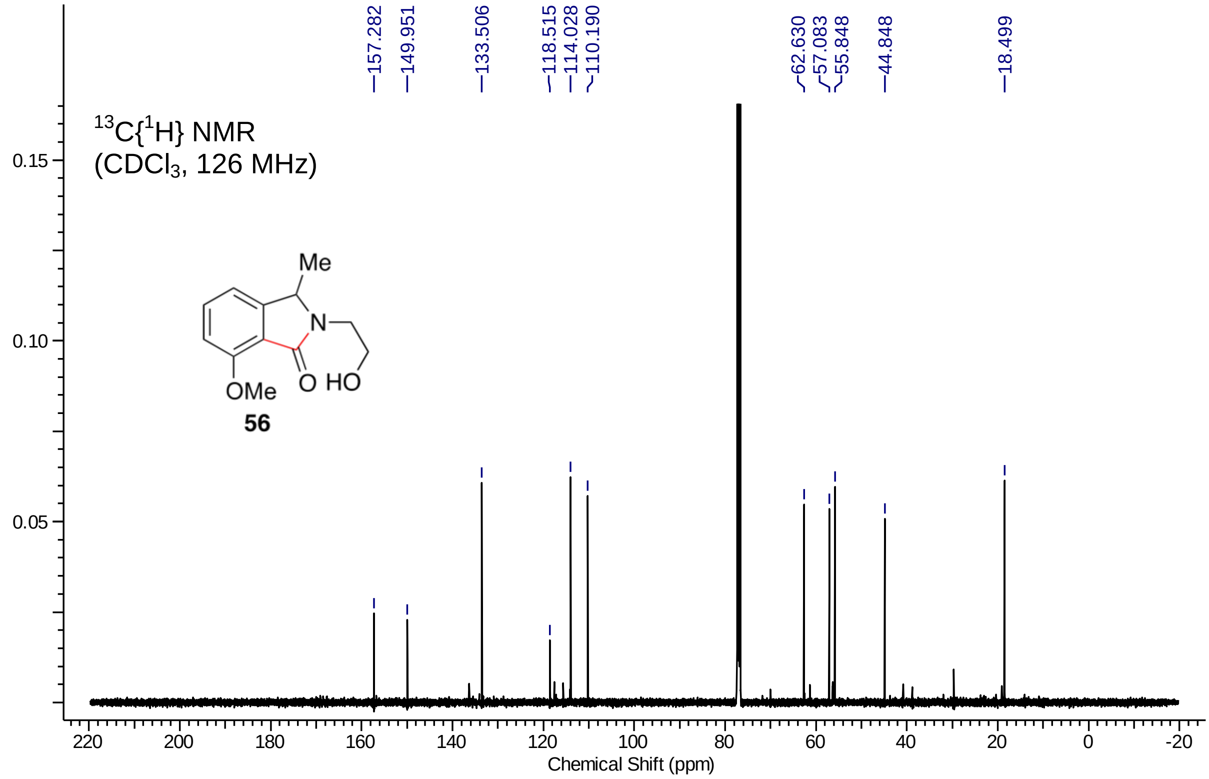

157.282
149.951
133.506
118.515
114.028
110.190
62.630
57.083
55.848
44.848
18.499
^{13}C{^{1}H} NMR
(CDCl$_3$, 126 MHz)
Me
N
OMe
O
HO
56
Chemical Shift (ppm)
